# Targeting Notch to Maximize Chemotherapeutic Benefits: Rationale, Advanced Strategies, and Future Perspectives

**DOI:** 10.3390/cancers13205106

**Published:** 2021-10-12

**Authors:** Nadezda Zhdanovskaya, Mariarosaria Firrincieli, Sara Lazzari, Eleonora Pace, Pietro Scribani Rossi, Maria Pia Felli, Claudio Talora, Isabella Screpanti, Rocco Palermo

**Affiliations:** 1 Department of Molecular Medicine, Sapienza University of Rome, 00161 Rome, Italy; nadezda.zhdanovskaya@uniroma1.it (N.Z.); mariarosaria.firrincieli@uniroma1.it (M.F.); sara.lazzari@uniroma1.it (S.L.); pace.1734142@studenti.uniroma1.it (E.P.); scribanirossi.1793423@studenti.uniroma1.it (P.S.R.); claudio.talora@uniroma1.it (C.T.); 2 Center for Life Nano Science, Istituto Italiano di Tecnologia, 00161 Rome, Italy; 3 Department of Experimental Medicine, Sapienza University of Rome, 00161 Rome, Italy; mariapia.felli@uniroma1.it

**Keywords:** Notch, oncogene, tumor suppressor, cancer treatment, chemotherapy, drug combinations

## Abstract

**Simple Summary:**

The Notch signaling pathway regulates cell proliferation, apoptosis, stem cell self-renewal, and differentiation in a context-dependent fashion both during embryonic development and in adult tissue homeostasis. Consistent with its pleiotropic physiological role, unproper activation of the signaling promotes or counteracts tumor pathogenesis and therapy response in distinct tissues. In the last twenty years, a wide number of studies have highlighted the anti-cancer potential of Notch-modulating agents as single treatment and in combination with the existent therapies. However, most of these strategies have failed in the clinical exploration due to dose-limiting toxicity and low efficacy, encouraging the development of novel agents and the design of more appropriate combinations between Notch signaling inhibitors and chemotherapeutic drugs with improved safety and effectiveness for distinct types of cancer.

**Abstract:**

Notch signaling guides cell fate decisions by affecting proliferation, apoptosis, stem cell self-renewal, and differentiation depending on cell and tissue context. Given its multifaceted function during tissue development, both overactivation and loss of Notch signaling have been linked to tumorigenesis in ways that are either oncogenic or oncosuppressive, but always context-dependent. Notch signaling is critical for several mechanisms of chemoresistance including cancer stem cell maintenance, epithelial-mesenchymal transition, tumor-stroma interaction, and malignant neovascularization that makes its targeting an appealing strategy against tumor growth and recurrence. During the last decades, numerous Notch-interfering agents have been developed, and the abundant preclinical evidence has been transformed in orphan drug approval for few rare diseases. However, the majority of Notch-dependent malignancies remain untargeted, even if the application of Notch inhibitors alone or in combination with common chemotherapeutic drugs is being evaluated in clinical trials. The modest clinical success of current Notch-targeting strategies is mostly due to their limited efficacy and severe on-target toxicity in Notch-controlled healthy tissues. Here, we review the available preclinical and clinical evidence on combinatorial treatment between different Notch signaling inhibitors and existent chemotherapeutic drugs, providing a comprehensive picture of molecular mechanisms explaining the potential or lacking success of these combinations.

## 1. General Aspects of the Notch Pathway

Notch signaling is an evolutionally conserved cell-to-cell communication mechanism that is critically involved in the regulation of an array of cellular processes during embryonic development and adult tissue homeostasis. Notch relays instruction from the surface to the nucleus of adjacent cells through the interaction of type I transmembrane ligands and receptors. In mammals, the Notch pathway consists of four receptors (Notch1–4) and five Delta/Serrate/Lag2 ligands (DSL), which belong to Delta-like (DLL1, DLL3, and DLL4) and Serrate-like (Jagged1 and Jagged2) families.

**Figure 1:**
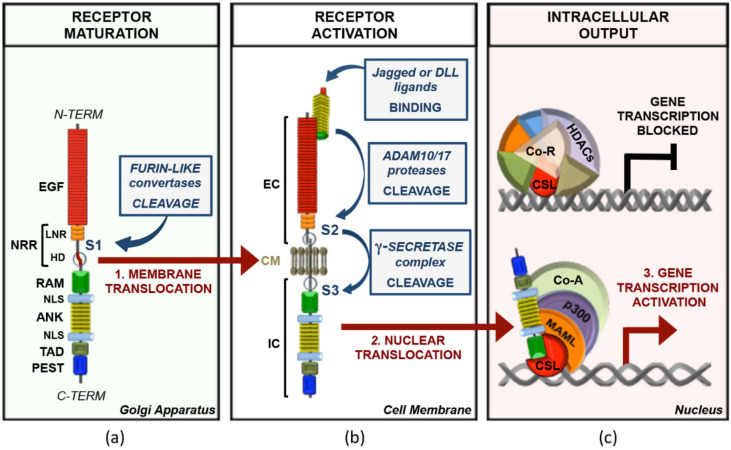
Main framework of canonical Notch signal transduction. (**a**) Notch receptors are synthetized as monomeric precursor proteins, which are subjected to a proteolytic cleavage by furin-like convertases (S1) in the Golgi apparatus before being exposed to the cell membrane as non-covalently linked heterodimers. From the N- to the C-terminal, the mammalian Notch proteins comprises: EGF (epidermal growth factor-like repeats), NRR (negative regulatory region), LNR (Lin12/Notch repeats), HD (heterodimerization domain), RAM (RBP-jk associated molecule), NLS (nuclear localization signal), ANK (ankyrin repeats), TAD (transactivation domain), and PEST (proline, glutamic acid, serine, and threonine). (**b**) The interaction of a Jagged or DLL (Delta-like ligand) family ligand to the EC (extracellular fragment) of the trans-membrane Notch receptor leads to the S2 cleavage of the receptor by ADAM10/17 (a disintegrin and metalloproteinase) and the subsequent S3 proteolysis catalyzed by the γ-secretase complex. This last cleavage releases from the membrane the IC (intracellular fragment) of Notch, which translocates to the nucleus. (**c**) In the absence of Notch, the transcription factor CSL (CBF-1/SuH/Lag-1 DNA-binding protein), in association with several Co-R (co-repressors factors) and HDACs (histone deacetylases) on the regulatory regions of Notch target genes, acts as a transcriptional repressor. The binding of the Notch IC to CSL displaces from CSL the Co-R, and by recruiting MAML (Mastermind-like), p300, and distinct context-related Co-A (co-activators factors), target genes’ transcription is switched to an activated state.

*Notch* genes encode large 300 kDa precursors that undergo post-translational modifications including a furin-like convertase-dependent S1 cleavage in trans-Golgi network necessary for its maturation into a multi-domain protein comprising an extracellular domain (NEC) noncovalently bound to a transmembrane-intracellular fragment (NTM) [1]. NEC is exposed on the cell surface and is responsible for the receptor/ligand interaction through epidermal growth factor (EGF)-like repeats [2]. This domain is followed by the negative regulatory region (NRR), which encompasses three cysteine-rich Lin12-Notch repeats (LNRs, defined as A, B, and C) and a heterodimerization domain (HD) (Figure 1) [3].

In absence of ligands, NRR restrains Notch signaling by hindering the ADAM10/17 (a disintegrin and metalloproteinase)-dependent proteolytic S2 sites that lie at the extracellular region of the NTM. Ligand binding causes the stretching of the NRR structure that unmasks S2 site and allows the ADAM10 metalloprotease-dependent proteolysis of the receptor. This process generates an intermediate transmembrane fragment termed Notch extracellular truncation (NEXT), which is rapidly cleaved by the intramembrane protease γ-secretase complex (GS) at S3 proteolytic site included in the NHD (Figure 1) [4,5,6]. GS-dependent proteolysis frees from the membrane the Notch intracellular domain (NIC), containing a single RAM (RBP-jκ associated molecule) domain, seven ankyrin repeats (ANK), a transactivation domain (TAD), and a PEST (proline (P)/glutamic acid (E)/serine (S)/threonine (T)-rich motif) sequence at the carboxy-terminus (Figure 1). NIC is translocated into the nucleus, where it associates with the DNA-binding CSL transcription factor Su(H) (also termed RBP-jκ (recombination signal binding protein for immunoglobulin kappa J region)) through its ANK domain at proximal promoters or distal enhancers of responsive genes [5,7,8]. ANK/CSL binds the N-terminal helix of coactivator Mastermind (MAM, MAML1-3 in mammals), which recruits p300/CBP histone acetyltransferases and other factors, finally assembling the Notch transcription complex (NTC) that turns on the Notch-driven transcription programs (Figure 1) [9]. NTC downstream factors include the universal Notch-targets Hairy/Enhancer of Split (HES) family genes (*HES1*, *HES5*, and *HEY1*) and several lineage-specific genes such as *CYCLIND1*, *NRARP*, *PTCRA*, *MYC*, and others [10].

Besides the above-mentioned canonical system machinery of Notch signaling, Notch can operate by alternative modalities. Indeed, the signal of Notch receptors could be elicited by non-canonical ligands (i.e., by MAGP1, MAGP2, and DLK1) [11,12], or it could promote gene transcription in a CSL-independent manner (i.e., IL6) [13]. In addition, a ligand-independent mechanism of Notch processing was unveiled in activated CD4^+^ T cells, according to which, in the absence of ligand binding, the T-cell receptor (TCR)/CD28 signaling pathway stimulated the internalization of Notch receptor in the endosome compartment and triggered its processing by ADAM metalloproteases via protein kinase C (PKC) activation [14,15]. A similar mechanism was described in CD8+ T-cells, in which the stimulation of the adenosine A2A receptor (A2AR) decreased the processing and signaling of the Notch1 receptor by interfering with the early phases of the TCR signaling transduction [16,17]. Moreover, a DSL-independent mechanism of Notch activation was driven by sphingosine 1-phosphate receptor 3 (S1PR3) in breast cancer, and it was involved in the expansion of a CSCs population [18].

Of note, in absence of NIC, RBP-jκ acts as a repressor, interacting with the corepressor complexes such as SMRT/mSin3A/HDAC1, NCor/mSin3A/HDAC1, or CIR/SAP30/HDAC2. Another corepressor complex is composed of RBP-jκ, SHARP (SMRT and HDAC1 associated repressor protein), CtBP (C-terminal binding protein), and CtIP (CtBP interacting protein) (Figure 1c). CtBP in its turn forms a complex with a histone demethylase LSD1/CoREST [10]. Several inactivating mechanisms allow fine dosing of the activated signal providing well-timed degradation of NIC. MAML can recruit different kinases such as the cyclin C/cyclin-dependent kinase 8 complex and glycogen synthase kinase 3β (GSK-3β), which target PEST domain [19]. PEST phosphorylation leads to NIC ubiquitination and proteasomal degradation, allowing the cell to start a new cycle of ligand-dependent Notch activation [20]. This step is mediated by the E3 ubiquitin ligase SCF_Fbxw7_ (S-phase-kinase-associated protein1(SKP1)-cullin1(CUL1)-F-box) protein complex, which is responsible for the recruitment of an F box protein involved in NIC degradation (FBXW7) [20]. Mammals have three isoforms of FBXW7 (α, β, and γ) generated by alternative splicing and different in their 5′-UTR and N-terminal coding regions. FBXW7α is expressed in the nucleoplasm and provides NIC degradation [11,13,21].

## 2. Notch Signaling Physiological Functions

Although Notch paralogues share similar structure and pathway architecture, Notch signaling generates different and even opposite cellular responses in cell developmental state- and lineage-dependent ways, thus finely governing cell fate and differentiation in a broad variety of tissues. To make Notch’s simplicity a little more complicated, in addition to Notch receptors, the distinct ligands could also promote different cellular outcomes, even inhibiting receptor activation if expressed on the surface of the same cell (cis-inhibition).

Mechanisms underlying the context-dependent selection of target genes by Notch are widely investigated yet are still mostly poorly understood. In embryonic development and tissue homeostasis, Notch signaling plays a crucial role in organ and tissue development thanks to its capacity to guide cell fate, leading to cell differentiation or maintaining self-renewal in a context-dependent way. Notch-orchestrated balancing between these events contributes to the maintenance of tissue homeostasis (reviewed in [22]).

Starting from somitogenesis, Notch signaling cooperates with other pathways such as Wnt, and its oscillatory expression drives somite segmentation and regulates the formation of somite-derived organs such as vertebral column and skeletal muscles [23]. In line with this, dysregulated expression of Notch ligands and its target genes has been associated with aberrant morphology of the vertebral column, whereas in myogenesis, Notch orients cell fate towards endothelial or smooth muscle phenotype (reviewed in [24]). In cardiac muscle development, Notch pathway activation blocks cardiomyocyte differentiation and supports the choice of non-myocardial cell fates [25]. Indeed, Notch signaling is crucial for the correct development of endocardial structures such as valves and chamber endocardium and for the genesis of epicardium and epicardium-derived coronary vessels [26]. Notch signaling influences not only coronary vessel formation but vasculature in general, where its crosstalk with vascular endothelial growth factor (VEGF) becomes particularly important [24]. In fact, VEGFR-mediated signaling upregulates DLL4 expression, which in its turn inhibits VEGF signaling (reviewed in [27]). Moreover, DLL4 overexpression leads to inefficient angiogenesis with defective endothelial tip formation and vessel branching, whereas Jagged1-activated Notch signaling has a weaker potency, competes with DLL4, and works in a proangiogenic way [28,29]. Interestingly, Notch signaling contributes to cardiac regeneration after injury, controlling the balance between fibrotic and regenerative repair in the adult heart [30].

Notch’s importance for differentiation and cell fate decisions is evident also in embryonic development of neural tissue [31,32]. The feed-forward and the feed-back regulatory loops involving Notch and its target genes and HES/proneural factor oscillations are crucial for neural stem cell maintenance and proliferation as well as normal timing of neurogenesis [33,34,35]. Indeed, Notch1, Notch2, Notch3, and the genes of *HES/HEY* family regulate the self-renewal of neural stem cells (NSCs) in radial glia by balancing their quiescence and commitment during embryonic and early development stages [36,37]. Notch1 and Notch3 appear to act similarly in the developing brain; however, the functional outcome of different transcriptional targets of Notch may differ, as HES5/CBF1 promoted radial glial/progenitor character of NSC commitment, whereas neurosphere growth was apparently independent of them [38]. The role of Notch signaling in peripheral nervous system formation is not completely clear, but it may be related to neural crest development (reviewed in [24]). However, the role of Notch signaling is not limited to the developing brain, since the contrasting activity of Notch paralogues and variable effects of Notch target genes contribute to adult NSC heterogeneity and regulate the balance between their quiescence and activation [32]. Notch1 is required to maintain a reservoir of undifferentiated cells in the adult hippocampus, and its loss results in self-renewal failure of adult NSCs with a consequent block of the transition from the quiescent to actively proliferating state [39,40]. On the other hand, Notch2 and Notch3 provide a maintenance signal for quiescence in adult NSCs, preventing cell cycle progression and differentiation [40]. Notch2 regulates the quiescence of ventricular-subventricular zone NSCs, and its effectors block cell-cycle entry. Indeed, the loss of Notch2 stimulated NSC to proliferate and generate new neurons resulting in accelerated exhaustion of NSC pool, whereas the loss of Notch2 target gene Id4 activated NSC proliferation and promoted astrogliogenesis and not neurogenesis [41,42]. Moreover, Notch3 gates NSC activation and amplification, and Notch3 deletion preferentially reduced the population of quiescent NSC in the lateral and ventral walls of the lateral ventricles [43,44].

Notch signaling participates in gastrointestinal tract development, being particularly important yet controversial for pancreatic organogenesis with evidence supporting its negative impact on early endocrine and ductal lineage differentiation and contribution to progenitor cell maintenance and the acinar cell fate choice [45,46,47,48]. In liver development, Notch signaling can block the differentiation of hepatoblasts into hepatocytes and favor the choice of cholangiocyte fate; however, the evidence regarding the expression of Notch receptors on proliferating bile ducts remains ambiguous [49,50,51]. Moreover, some receptors may be involved in neovascularization [50]. The indisputable yet not completely clear role of Notch signaling in hepatic progenitor differentiation supports its participation in liver repair after injury and creates a possible background for the development of hepatoblastoma, cholangiocarcinoma, and hepatocellular carcinoma (HCC) [52]. Finally, in intestinal development, Notch maintains the undifferentiated state of crypt progenitors in cooperation with the Wnt pathway to provide the expansion of immature cells, whereas the blocking of Notch signaling favors the choice of goblet cell fate [53].

Notch signaling plays a pivotal role in lung development, and different Notch receptors and ligands are abundantly and specifically expressed in epithelial, mesenchymal, and endothelial elements of the embryonic lung starting from the early stages, and they participate in proximo-distal differentiation of the airway epithelium, alveologenesis, and cell phenotype switch [1,2,3]. Interestingly, in this case, Notch shifts the balance between ciliated, secretory, and neuroendocrine cells to the secretory phenotype and supports smooth vascular cells expansion, and its ablation may lead to the overpopulation of ciliated cells and the expansion of neuroendocrine bodies [4,5,6].

Notch signaling has a well-recognized role in embryonic hematopoiesis, supporting the generation of hematopoietic stem cells (HSC) from the endothelium during embryogenesis, whereas its impact on the maintenance of post-embryonal HSC remains controversial, with evidence supporting its participation in HSC self-renewal and communication with their niche and its dispensable contribution to megakaryocyte and erythrocyte progenitors’ differentiation [54,55,56,57,58]. At the same time, Notch signaling is known to be crucial for correct lymphoid differentiation and intrathymic development of T lymphocytes cooperating with other transcription factors (TFs) and controlling αβ- and γδ-transition stages as well as preTCR and TCR genes [59,60,61,62].

The contribution of Notch signaling to the epidermal homeostasis is based on the spatial and sequential activity of different Notch ligands, receptors, and downstream effectors such as p21 (Waf1/Cip1) and p63, orchestrating epidermal differentiation and proliferation [63,64,65]. More commonly, Notch signaling contributes to the commitment switch in epithelial lineage, and its activation results in growth arrest and terminal differentiation. At the same time, the contribution of different receptors and ligands may differ, as DLL1 may support the undifferentiated state of keratinocytes, whereas Jagged1 contributes to hair follicle differentiation [66,67,68,69].

These findings support the importance of Notch signaling in both developing and adult tissues’ homeostasis and suggest that Notch deregulation is tightly linked to the onset and progression of several congenital diseases and cancer [70,71].

## 3. The Double-Faced Notch in Cancer

**Figure 2:**
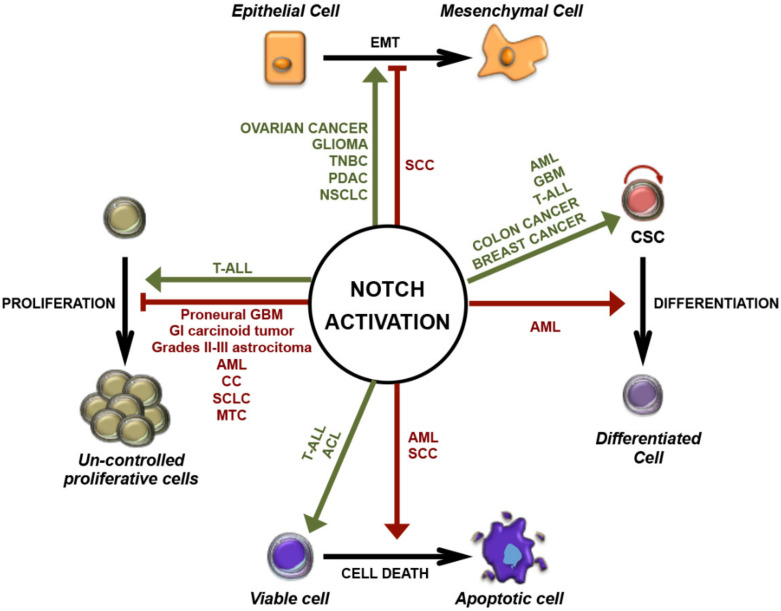
Pleiotropic functions of Notch activation in cancer. Schematic representation of oncogenic (green) and tumor-suppressive (red) roles of Notch signaling in different cancers: stimulation or inhibition of uncontrolled proliferation; regulation of epithelial-mesenchymal transition (EMT); induction of differentiation or maintenance of cancer stem cells (CSCs); promotion of cell survival or cell death. ACL: lung adenocarcinoma; AML: acute myeloid leukemia; CC: cervical cancer; GI: gastrointestinal; GBM: glioblastoma multiforme; MTC: medullary thyroid carcinoma; NSCLC: non-small-cell lung cancer; PDAC: pancreatic ductal adenocarcinoma; SCLC: small-cell lung cancer; SCC: squamous cell carcinoma; T-ALL: T-cell acute lymphoblastic leukemia; TNBC: triple-negative breast cancer.

The aberrant activation of Notch signaling generally supports cancer development. However, in line with its pleiotropic function, it has been shown to exert the opposite role and to prevent carcinogenesis in certain tissues (summarized in Figure 2) [72,73].

### 3.1. Mechanisms of Notch Signaling Alteration in Cancer

Initially, *Notch* mutations were associated with a small subset of T-cell acute lymphoblastic leukemia (T-ALL) patients carrying the chromosomal translocation t(7;9)(q34;q34.3) that, by fusing the 3′ end of NRR-truncated *Notch1* gene with the enhancer element of *TCRβ* gene, leads to constitutive and ligand-independent activation of Notch1 [74]. Further sequencing analysis revealed that up to 50% of T-ALL patients harbor mutations at the coding sequence of the *Notch1* gene, which by disrupting the NRR and/or PEST domains results in hyper-activated signaling due to increased receptor susceptibility to ADAM cleavage or NIC half-life, respectively [75]. In addition to *Notch1*, a similar pattern of mutations occurs at other *Notch* genes in several types of hematological malignancies and solid tumors, such as chronic lymphocytic leukemia (CLL) [76], B-cell malignancies [77,78,79,80], triple-negative breast cancer (TNBC) [81], adenoid cystic carcinoma (ACC) [82], and non-small-cell lung cancer (NSCLC) [83]. Moreover, hyper-activated Notch has been related to missense mutations at the *FBXW7* coding sequence in several hematological malignancies, including approximately 30% of T-ALL patients [84]. Mechanistically, loss-of-function mutations in the *FBXW7* gene, by preventing the FBXW7-mediated NIC degradation, extend its half-life and amplify the output of the signal. Interestingly, *FBXW7* mutations have been also linked to γ-secretase inhibitor (GSI) resistance in T-ALL [85,86].

In addition to the genetic alterations that lead to oncogenic activation of Notch in several cancers, loss-of-function mutations harbored in the genes encoding Notch pathway components have been detected in certain subgroups of tumors. Indeed, mutations, occurring at the genes of the γ-secretase component, the co-activator *MAML1*, as well as the Notch receptors that lead to reduced Notch signaling, have been described in particular subclasses of patients with chronic myelomonocytic leukemia [87], bladder cancer [88], low-grade glioma [89], and small-cell lung cancer (SCLC) [90]. Moreover, *Notch* genes inactivating mutations are among the most frequent genetic alteration in patients of squamous cell carcinoma (SCC) of the esophagus (ESCC) [91], head and neck (HNSCC) [92], skin (SSCC), and lung (LSCC), thus indicating that Notch-deficiency is required for cancer onset and progression for these tissues [93].

Notch signaling deregulation in cancer may be also driven by non-mutational mechanisms influencing Notch receptors’ expression, stability, and activity. Accordingly, despite how few *Notch*3 gene alterations have been described, upregulated Notch3 signaling, due to aberrant post-translational modifications, epigenetic mechanisms, and abnormal activity of other Notch regulators, including non-coding RNA, has been linked to the pathogenesis of several cancers (reviewed in [94,95]). Furthermore, deregulation of oncogenes or tumor suppressors may modulate Notch signaling in a downstream way, as lack or inhibition of the negative regulator of NIC Numb has been linked to enhanced activity of Notch in breast cancer, glioblastoma multiforme (GBM), and NSCLC [83,96,97], while inactivation of p53 has been demonstrated to lead to up- or downregulation of Notch signaling in several cancers (reviewed in [98]).

### 3.2. Notch-Driven Carcinogenesis

Given the contrasting mutational and aberrant regulatory patterns of Notch signaling, the design of pharmacological anti-cancer strategies must consider the functional role and the pathway interaction of the distinct paralogs in the diverse tissues to drive appropriate therapeutic Notch-modulation. The best-characterized oncogenic function of Notch is realized through promoting cell growth and survival by inducing the specific transcriptional program in a context-dependent way. In T-ALL, the oncogene c-Myc is a direct transcriptional target of Notch1, crucial for controlling cell growth and metabolism [99]. Confirming the importance of this interaction, the exogenous expression of c-Myc rescued the anti-growth and pro-apoptotic effects of Notch-inhibition by GSI in Notch-dependent T-ALL in vitro and in vivo [100], and consistently, it has been shown that T-ALL resistance to Notch-targeting agents is due at least in part by the chromatin modifier BRD4 that epigenetically sustains c-Myc expression and function [100,101,102]. Supporting Notch–Myc crosstalk, c-Myc sustained Notch1 activity via suppressing its negative regulator microRNA (miR)-30 [103]. Of note, the turnover of Myc protein in T-ALL, and likewise NIC, is controlled by FBXW7-mediated proteasomal degradation; therefore, *FBXW7* loss-of-function mutations result in increased Myc protein levels and function [86]. Furthermore, Palomero and colleagues revealed the existence of a Notch1-PTEN-AKT axis among mechanisms that underlie Notch-dependent T-ALL leukemogenesis. The authors demonstrated that HES1, the direct transcriptional target of Notch1, by repressing the transcription of the oncosuppressor *PTEN* (phosphatase and tension homolog deleted on chromosome 10), elicits the activation of the pro-survival PI3K-AKT pathway [104]. Consistently, the mutational loss of the oncosuppressor PTEN results in the constitutive activation of AKT signal that stabilizes Myc by inhibiting its GSK-3β-dependent proteasomal degradation [105], making T-ALL cells resistant to Notch pharmacological inhibition [104]. In addition, Notch sustains T-ALL initiation and progression indirectly by means of its downstream target genes *IL7Ra* and *IGF1R*, which strongly contribute to AKT pathway activation [106,107]. Likewise, in lung adenocarcinoma (ACL), Notch/AKT crosstalk is regulated by IGF1R in a Notch-dependent way that sustains cell survival under hypoxia stimuli. Moreover, it has been shown that IGF1R inhibition enhanced pro-apoptotic effects of GSI, thus supporting the rationale for combinatorial IGF1R and Notch targeting in this type of cancer [108]. Moreover, Notch signaling sustains proliferation, survival, and invasion in several cancer cells by interacting with the pro-survival transcription factor nuclear factor κB (NF-κB). Indeed, evidence supports Notch-NF-κB crosstalk as one of the major mediators of Notch-driven T-ALL transformation via different mechanisms. In particular, in murine models of Notch3- and Notch1-dependent T-ALL, Notch signaling sustained the canonical p50/p65 NF-κB pathway by interacting with the IKK (Inhibitor of NF-κB (IκB) Kinase) signalosome, and consistently, independent studies demonstrated that combined pharmacological inhibition of Notch and NF-κB strongly enhanced cell growth arrest and apoptosis in T-ALL [109,110,111,112,113]. Otherwise, in colorectal cancer model, IKKα was recruited to regulatory regions of Notch target genes, leading to their uncontrolled transcription in an NF-κB independent way [114]. In triple-negative breast cancer and glioma, Notch activates NF-κB via AKT, and this axis sustains cell migration and invasion via regulation of invasion and epithelial-mesenchymal transition (EMT)- related genes [115,116]. EMT is associated with the loss of epithelial molecular markers, including E-cadherin, α-, and β-catenin, as well as an increase in mesenchymal markers, such as vimentin and fibronectin. Moreover, it is a fundamental process in embryonic development, tissue repair, and diseases including cancer, in which it underlies metastatic dissemination and drug resistance [117,118]. During cancer progression, EMT is triggered by the interplay of specific secreted factors including EGF, PDGF, TGFβ; transcription factors such as ZEB1, Slug, Snail; and signal pathways such as AKT, ERK, and Notch. Multiple studies have indicated the interplay between Notch and TGFβ as critical in induction and maintenance of EMT in solid tumors. TGFβ-dependent EMT in NSCLC and SCC is regulated by the interaction between EMT-related transcriptional factor ZEB1 and Notch signaling [119,120]. In particular, TGFβ induced the signaling of Notch3 in NSCLC and Notch1 in SCC that in turn are responsible for the transcriptional activation of ZEB1. However, in SCC, another necessary step in TGFβ-Notch1 induction of EMT is ZEB1-dependent inhibition of *Notch3*, suggesting that Notch1 and Notch3 may have different cancer-dependent roles in EMT induction and highlighting that the use of pan-Notch inhibitors may not be universally useful [120]. In breast and ovarian cancer cell models, the hypoxia/Notch/EMT axis has been described, in which Notch, under the hypoxia stimuli, upregulates the known EMT inducer Snail1 and downregulates the epithelial marker E-cadherin [121,122]. Additionally, Jagged2 may be overexpressed in bone marrow stroma under hypoxic conditions, providing conditions favoring the renewal of Notch-dependent cancer stem-like cells [122]. Cancer stem cells (CSC) are a subpopulation of cancer cells which are implicated in metastasis, recurrence after therapy, and drug resistance, the maintenance of which relies on several particular pathways including Hedgehog, Wnt, and Notch [123]. Emerging evidence suggests that CSCs concentrate in perivascular regions and that tumor-associated endothelial cells (ECs) could maintain CSCs through direct activation of Notch signaling, which, in case of colon cancer, may be achieved through paracrine release of soluble Jagged1 [124]. Moreover, in glioblastoma, Notch blockade attenuated CSC renewal through affecting the ECs of the vascular niche and increased the efficiency of radiotherapy [125]. Notably, positive staining of glioblastoma for HEY1 and Notch1 correlated with worse prognosis of patients and resistance to chemo- and radiotherapy [126,127]. Confirming the oncogenic role of Notch in glioma stem cells (GSC), Notch inhibition with GSI reduced cell proliferation and induced neural differentiation of GSC by upregulating ASCL1 expression, high levels of which were associated with elevated neuronal lineage potential and good response to Notch inhibition [128]. Furthermore, in Wnt-dependent glioblastoma positive for ASCL1-high GSC cells, the combined treatment with GSI and the Wnt inhibitor LGK974 enhanced the anticlonogenic and neural prodifferentiative potential of GSC compared with the GSI alone. The concomitant prodifferentiative action of Wnt and Notch inhibition in tumors with high expression of ASCL1 has also been suggested [129]. These studies highlight the oncogenic role of Notch-mediated suppression of ASCL1; however, its Notch-dependent inhibition can also result in a tumor-suppressive effect by interfering with the proliferation of glioma cells [130]. In line with the bivalent role of Notch in brain tumors, simultaneous inhibition of Notch signaling and p53 in glioma murine models induced the formation of aggressive sPNET-like (supratentorial primitive neuroectodermal tumor) brain tumors. Consistently low levels of HES5 correlated with poor prognosis in proneural GBM and grades II-III astrocytoma patients [131].

The distinct function of Notch pathway members in the regulation of the self-renewal and activity of CSCs has been widely investigated in breast cancer. For instance, high levels of Notch1 have been correlated with tumor progression, unfavorable survival and disease recurrence in patients with malignancies of the breast [132,133,134,135]. Indeed, Notch1 signaling sustained the survival of a CSC-enriched population following the inhibition of mTOR pathway in TNBC cell models [136], and likewise, it maintained the self-renewal of a population of CSCs resistant to the trastuzumab-based therapy in HER2-positive breast cancer by repressing PTEN [137]. On the other hand, Notch3 signaling mediated spontaneous lung metastasis in estrogen receptor alpha-positive (ERα+) breast tumor xenografts by sustaining the self-renewal and high invasive properties of a population of metastatic cancer cells [138]. Moreover, Notch3 critically regulated self-renewal and survival of the mammary gland stem/progenitor cells derived from ductal breast carcinoma patients under hypoxic conditions through the interplay with the 66-kDa isoform of the *SHC* gene (p66Shc) [139]. Concerning Notch4 in breast cancer, it is required for CSC maintenance, and its high levels have been documented in patient samples. Moreover, its signaling activity correlated with the recurrence following the chemo- and endocrine therapy [140,141]. Indeed, the aberrant activity of Notch4 signaling has been described in CSC-like cells isolated from cancer cell lines and patients’ primary samples, and its selective inhibition by shRNA significantly affected the mammosphere formation and tumor initiation capabilities [142]. Likewise, a Notch4-neutralizing antibody reduced the tumorsphere-forming efficiency of cancer cells isolated from primary ductal carcinoma in situ, thus suggesting that Notch4 disruption would be therapeutically useful for the treatment of this cancer [143]. The mechanism of Notch4-mediated resistance to hormonal therapy of breast cancer is worth mentioning. Indeed, the treatment with tamoxifen or fulvestrant in patient-derived samples and xenograft models of ER+ breast tumor selected a population of CSC-like cells through the activation of the Jagged1-Notch4 signaling. Confirming the key role of Notch4 in endocrine resistance, the combinatory treatment with Notch inhibitors reduced the frequency of the hormonal therapy-resistant CSC [144]. Additionally, the treatment with an FK506-binding protein-like (FKBPL)-based peptide repressed a subpopulation of endocrine therapy-resistant CSC in ER+ breast cancer by interfering with DLL4 and Notch4 signaling [145]. Moreover, mutational disruption of *ERα* ligand-binding domain (which frequently occurs in therapy-resistant ER+ breast cancers patients) promoted the acquisition of a stem-cell-like phenotype and the upregulation of the Notch4 signaling pathway. Notably, the targeting of the Notch transduction significantly counteracted the mammosphere-forming efficiency and the migratory capabilities of these mutation-presenting breast cancer cells. Mechanistically, the stem features and Notch4 activation in cells bearing this mutation are driven by the phosphorylation of ERα at Ser118, as its selective inhibition counteracted both stemness potential and Notch signaling activation [146]. Collectively, these studies indicate Notch4 upregulation among the mechanisms promoting the resistance to the hormonal therapy in ER+ breast tumors and suggest its targeting as a potential strategy to overcome the relapsed disease.

Notch signaling is also implicated in the maintenance of leukemia stem cells (LSC) or leukemia-initiating cells (LIC) in T-ALL and in AML, which has been confirmed through effective reduction of the LSC pool in vivo, in vitro, and in patient-derived samples by pharmacological inhibition of Notch [147,148]. However, Notch signaling may suppress the activity of LIC in AML, thus acting as an oncosuppressor [149]. Supporting this, ligand-induced activation of any of Notch receptors arrested the growth of AML cell lines and led to caspase-dependent apoptosis and/or differentiation. Indeed, Notch may fulfill its tumor-suppressive function by promoting tumor cell differentiation [150]. This role of Notch signaling is relatively well-described for keratinocyte-derived tumors (reviewed in [72]). In particular, p63 is crucial for epithelial homeostasis, and its isoform ΔNp63 is an important oncogene in SCC. Notch has been found to downregulate ΔNp63, and the Notch/IRF6 axis may be responsible for this inhibition [151,152]. Additionally, in human papillomavirus (HPV)-positive cervical cancer cell lines, the overexpression of Notch-activated domain-inhibited HPV oncoproteins E6 and E7, leading to growth arrest and apoptosis through reactivation of p53 [153]. However, it is worth mentioning that hyperactivation of Notch1 has been indicated as oncogenic in some subsets of cervical cancer, suggesting a contrasting role of Notch in this context [154,155].

In lung homeostasis, Notch blocked the neuroendocrine differentiation through inhibition of ASCL1 [156]. Accordingly, the reactivation of Notch1 suppressed the growth of neuroendocrine lung tumors and small-cell lung cancer (SCLC) by ASCL1 downregulation in vivo and in vitro [157]. Moreover, the oncosuppressive effect of Notch-mediated repression of ASCL1 has been confirmed for other neuroendocrine tumors (NETs) such as medullary thyroid cancer and gastrointestinal carcinoid tumor, thus providing a molecular mechanism for Notch signaling activation as a potential therapeutic target [158,159]. Conversely, high levels of Notch1 underlaid SCLC chemoresistance to doxorubicin that was resolved by *Notch1* knockdown, which once again points out the controversial role of Notch signaling in tumor onset and progression [160]. All these findings suggest that targeting Notch signaling, alone or in combination with other agents, represents a promising therapeutic strategy in various cancers such as ALL [161], breast cancer [162], ovarian cancer [163], NSCLC [164], SCLC [165], colon cancer, and other gastrointestinal tumors [166] and several brain tumors [167], which, however, should be considered carefully due to the tricky balance between oncogenic and oncosuppressive effects of Notch paralogues in the context of a single cancer [168].

## 4. Notch-Targeting Approaches in Preclinical and Clinical Studies

Over the last twenty years, the knowledge on Notch biology and function has grown exponentially. It has stimulated the design of numerous seminatural and synthetic Notch modulators acting on different levels of this pathway. The status of their development is described below.

### 4.1. Gamma-Secretase Inhibitors

Gamma-secretase complex (GS) is an intramembrane cleaving protease containing two stable subunits (nicastrin and presenilin enhancer 2) and two variable subunits (presenilin (PSEN)1/PSEN2 and APH-1A/APH-1B). Different combinations of subunits generate four sub-complexes [169]. GS cleavage can be inhibited by several small molecules called γ-secretase inhibitors (GSIs), most of which target PSEN1 and PSEN2, the proteins of the catalytic core, and to a lesser extent, other subunits of the GS complex [170]. As mentioned in the previous paragraph, this complex is necessary for the third cleavage of all four Notch receptors and its consequent translocation into the nucleus. Reasonably, GSIs are the best-studied Notch signaling modulators, with pan-Notch inhibitory activity preventing Notch signaling activation [171].

GSIs have been proposed as anticancer treatment in tumors with the well-proven oncogenic role of Notch. For example, two different GSIs, MRK-003 and GSI-1, inhibited cell proliferation and induced apoptosis through Notch3 inhibition in Notch3-overexpressing lung and ovarian cancer cell lines, respectively [172,173]. On the contrary, another study demonstrated that the GSI Compound E did not affect ovarian tumor growth in vitro or in vivo but caused angiogenic alterations in an ovarian cancer murine model by reducing microvessel density, thus acting as anti-angiogenetic therapy [174]. Moreover, GSI induced G2/M cell cycle arrest and apoptosis in breast cancer cell lines [175] and breast cancer patient-derived xenografts (PDX) characterized by PEST domain mutations of *Notch*, which were more sensitive to GSI PF-03084014 (also known as nirogacestat) [81]. In addition, another GSI, RO4929097, inhibited tumorsphere formation of a specific population of breast tumor-initiating stem cells (T-ISCs), CD44+ CD24low+, which expressed activated N1IC. Of note, limiting GSI efficacy, the N1IC-negative population of T-ISCs CD44+ CD24- were resistant to GSI [176]. Consistently, tumor explants derived from colon cancers sensitive to GSI PF-03084014 showed higher levels of Notch1 and Wnt/β-catenin signaling components compared with the resistant samples and GSI treatment, affecting the activation of both pathways [177]. These studies suggest that, besides Notch, Wnt activation may also represent a potential biomarker predicting GSI efficacy. Sustaining this hypothesis, it has been demonstrated that limited pro-apoptotic effects of DAPT treatment in Notch1-expressing gastric cancer cells were at least partially due to ERK1-2-dependent upregulation of Wnt-β-catenin signaling as combined inhibition of Notch and ERK1-2 pathway prevented β-catenin induction and enhanced the efficacy of single DAPT treatment [178]. Similarly, in glioblastoma multiforme (GBM) cells, MRK-003 suppressed Notch signaling and activated Wnt and Hedgehog pathways, partially explaining resistance to long-term treatment, whereas a combination of Notch and Hedgehog inhibitors allowed bypassing GSI resistance [179]. In GBM, *PTEN* mutations have also been linked to the mechanisms mediating GSI resistance. Indeed, it has been revealed that, even if GBM CSC are generally susceptible to GSI, stem-like populations with low or absent PTEN expression were insensitive to GSI due to the upregulation of PI3K/AKT pathway. Therefore, targeting PI3K/AKT together with GS might be of a great advantage, as it was for the combination of PI3K inhibitor BKM120 and GSI RO4929097 [180,181]. *PTEN* mutation status predicted the response to GSI also in melanoma and T-ALL [104,182]. In the case of melanoma, RO4929097 induced senescence or apoptosis only in *PTEN* wild-type (WT) cell lines, whereas *PTEN* null or mutated cells were GSI-resistant [182]. In T-ALL with gain-of-function mutations of *Notch1*, GSI treatment led to rapid clearance of Notch1 signaling and resulted in G1 cell cycle arrest or apoptosis, whereas GSI resistance was related to *PTEN* and *FBXW7* mutations that sustained leukemic cell proliferation despite Notch signaling inhibition [75,85,86,104,183,184]. Overall, the results of preclinical studies have shown that GSI might be a promising treatment for several cancers with Notch hyperactivation; however, GSI alone could upregulate other survival pathways resulting in partial or complete insensitivity to GS inhibition, therefore making it highly promising to combine GSI with other drugs. Additionally, a synergic effect between GSI and chemotherapy or radiotherapy might become a rational strategy to counteract resistance mechanisms due to a well-known activation of Notch signaling in response to conventional treatment [185,186,187,188]. In colon cancer, oxaliplatin, 5-fluorouracil (5-FU), or SN-38 induced expression of GS subunits that resulted in increased activation of the Notch1/HES1 axis associated with chemoresistance, whereas addition of GSI134 to conventional drugs drastically reduced cell viability through downregulation of downstream survival pathways such as PI3K/AKT. [187]. In breast cancer, doxorubicin upregulated the Notch1/multidrug-resistance-associated protein-1 (MRP1) axis, thus reducing the effective intracellular concentration of the cytotoxic agent. Reasonably, co-treatment with DAPT increased cellular retention of anthracycline and enhanced cell death without affecting non-tumoral cells [185]. Notch1-mediated enrichment of drug efflux transporters and CSC population after chemotherapy was also described for NSCLC; however, DAPT pretreatment negatively influenced cisplatin-induced CD133^+^ cell selection and increased the sensitivity to doxorubicin and paclitaxel [186]. Confirming the utility of GSI in counteracting Notch-induced CSC enrichment after eradication of rapidly dividing cells, DAPT improved the response of GBM explants to radiotherapy by targeting tumoral endothelium, whereas the addition of RO4929097 to the standard dual-protocol of care (radiation + temozolomide) reduced GBM tumor growth and prolonged mice survival compared with the conventional treatment [125,189]. In T-ALL, Notch1 target gene HES1 mediated resistance to dexamethasone through inhibition of glucocorticoid receptor auto-upregulation, a positive feedback loop necessary for glucocorticoid-induced apoptosis. Consequently, the addition of GSI reverted this mechanism and enhanced dexamethasone cytotoxicity. Moreover, combining GSI and glucocorticoids had a beneficial effect on GSI-induced gut toxicity by preventing GSI-inducing goblet cell metaplasia [190,191].

Unfortunately, the clinical use of GSI is limited by their adverse effects such as diarrhea, nausea, vomiting, skin rash, and thrombocytopenia, often related to on-target inhibition of Notch in normal tissues, especially in the gut, where GSI causes goblet cell metaplasia redirecting gastrointestinal progenitor differentiation from absorptive to secretory and enteroendocrine phenotypes [192,193]. Furthermore, GSIs affect the cleavage of other γ-secretase substrates such as amyloid precursor proteins (APP), all Notch ligands, N-cadherin and E-Cadherin, syndecan-3, CD44, and ERBB4, widening the spectrum of both antitumoral and toxic effects of GSI [170,194]. Notably, PF-03084014 could influence initially NF-κB phosphorylation and caspase 3 and PARP (poly (ADP-ribose) polymerase) cleavage with a subsequent decrease in Notch activation, confirming the existence of Notch-independent off-target substrates of GSI [177]. Therefore, the application of GSIs is restricted by their on- and off-target toxicity that makes it highly relevant to develop GSIs with selective substrate inhibition capacity. Indeed, GSIs may be pharmacologically and functionally different in their effects on Notch receptors or APP (Table S1), since BMS-906024 equally affected all Notch receptors and APP, whereas PF-3084014 inhibited Notch2 to a greater extent than other Notch receptors; moreover, at low concentrations it increased Notch3 cleavage [194]. Highlighting the importance of tissue distribution of differently composed γ-secretase complexes, GSI MRK-560 had higher affinity to PSEN1 than to PSEN2-containing GS complex providing a strategy to selectively inhibit GS of T-ALL cells, expressing only PSEN1 without affecting the physiological function of Notch in the gastrointestinal tract, where PSEN2 compensated the lacking activity of PSEN1 (Table S1) [169]. GSI MK-0752, LY3039478, RO4929097, and PF-03084014 were relatively well-tolerated at Notch-inhibitory doses and showed modest antitumor activity at early phase clinical trials (CT), with frequent toxic reactions such as diarrhea, nausea, fatigue, hypophosphatemia, vomiting, rash, and decreased appetite [195,196,197,198]. In the case of high-grade glioma, monotherapy with MK-0752 allowed to achieve complete response (CR) in one patient and stable disease (SD) for more than 4 months in 10 out of 21 recruited individuals [195]. LY3039478, tested in heavily pretreated patients with advanced or metastatic cancer, caused partial response (PR) in one case of breast cancer (Estrogen/Progesterone receptor+, HER2-, *FBXW7* mutated) and SD in around one-third of patients receiving different GSI dose regimens. Additionally, clinically relevant tumor necrosis or shrinkage or metabolic responses were observed in individuals with breast cancer, leiomyosarcoma, and ACC [196]. Another phase I study showed limited clinical activity without confirmed CR or PR of the same GSI in heavily pretreated patients with ACC, and 68% of patients had SD for ≥6 months [199]. Considering the appealing prospective of GSI use for the treatment of *Notch*-mutated ACC, BMS-906024 (re-registered as AL101) was granted Orphan Drug Designation by the Food and Drug Administration (FDA) [200]. Similarly, RO4929097 showed modest activity in patients with advanced solid tumors with achievement of SD in 25% of treated patients, and single cases of PR in an individual with colon cancer and nearly complete FDG-PET response in a patient with melanoma [198]. Disappointingly, several phase II CT in patients with advanced, metastatic, or resistant solid cancers demonstrated insufficient therapeutic activity of RO4929097 as a single agent and resulted in the termination of its clinical development [200]. PF-03084014 had acceptable tolerability and allowed to achieve CR in one case of advanced thyroid cancer and PR in several patients with desmoid tumors [197]. Further studies of PF-03084014 demonstrated PR or SD in all evaluable patients with desmoid tumors, and consequently, the agent has obtained breakthrough designation status for the treatment of adult patients with progressive, unresectable, recurrent, or refractory therapy desmoid tumors or deep fibromatosis [200,201]. Moreover, the same GSI has been evaluated in a phase I trial of a small group of adult patients with T-cell lymphoblastic lymphoma or T-ALL after the failure of prior therapy, and treatment with PF-03084014 was associated with CR in one heavily pretreated T-ALL patient with Notch-activating mutation [201].

Overall, these CT have demonstrated that monotherapy with GSI has limited antitumor activity in advanced tumors, with the exception of PF-03084014 for the treatment of desmoid tumors. The complete list of CT involving GSI as a monotherapy is shown in Table S2. Currently, the benefits of combining GSI with other antitumor drugs are being evaluated in several clinical studies [200].

### 4.2. Notch-Targeting Antibodies

Modest efficacy and disputable selectivity of GSI have encouraged the search for more selective Notch signaling inhibitors and have led to the development of antibodies against Notch receptors and ligands specifically preventing Notch signaling activation (summarized in Table S1) [202]. Notch-targeting antibodies can be subdivided into antibodies counteracting conformational change after the linkage with ligands and antibodies directly preventing the ligand binding. The first group includes antibodies against the NRR domain of Notch receptors that block Notch activation, preventing the conformational change of the NRR region and hindering ADAM10 proteolytic cleavage. Anti-NRR antibodies directly inhibiting cancer cell growth and disrupting tumor angiogenesis are quite strong drug candidates for targeted therapy [203]. Biological effects of anti-NRR antibodies have been vigorously studied in cells harboring class I *Notch1* mutations (HD mutation with in-frame deletions or insertions in extracellular heterodimerization domain) [204]. Tarextumab (OMP-59R5), a monoclonal antibody (mAb) against Notch2 and Notch3, has shown promising antitumor activity in several in vitro and in vivo models of SCLC, breast, ovarian, and pancreatic cancer that correlated with downregulation of Notch target genes [205]. Like GSI, tarextumab exhibited gastrointestinal adverse effects due to Notch inhibition in intestinal crypt progenitor cells. However, its undesirable action could be mitigated with intermittent schedule or glucocorticoids [206,207]. Consequently, this mAb was evaluated in several CTs in combination with chemotherapeutic drugs, which did not bring any relevant benefit in patients with advanced SCLC or metastatic pancreatic cancer (NCT01859741) [208]. Brontictuzumab (OMP-52M51), a mAb directed against NRR of Notch, was effective in *Notch1*-mutated T-ALL, CLL, mantle cell lymphoma, and ACC cell and murine models [209,210,211,212]. This agent has been studied in several phase I CTs of relapsed or refractory lymphoid malignancies, solid tumors, and previously treated metastatic colon cancers (NCT01778439, NCT01703572, NCT03031691), with some efficacy signals in patients with ACC associated with *Notch1*-activating mutations [212,213]. It is worth mentioning that not only blocking but also activating antibodies against NRR have been developed, however, just for Notch3 by now [214].

It is noteworthy that Notch-targeting antibodies may be used to develop immunoconjugates, selectively delivering cytotoxic agents inside Notch-expressing cancer cells and minimizing the exposure of normal tissues. A novel Notch3-targeting antibody conjugated to a cytostatic agent, auristatin, demonstrated promising antitumor activity in preclinical models of breast cancer, NSCLC, and ovarian cancer as well as a manageable safety profile and preliminary signs of antitumor activity (PR and SD in more than 50% of enrolled patients) in advanced solid tumors in a phase 1 CT (NCT02129205) [215,216].

The second group of antibodies is directed against ligands or EGF-like repeats and prevents the ligand/receptor interaction, thus being particularly useful when the oncogenic role of Notch depends on aberrant ligand-dependent activation. Illustrating this, a mAb targeting anti-EGF-like repeats of Notch1 (602.101), sensitive to Ca^2+^-induced conformational changes of the receptor, prevented the binding of Jagged1, DLL1, and DLL4 and had selective activity against breast cancer CSC, enhancing apoptosis and increasing chemo- and radio-sensitivity of resistant cells [217]. Jagged1 is often upregulated in tumor cells and plays an important role in neoplastic vascularization, maintenance of immunosuppressive microenvironment and CSC, thus making it a highly appealing target for designing selective mAb (reviewed in [218]). Antibodies against Jagged1 targeted both tumor- and stroma-expressed Jagged1, blocked Notch/Jagged signalization between smooth muscular and endothelial cells, affected CSC, and effectively reduced metastatic brain lesions in in vitro and in vivo models of breast cancer. In human breast cancer, elevated expression of Jagged1 (and Notch1) is associated with osteolytic bone metastasis and poor prognosis [219]. In osteoblasts, Notch activation by tumor-derived Jagged1 increased Interleukin-6, which supported the survival of metastatic breast cancer cells. Meanwhile, Jagged1 stimulated osteoclastogenesis and bone degradation, releasing TGFβ, a potent inducer of Jagged1. A fully human monoclonal antibody against Jagged1 (clone 15D11) with minimal toxicity has been developed. Besides its inhibitory effect on bone metastasis of Jagged1-expressing tumor cells, the group of Kang demonstrated that this anti-Jagged1 sensitized bone metastasis to chemotherapy [16,220]. Indeed, targeting Jagged1 may circumvent drug-associated toxicity and prevent bone metastasis. Additionally, the effects of anti-Jagged1 on tumor vasculature might provide a promising curative alternative for patients refractory to VEGF inhibitor bevacizumab [221]. DLL4 represents an attractive target for cancer therapy since the blockade of DLL4/Notch signaling has been shown to cause non-productive tumor angiogenesis, to reduce the growth of VEGF-sensitive and resistant tumors, and to affect the CSC pool [221,222,223]. However, chronic administration of anti-DLL4 in preclinical studies revealed their potentially significant toxicity due to abnormal activation of endothelial cells, possible induction of vascular neoplasms, and associated damage of multiple organs including liver, thymus, heart, and lung [224]. Multiple early-stage CTs of the first-in-class anti-DLL4 antibody demcizumab (OMP-21M18) in combination with other chemotherapeutic drugs have been registered: demcizumab + gemcitabine + nab-paclitaxel in pancreatic cancer (NCT01189929), demcizumab + FOLFIRI in colorectal cancer (NCT01189942), demcizumab + carboplatin + pemetrexed in NSCLC (NCT01189968), demcizumab + paclitaxel in patients with platinum-resistant ovarian cancer (NCT01952249). Notably, the antitumor activity of demcizumab looked quite promising in the case of advanced NSCLC and ovarian cancer despite some clinically relevant cardiotoxicity manifested as hypertension and elevated risk of congestive heart failure [225,226,227]. Another blocking DLL4 antibody, enoticumab, (REGN1035) showed potent anti-tumor activity against renal cell carcinoma and ovarian cancer [228,229]. It was further evaluated in a phase I trial in patients with advanced solid tumors, where it was generally well-tolerated with some evidence of cardiotoxicity and registered cases of PR in NSCLC and ovarian cancer and SD in around one-third of treated subjects [230]. One more anti-DLL4 antibody, MEDI0639, induced non-productive angiogenesis in vivo; however, its clinical tolerability and efficiency were not so encouraging (NCT01577745) [231]. Since DLL4/Notch signaling is a negative regulator of the VEGF/VEGFR-2 axis, combined inhibition of these two pathways becomes a double-edged sword acting against both the quality and the number of tumoral vessels and provides significant antitumoral benefits [229,232]. Indeed, simultaneous blockade of DLL4 and VEGF by bispecific antibodies HD105 and HB32 showed potent anti-tumor and anti-angiogenic activity in vivo and in vitro [233,234]. A bispecific anti-DLL4/anti-VEGF antibody, navicixizumab (OMP-305B83), was evaluated in several early-phase CTs of metastatic colorectal cancer (NCT03035253), platinum-resistant ovarian cancer in combination with paclitaxel (NCT03030287), and advanced solid tumors (NCT02298387), where it showed preliminary signs of antitumor activity often associated with cardiovascular adverse events such as arterial and pulmonary hypertension, being most promising in ovarian cancer [235]. Another bispecific anti-DLL4/VEGF antibody, ABT-165, outperformed tumor response with single anti-VEGF treatment in preclinical models of glioma, breast cancer, and colon cancer alone and in combination with standard-of-care chemotherapy drugs [236]. It is being currently evaluated in two CT: a phase II RCT of FOLFIRI+ABT-165/bevacizumab in pretreated patients with metastatic colon cancer (NCT03368859) and in a phase I trial in advanced solid tumors as monotherapy or in combination with paclitaxel or 5-FU (NCT01946074). DLL3 is overexpressed on the surface of SCLC and other NETs, becoming an appealing target for designing mAbs [237]. The bispecific T-cell engager (BiTE^®^) AMG757 interacts with DLL3 expressed by SCLC cells and CD3ε of T cells, redirecting them to initiate the cytotoxic response against malignant clones [237]. It was highly potent against SCLC in vitro and in vivo and is currently under evaluation in a phase 1 CT (NCT03319940) [238]. The antibody-drug conjugate Rovalpituzumab tesirine (Rova-T, AbbVie), composed of a human DLL3-specific mAb, the DNA-crosslinking agent pyrrolobenzodiazepine, and a protease-cleavable linker, effectively eradicated neuroendocrine CSCs in vivo and abolished chemotherapy-resistant SCLC and large cell neuroendocrine carcinoma (LCNEC) xenograft growth [239]. It showed modest efficiency (objective response (OR) in 12–18% of patients and acceptable tolerability) as a single agent for SCLC and LCNEC treatment in several early-phase CTs (NTC01901653, NCT02674568, NCT03086239). However, it provided lower overall survival (OS) compared with topotecan (NCT03061812) and gave no survival benefit as maintenance therapy in platinum-pretreated patients (NCT03033511), whereas its combination with nivolumab and ipilimumab was not well tolerated (NCT03026166) [240,241,242,243,244,245]. Another phase I/II CT evaluated the tolerability of agents in delta-like protein 3-expressing advanced solid tumors (melanoma, medullary thyroid cancer, glioblastoma, various NET) and revealed some signs of context-dependent benefit, expressed as 4.3–22.6% objective response rate (ORR) (NCT02709889). Due to the suboptimal results of clinical trials, its development has been terminated [246]. The third DLL3-targeting approach (NCT03392064) uses CAR-T cells modified to recognize DLL3-positive cells and opens the road for the development of personalized therapies in NETs overexpressing this Notch ligand [247]. The complete list of CTs involving mAbs as a monotherapy is shown in Table S3. Of note, although growing evidence indicated the selective targeting of Notch4 of therapeutic relevance in certain tumors, including the ER+ metastatic hormone-refractory breast cancer, no Notch4-blocking antibodies are under clinical investigation.

### 4.3. Targeting Notch Transcriptional Complex

The search for more specific Notch inhibitors has led to the development of strategies to target the pathway downstream of the GS-mediated activation of Notch receptors, with particular attention to the key components of the Notch transcription complex (MAML and RBP-jκ). Bradner and colleagues designed a synthetic and stabilized a-helical peptide named SAHM1 that competitively inhibited MAML1 binding, thus preventing the assembly of the Notch active transcriptional complex. The SAHM1-dependent inhibition of Notch transcriptional program suppressed the proliferation of Notch-dependent human T-ALL cells sensitive to GSI as well as the cancer progression in murine models of T-ALL without associated gastrointestinal toxicity [248]. Subsequently, a novel small molecule was developed named IMR-1 (inhibitor of Mastermind recruitment-1), which selectively inhibits NTC by preventing the recruitment of MAML1 to the complex [249]. IMR-1 decreased colony formation of Notch-dependent cancer cell lines sensitive to DAPT and blocked tumor growth in PDX mouse models by decreasing the expression of Notch target genes, without any observable adverse effects [249]. In addition to MAML specific inhibitors, two novel small molecules that selectively target RBP-jκ-NIC binding have been developed: RBP-jκ inhibitor-1 (RIN1) and CB-103 [250,251]. In particular, RIN1 inhibited the transcriptional activation of Notch downstream target genes and suppressed the Notch-dependent growth of three hematological cancer cell lines by interfering with the functional association of RBP-jκ with NIC and SHARP. However, this chemical inhibitor is yet to be tested in vivo for intestinal toxicity [250]. On the other hand, CB-103 functions as a pan-Notch inhibitor, similar to GSI, but its advantage is that it is active against tumor cells carrying any type of Notch mutations. Indeed, CB-103 counteracted the growth of Notch-dependent human T-ALL and TNBC cells, both in vitro and in xenotransplanted mice, including those carrying rearrangement of *Notch* genes that drive the resistance to GSI treatment [252]. Furthermore, differently from GSI, CB-103 did not cause the anticipated goblet cell metaplasia in mice [251]. For this reason, CB-103 is currently in phase I/II clinical trials in adult patients with hematological malignancies and advanced or metastatic solid tumors (NCT03422679).

Of note, recently, a novel orally available, potent, and selective Notch1 inhibitor NADI-351 was developed. Indeed, NADI-351 disrupted Notch1 NTC without any effects on Notch2-4 transcriptional complex [253]. Interestingly, NADI-351 suppressed tumor growth in Notch-dependent cell lines and PDX models without inducing goblet cell metaplasia or other collateral effects that could be caused by pan-Notch inhibition. Moreover, it specifically targeted the Notch1-positive CSC subpopulations [253].

Another way to target Notch signaling at the level of Notch transcriptional activity is the specific inhibition of its target genes. Accordingly, a small molecule, JI051, has been discovered, which induced cell-cycle arrest in HEK293 cell lines by targeting the Notch downstream target gene HES1. Furthermore, a JI051 derivative, JI130, reduced the growth of pancreatic cancer cell lines in vivo and in vitro [254].

Overall, these studies provide the evidence that targeting of the Notch transcriptional complex (Table S1) could be an effective anti-cancer strategy in Notch-driven tumors without the limiting side-effects associated with other Notch inhibitors.

### 4.4. Targeting Notch Receptor Maturation

Targeting Notch receptor maturation may become a promising therapeutic approach to block Notch signaling in contexts where it functions in a ligand-independent manner due to the activating *Notch* or inactivating *FBXW7* mutations (T-ALL, CLL, and mantle cell lymphoma) [255]. Notch maturation occurs in endoplasmatic reticulum (ER), where sarco/endoplasmic reticulum Ca^2+^-ATPase (SERCA) uses ATP to pump Ca^2+^ from the cytoplasm to internal compartments. Ca^2+^ is important for the interaction between Notch and its ligands through its Ca^2+^-binding EGF-like and Lin12/Notch repeats; therefore, the lack of intraendoplasmatic Ca^2+^ inhibits Notch processing [256]. EGF-like repeats may be modified by O-fucosyltransferase1 (Pofut1) responsible for O-fucosylation of these motifs. Mammalian Pofut1 is not essential for Notch receptors such as the homologous Ofut1 in Drosophila, but its lack prevents Notch activation, probably because of improper folding of the receptor precursor [257]. The importance of SERCA makes it a highly attractive subject for Notch inhibition. Thapsigargin (TG) is a natural SERCA inhibitor that causes accumulation of defective Notch1 precursors in the ER-Golgi complex and induces ER stress [258]. Several mutations can influence biological response to TG since cells harboring class I HD *Notch1* mutations and mutations in polypeptides regulating ER stress response were more sensitive to this molecule, whereas Notch1 precursors with mutations in EGF-like and LNR domains were refractory to TG [258,259]. Indeed, the combination of TG and mAb against the NRR of Notch1 (MAb604.107) was particularly effective against T-ALL cell lines with mutated *Notch1*, however, did not affect WT Notch [260]. Even if it might be considered a promising candidate for targeted therapy, TG is poorly tolerated; however, conjugating folate to an alcohol derivative of TG has led to the development of the compound JQ-FT, which is selectively recognizable by T-ALL due to the high expression of folate receptor on the surface of leukemic cells [255]. Likewise, casearin J possessed the same effect only in *Notch1*-HD mutated T-ALL cell lines, downregulated Myc and HES1 expression, and induced oxidative stress and apoptosis. In addition, this compound was shown to synergize with the NF-κB inhibitor, which is of a possible therapeutic importance [261].

Similar alterations in Ca^2+^ current were caused by bepridil, a non-selective ion channel blocker, used for the treatment of angina pectoris and arrhythmia [262]. Bepridil induces Ca^2+^ release from multiple stores (mitochondria, ER, and other non-identified locations) in a phospholipase C-independent manner, increasing cytosol Ca^2+^ levels [263]. Like SERCA inhibitors, bepridil blocked Notch1 maturation, reduced Notch1 activation, did not influence Notch2 expression, and, consistent with its mechanism of action, reduced ER Ca^2+^ pool in CLL [264]. Additionally, bepridil enhanced the cytotoxic effects of ibrutinib, a BTK inhibitor used for CLL treatment; however, the interaction between BCR and Notch1 pathways in CLL requires further studies to identify the subsets of patients who could benefit from this therapy [265,266].

Consistent with the above-mentioned findings, affecting Notch precursor maturation appears to be an appealing strategy for developing new therapies for cancers where *Notch* mutations lead to the ligand-independent activation of Notch signaling. The complete list of Notch maturation-affecting approaches is summarized in Table S1.

### 4.5. Affecting Notch-Signaling-Related Epigenetic Events

Epigenetics modifications lead to change in gene expression without affecting DNA sequence. DNA methylation, histone modifications, and miR regulation are the key epigenetic mechanisms regulating the Notch pathway’s components [267]. Numerous studies suggest epigenetic machinery as a new target in cancer therapy. Therefore, targeting epigenetic mechanisms may represent potential strategies to modulate Notch signaling in cancer (summarized in Table S1).

#### 4.5.1. DNA Methylation Pattern of Notch-Related Genes in Cancer

DNA methylation is one of the prevalent epigenetic modifications. Hyper-methylation reduces gene expression, whereas hypo-methylation upregulates it. Both aberrant hypo-methylation and hyper-methylation have been described in different genes of the Notch pathway in several types of cancer [268].

In diffuse gastric cancer (DGC), Notch signaling inactivation was associated with hyper-methylation of the *DLL1* ligand gene. Consequently, the treatment of DCG cell lines with DNA methyltransferase inhibitor 5-aza-2′deoxycitidine (DAC) reactivated Notch signaling by upregulating DLL1, which in turn repressed the expression of the oncogene HATH1 [269]. Conversely, in clear cell renal cellular carcinoma and in breast cancer, DNA hypo-methylation of *JAG1* partially mediated oncogenic hyper-activation of Notch signaling, favoring tumor progression [270,271].

Besides ligands, receptors, target genes, and other modulators of the Notch pathway could also be regulated by aberrant methylation. Silenced *Notch3* and *HES5* are hyper-methylated in B-ALL primary samples and cell lines, whereas in T-ALL, high expression of these genes combined with unmethylated or weakly methylated status at their regulatory regions [272]. Accordingly, DAC treatment restored the expression of Notch signaling components in cell lines in which they were silenced [272]. On the other hand, in vincristine-resistant gastric cancer cell line SGC7901/VCR, treatment with lenalidomide enhanced DNMT3A expression, increased DNA methylation and downregulated *Notch2* expression, providing a limited cytotoxic effect and suggesting this agent as a therapeutic approach in drug-resistant gastric cancer [273]. Similarly, the naturally occurring substance resveratrol inhibited *MAML2* gene expression by increasing methylation in its enhancer region and consequently suppressed Notch signaling, invasive capacity, and proliferation in breast cancer cell lines [274].

Taken together, these studies demonstrated that DNA methylation-affecting agents might be useful modulators of Notch signaling in cancer. Some molecules such as DAC or 5-azacytidine have been approved for the treatment of myelodysplastic syndrome and AML; however, their activity in solid tumors is considered limited because of high toxicity, whereas other DNA-hypomethylating agents such as guadecitabine are being investigated in ongoing CT [275].

#### 4.5.2. Histone Modifications Drive Aberrant Notch Signaling Activity in Cancer

In addition to DNA methylation, gene expression can be regulated through post-translational modification of specific amino acid residues of histone proteins [276]. Several histone modifiers, such as histone deacetylases (HDAC), histone acetyltransferases (HAT), lysine demethylases (KDM), and histone methyltransferases (HMT), play a pivotal role in regulating Notch signaling by facilitating or repressing the transcriptional availability of Notch target genes (reviewed in [267]).

HDAC-dependent corepressors complexes, such as CIR/SAP30/HDAC2, which directly binds RBP-jκ, and SMRT/mSin3A/HDACs, NCor/mSin3A/HDACs, and CtBP/SIRT1, which bind SHARP, an RBP-jκ interacting protein, are recruited by RBP-jκ in the absence of NIC [10,277]. In line with the key role of HDACs in regulating Notch signaling, a common antiepileptic drug, valproic acid (VPA), possessing HDAC4 inhibitory activity, increased the level of acetylated histone (H) 4 in NET, reactivating Notch signaling and suppressing cell proliferation [278,279]. Likewise, a pilot phase II CT in low-grade NET showed that VPA treatment upregulated Notch1 expression and resulted in a better clinical response. Unfortunately, the small number of patients in this study complicated the evaluation of a real clinical benefit of VPA treatment and its role in Notch signaling activation, and larger CT could be necessary to confirm this finding [280]. Additional HDACs inhibitors are used or being evaluated in CT for cancer treatment (reviewed in [281]). These findings suggest the use of HDAC inhibitors reactivating Notch signaling in tumors in which Notch acts as an oncosuppressor. On the other hand, when Notch acts as an oncogene, its upregulation could become a resistance mechanism to HDAC inhibitors. Indeed, the failure of the clinical trials in ovarian cancer with the HDAC inhibitor vorinostat could be due to Notch signaling activation [282].

In addition to HDAC, HMTs and KDMs contribute to the regulation of Notch signaling. SHARP, an RBP-jκ interacting factor, was able to switch the binding to NCor/HDAC complex with KMT2A (lysine methyltransferase 2A), promoting trimethylation of H3K4 and providing permissive chromatin state at Notch target genes before Notch activation [283]. On the other hand, the H3K4-demethylases KDM5A and LSD1 (lysine demethylase 1) are essential components of the RBP-jκ repressor complex and activity maintains a low level of H3K4me3 [284,285,286]. In line with this, KDM5A promoted SCLC proliferation by repressing *Notch2* and Notch signaling and sustaining expression of a neuroendocrine TF ASCL1, while *KDM5A* knockout restored expression of *Notch2* and Notch target genes, reverting ASCL1 expression and blocking tumor growth and thus suggesting KDM5A as a possible therapeutic target in SCLC [287]. Likewise, in the same cancer, an LSD1 inhibitor, ORY1001, favored Notch1 expression and suppressed tumor growth in vitro and in vivo through Notch-dependent ASCL1 repression [157]. Elevated *Notch1* expression was associated with increased levels of acetylated H3K27 in its promoter region, probably due to the presence of HDAC1 and HDAC2 in LSD1 complex [157,286]. On the contrary, in T-ALL, LSD1 is a component of NTC that is recruited to sustain Notch transcriptional activity by permitting low levels of dimethylated H3K9; therefore, depletion of this demethylase induced cell cycle arrest in Notch-dependent T-ALL cell lines [288]. These studies suggest LSD1 as a bivalent target-modulating Notch signaling, both as oncogene and as oncosuppressor. Indeed, the LSD1 inhibitor ORY-1001 is under clinical investigation in ALL and AML, in which inhibition of LSD1 was pro-differentiative, however not sufficient to induce cell death as monotherapy [275].

In order to activate the transcription of Notch target genes, Notch-RBP-jκ-MAM ternary complex recruits histone acetyltransferases (HAT) such as PCAF, GCN5, and p300 [277]. p300 is required for acetylation of H3K27, a histone modification critical for the regulation of Notch signaling [289,290]. Indeed, in T-ALL, a subset of NIC-binding sites responsive to Notch modulation was identified through switching between Notch on/off states, and the dynamic association of NIC with these sites correlated with a dramatic change in H3K27ac levels at promoters of Notch target genes [290,291]. Both H3K27 acetylation and methylation are crucial for regulating Notch signaling. NTC requires histone lysine demethylase PHF8 to maintain low levels of demethylated H3K27 and to permit Notch transcriptional activity [288]. Moreover, during the physiological development of T-lymphocytes, Ikaros shuts down Notch signaling in DN4 (double negative 4) precursors by recruiting PRC2 (Polycomb repressive complex 2) to Notch target genes, which increased levels of H3K27me3 [292]. The levels of H3K27me3 are controlled by the methyltransferase EZH2 (enhancer of zeste homolog 2), a key component of PRC2 complex, and the demethylases JMJD3 (Jumonji domain-containing protein 3) and UTX (ubiquitously transcribed tetratricopeptide repeat X-linked protein) [293,294]. PCR2 is a known tumor suppressor which antagonizes Notch activity in T-ALL, and loss-off-function mutations of PCR2 complex components were attributed to 25% of T-ALL patients [295]. On the other hand, in an N1IC-induced-T-ALL mouse model, Notch1 antagonized EZH2 and cooperated with JMJD3 to support the demethylated state of H3K27 on Notch target gene promoters and favor their transcription [295,296]. Furthermore, treatment with GSK-J4, a JMJD3 inhibitor, arrested tumor growth in primary human T-ALL cell lines and xenograft models [296]. In line with the importance of H3K27ac and H3K27me3 in modulating Notch signaling in T-ALL, we have recently demonstrated that both demethylation and acetylation of H3K27 enhanced the expression of *Notch3* under the control of Notch1 or Notch3 in Notch-dependent T-ALL [297]. Consistently, inhibition of JMJD3 and p300 in different human T-ALL cell lines induced apoptosis and growth arrest, partially by silencing Notch signaling, suggesting also p300 as a possible therapeutic target for Notch-driven T-ALL [297].

Despite the key role of EZH2 in repressing Notch signaling in T-ALL, a positive correlation between EZH2 and Notch was found in glioblastoma and breast cancer in which EZH2 directly binds *Notch1* promoter, upregulating Notch1 expression without any change in H3K27me3 levels [298,299]. Furthermore, in sorafenib-resistant HCC cell lines, in which Notch1 and EZH2 enhance self-renewal and tumorigenicity, knockdown or pharmacological inhibition of EZH2 suppressed Notch1 signaling activity through upregulation of Notch1-related microRNAs (miR-21-5p and miR-26a-1-5p) and abrogated CSC stemness, suggesting the EZH2/Notch1 axis as a rational therapeutic target [300].

Confirming the oncogenic Notch/JMJD3 crosstalk, in colorectal cancer, activated Notch recruited JMJD3 to the *EPHB4* gene promoter, enhancing tumor cell growth in vitro and in vivo [301]. Additionally, colon cancer resistance to oxaliplatin could be related to the upregulation of JMJD3 and UTX, decreasing the tri-methylation of H3K27 at *Notch2* gene and permitting its transcription, whereas the addition of GSK-J4 notably potentiated platinum-drug induced apoptosis [302]. Similarly, inhibition of the lysine demethylase 2A (KDM2A), which catalyzes the trimethylation of H3K36, sensitized breast cancer cells to cisplatin and blocked tumorsphere formation by inhibiting Notch [303]. Taken together, these studies demonstrated the appealing prospective of targeting histone modifications modulating Notch expression to overcome tumor resistance to chemotherapy.

#### 4.5.3. Targeting the miRNA–Notch Axis in Cancer

MicroRNAs are a class of non-coding RNAs that regulate gene expression through RNA-induced silencing complex (RISC). Increasing evidence has demonstrated that the interplay between Notch and miRNAs is implicated in cancer initiation/progression, metastasis, and chemoresistance (reviewed in [95]).

Indeed, the tumor-suppressive function of miR-34 was at least partially related to Notch targeting. Moreover, in ovarian cancer, the overexpression of miR-34 mimic downregulated Notch1, and it induced cell death and autophagy, whereas Notch1 transfection reverted its anti-proliferative effects [304]. In colorectal cancer primary samples, miR-34 was weakly expressed and inversely correlated with metastasis, whereas miR-34 overexpression suppressed cell invasiveness and migration by targeting *Notch1* and *JAG1* [305]. Moreover, miR-34 played a critical role in regulating the choice between self-renewal and differentiation of CSC in a Notch-dependent way in colon cancer, breast cancer, and glioblastoma [306,307,308]. In line with this, in breast cancer, Notch1 and miR-34 expressions were inversely correlated, and miR-34 mimic sensitized chemoresistant breast cancer cells to doxorubicin and paclitaxel, thus suggesting a possible advantage of Notch inhibition by replacing miR-34 in combined therapeutic strategies [307,309,310]. In NSCLC, treatment with delta-tocotrienol upregulated miR-34 and suppressed cell proliferation and invasion partially through affecting Notch1 [311]. Furthermore, in cholangiocarcinoma, inhibition of EZH2 or DNA methylation repressed tumor cell growth in vitro and in vivo through the miR-34/*Notch1* axis [312]. In colorectal cancer, DAC treatment enhanced expression of another Notch-targeting microRNA miR-139-5p and sensitized multidrug-resistant CSC to 5-FU, mitomycin C, oxaliplatin, and vincristine via Notch inhibition [313,314]. In ovarian cancer, DAC upregulated miR-199, which suppressed tumor growth and enhanced cytotoxicity of cisplatin in vitro and in vivo by shutting down *JAG1* mRNA and its overexpression, thus suggesting the targeting of chromatin remodelers to indirectly modulate Notch signaling with miRNAs [315]. Similar to miR-199, miR-449 was downregulated in cisplatin-resistant ovarian cancer cells, and its overexpression inhibited Notch and sensitized tumor cells to this platinum drug [316]. In line with the pivotal role of the miR/Notch axis in mediating cisplatin resistance, Ma et al., found that miR-129-5p is downregulated in NSCLC CD133+ stem cells, whereas its exogenous expression inhibited stemness and allowed to overcome drug resistance affecting Notch ligand DLK1 expression [317]. On the other hand, Notch can mediate tumor chemoresistance by regulating several miRNAs in a downstream way.

The Notch-NF-κB axis acted as a co-regulator on promoting transcription of oncogenic miR-223 that sustains proliferation in Notch-dependent T-ALL through the negative regulator of Notch FBXW7 [111]. Similarly, Notch or AKT inhibition reduced levels of miR-223 in NSCLC cell lines resistant to the anti-EGFR agent erlotinib, whereas suppression of miR-223 sensitized cells to this kinase inhibitor by increasing expression of *FBXW7* [318]. Taken together, these studies show that targeting miRs/Notch crosstalk may sensitize cancer cells to chemotherapy, suggesting targeting Notch-related miRNAs in combined treatment strategies.

### 4.6. Targeting Post-Translational Modifications for Notch Signaling Modulation

Targeting histone acetyltransferases or HDAC may regulate Notch signaling not only at the level of transcription and chromatin remodeling but also by affecting post-translational modification (PTM) of Notch pathway’s components. Indeed, p300/CBP-dependent MAML1 acetylation in NTC engaged NACK that, in turn, recruited RNA polymerase II on Notch target genes’ promoters. Reasonably, in esophageal adenocarcinoma, in which Notch and NACK play a critical pro-survival role and CBP is highly expressed, combining GSI with p300/CBP inhibitor C646 decreased Notch transcription activity, reduced tumor growth and triggered apoptosis, demonstrating that affecting Notch pathways at multiple levels may be beneficial for tumor growth suppression [319].

Acetylation/deacetylation status of Notch3 might determine its proteasomal or lysosomal degradation. From one side, p300 acetylated N3IC, favoring its ubiquitination and proteasomal degradation, whereas HDAC1 reverted p300-dependent acetylation stabilizing N3IC. Therefore, a pan-HDAC inhibitor Trichostatin A provided N3IC hyper-acetylation and consequent proteasomal degradation, abolishing T-ALL development and progression in N3IC-transgenic mice [320]. On the other hand, HDAC6-dependent deacetylation of Notch3 was crucial for protecting it against lysosome-dependent degradation since silencing of HDAC6 or treatment with a specific HDAC6 inhibitor Tubacin reduced Notch3 protein expression and activity leading to apoptosis of T-ALL cells [321]. In urothelial cancer, another HDAC inhibitor, SAHA, downregulated Notch3 by increasing its acetylation with consequent proteasomal degradation, leading to cell cycle arrest. Considering that, in this tumor, Notch3 overexpression correlated with OS and that Notch3 silencing could counteract cisplatin resistance, Notch3 inhibition with SAHA could be applied as a potential therapeutic strategy [322].

Not only Notch3 but also Notch1 stability and degradation may be regulated by acetylation/deacetylation. Indeed, high expression of HDAC3 and the deacetylated state of Notch1 were associated with higher N1IC stability in T-ALL and CLL, whereas HDAC3 inhibition with Apicidin reduced N1IC protein levels and activity in Notch-dependent leukemic cell lines [323].

Notch protein stability may also be modulated by direct targeting E3 ubiquitin ligases responsible for Notch receptors degradation. In line with this, N-acetylcysteine (NAC) treatment inhibited glioblastoma growth upregulating the expression of E3 ubiquitin ligase ITCH, which mediates lysosome-dependent degradation of Notch2, proposing NAC as a Notch-targeting agent [324]. FBXW7 is another E3 ubiquitin ligase crucial for precise dosing of Notch activation that recognizes NIC phosphorylation at PEST domain and directs it to proteasomal degradation. This phosphorylation is driven by Cyc C-CDK8 recruited nuclear NIC by MAML [19]. Nuclear N1IC may also be phosphorylated by GSK-3β, which inhibits N1IC proteasomal degradation. Indeed, inhibition of GSK-3β with lithium chloride (LiCl) decreased N1IC stability and reduced Notch signaling activity [325]. On the contrary, other studies showed that GSK-3α/β phosphorylation can decrease Notch1 and Notch2 protein levels and their transcriptional activity [326,327], and treatment with LiCl increased N1IC levels [327]. These studies demonstrated that targeting GSK-3α/β might become a potential strategy to modulate Notch in cancer; however, this should be taken with caution, due to the complex interactions between these proteins and the opposite effects of LiCl treatment.

Notch3 activity can be negatively regulated through EGFR-mediated phosphorylation. Indeed, treatment with the EGFR inhibitor erlotinib reduced growth of EGFR-mutated lung cancer cell lines but favored stem-cell-like phenotype by enhancing Notch3 activation, while addition of GSI prevented selection of CSC, suggesting combined inhibition of EGFR and Notch3 in EGFR-mutated lung cancer as an optimal strategy to counteract the drug resistance mechanism [328].

Phosphorylation of Notch receptor intracellular regions may generate binding sites for prolyl-isomerase PIN1, positively regulating Notch signaling [329,330,331]. PIN1 enhanced Notch3 stability in Notch3-dependent T-ALL, and its silencing repressed leukemic cell invasiveness [329]. Likewise, in breast cancer, PIN1 increased Notch1 cleavage and N1IC and N4IC stability by inhibiting FBXW7-dependent proteasomal degradation, whereas *PIN1* silencing allowed to reduce the GSI dose necessary to suppress cell growth and CSC selection and to sensitize cancer cells to chemotherapeutic agents in vitro and in vivo [330,331]. Additionally, all-trans-retinoic acid, used for the treatment of acute promyelocytic leukemia treatment, suppressed breast cancer growth probably by inhibition of PIN1, being potentially an applicable approach for Notch inhibition when it is positively regulated by PIN1 [332].

The PTM-influencing strategies for Notch modulation are summarized in Table S1.

### 4.7. Natural Compounds as Notch Signaling Modulators

The anti-tumoral and Notch-antagonizing effects of flavonoids—natural compounds of phenolic structure present in fruits, vegetables, flowers, wine, and tea—have attracted a large amount of attention during the last decades (reviewed in [333,334]).

Xanthohumol (XN), a flavonoid isolated from the cones of hop plants (*Humulus lupulus L.*), inhibited tumor growth in in vitro models of breast, prostate, colon, hepatocellular, medullary thyroid, pancreatic, and ovarian cancer (reviewed in [335]). XN decreased cell viability through caspase-dependent and independent apoptosis and cell cycle arrest, and at least partially, its action was mediated through Notch inhibition [336,337]. In pancreatic cancer cells, XN reduced Notch1 expression and activity and induced apoptosis [335,338]; in breast cancer, it showed anti-proliferative, anti-metastatic, and pro-apoptotic effects [336] and enhanced anticancer Th1 immune response [339]. The same effects were also observed in ovarian cancer [340] and in BCR-ABL+ myeloid leukemia, where it counteracted the tissue-infiltrative capacity of malignant cells [341], as well as in other contexts (reviewed in [342]). Several clinical studies evaluating XN’s safety profile, antioxidant, and anti-inflammatory properties have been registered; however, its therapeutic applicability in cancer patients is yet to be elucidated.

Chalcones, another class of flavonoids, have shown anticancer, anti-inflammatory, and antimicrobial properties in numerous studies (reviewed in [343]). Butein and its derivative chalcone 8 suppressed endogenous Notch activity and induced cell cycle arrest and apoptosis in several T-ALL cell lines in a way different from GSI [344]. Additionally, not only classic chalcone scaffold but also chalcone-mimetic molecules sharing a distinct structural similarity with the maternal class of compounds reduced Notch signaling activation and T-ALL cell growth in vitro [345].

Similarly, the natural occurring phenolic compound juglone (5-hydroxy-1,4-naphthoquinone) had anti-Notch3 activities both in in vitro and in vivo leukemia settings, thus suggesting juglone-based therapies as potential approaches for the treatment of Notch3-dependent T-ALL [346].

Quercetin, another molecule of natural origin with pleiotropic capacities (reviewed in [347]), downregulated Notch, upregulated apoptosis, and reduced proliferation of HCC cells [348]. In colon cancer, treatment with quercetin affected cleaved Notch and Jagged expression and enhanced radiosensitivity by counteracting CSC growth in vitro and in murine xenograft models [349]. One more flavonoid luteolin decreased growth and invasion of breast cancer cells through Notch1 inhibition [350].

In addition, the natural compound honokiol (HNK) is also endowed with Notch-inhibitory potential. This molecule isolated from the roots, stem bark, and seed cones of Magnolia species has been used for the treatment of anxiety and stroke in traditional medicine; however, further studies have revealed its anti-inflammatory, anti-oxidative, and antimicrobial effects (reviewed in [351]). HNK inhibited Notch activation, induced cell cycle arrest, and possessed cytotoxic effect in melanoma models [352,353]. Additionally, HNK sensitized colon cancer cells to ionizing radiation through downregulation of Notch1 [354,355].

Curcumin is a natural polyphenol present in the rhizome of *Curcuma longa* (turmeric) and in other Curcuma species used in Asian countries for its antioxidant, antimicrobial, anti-inflammatory, and antineoplastic action. Curcumin and its derivatives have been recognized as molecules with good tolerability and safety profiles by the FDA (reviewed in [356]). Curcumin inhibited Notch1 activity, arrested the cell cycle in G0/G1 stages, and led to caspase-dependent apoptosis in prostate cancer cells [357]. Moreover, curcumin inhibited growth and invasion of osteosarcoma cells through downregulation of Notch1 and matrix metallopeptidase (MMPs) [358], and in oral SCC, it reduced the expression of Notch target genes such as MMPs, BCL-2 and NF-κB [359]. The already described effects of curcumin on cell proliferation and migration were observed also in NSCLC by affecting the EZH2/*Notch1* axis in a miRNA-dependent way [360]. An additional impact of curcumin on Notch1 signaling may be related to HDAC and p300 modulation [361]. The increasing evidence of antineoplastic effects of curcumin together with its relatively good safety profile encourages its use for cancer treatment as a single agent and in combination with other drugs due to multimodal modulation of Notch signaling.

Withaferin A (WA) is a steroidal lactone with anti-cancer and anti-inflammatory properties isolated from *Withania somnifera* (reviewed in [362]). WA inhibited Notch1 cleavage, downregulated AKT pathway in a Notch-downstream way, and induced apoptosis of colon cancer cells [363]. In ovarian cancer, the antiproliferative effects of WA were related to abolished Notch1 and Notch3 expression and AKT signaling inhibition [364]. Interestingly, in ovarian cancer, WA could synergize with doxorubicin potentially reducing the toxicity of high doses of this anthracycline antibiotic [365].

Additionally, other natural compounds have shown Notch-inhibitory potential in various cancer models, i.e., cucurbitacin B, which downregulated Notch1 signaling with consequent reduction of colon cancer growth in vivo and in vitro [366]; diallyl trisulfide, which showed antitumor and anti-inflammatory effects in a model of breast cancer associated with Notch inhibition and the additional advantage of tumor sensitization to doxorubicin [367]; epigallocatechin gallate [368]; genistein [369]; uscharin [370]; oleandrin [371]; and cowanin [372]. Moreover, recently it was demonstrated that the treatment with the non-toxic natural agonist 2-(1′H-indole-3′-carbonyl)-thiazole-4-carboxylic acid methyl ester (ITE) of the ligand-activated transcription factor aryl hydrocarbon receptor (AhR), which is widely investigated as a promising anti-cancer drug target, interferes with the Jagged1-dependent Notch pathway activation and counteracts proliferation, invasion, and migration of TNBC cells [373,374].

Of note, some plant-derived compounds have shown the ability to upregulate Notch signaling. N-methylhemeanthidine chloride found in *Zephyranthes candida* drastically upregulated Notch1 target gene expression and led to apoptosis in DLL4-stimulated AML experimental models [375]. Likewise, recently it has been demonstrated that Chrysin activated Notch1 signaling pathway, induced apoptosis, and inhibited cancer cell growth in in vitro and in vivo models of anaplastic thyroid carcinoma [376].

Natural compounds modulating Notch signaling are listed in Table S1.

## 5. Combining Notch Inhibitors and Conventional Chemotherapy

### 5.1. Notch and Alkylating Agents

Since their approval by the FDA in 1949, alkylating agents have been widely used as antineoplastic and immunosuppressive agents. Their mechanism of action is based on the covalent transfer of an alkyl group to the nucleophilic moieties of DNA resulting in replication blockage due to the presence of alkylated bases, defective DNA reparation of these lesions leading to mutagenicity or accumulation of single- and double-strand breaks [377]. Alkylating drugs are subdivided into several classes according to their chemical structure; here, we will review just the ones tested in combination with Notch-targeting agents.

#### 5.1.1. Nitrogen Mustards and Oxazaphosphorines

Nitrogen mustards such as melphalan, bendamustine, and chlorambucil are used for the treatment of hematologic malignancies, ovarian cancer, and refractory solid tumors. Oxazaphosphorines (cyclophosphamide, ifosfamide) are included in treatment protocols of various hematologic and solid tumors including sarcomas [377].

The mutational status of *Notch1/FBXW7* genes may influence the response of leukemia patients to treatment protocols including cyclophosphamide. Notably, acute lymphoblastic leukemia (ALL) *Notch1/FBXW7* mutated patients had a better response to the multiagent ALL BFM-95 protocol [378]. In the case of chronic lymphoblastic leukemia, the same mutations were associated with shorter progression-free survival (PFS) and OS and could serve as a predictive marker for decreased benefit from the addition of rituximab to cyclophosphamide [379]. However, in the UK LRF CCL4, which compared chlorambucil, fludarabine, and a combination of fludarabine with cyclophosphamide in previously untreated patients, *Notch1* mutational status was an independent marker that identified patients with intermediate outcome after initial therapy with DNA damaging agents [380].

Evidence supports that the addition of Notch-targeting agents to conventional treatment may contribute to overcoming stromal cell-mediated resistance of CLL cells to chemotherapy. Indeed, the coculturing of CLL cells with bone marrow stroma derived-cells mediated survival and anti-apoptotic mechanisms in the leukemic cells by upregulating IL7R, CD23, BCL-2, and NF-κB and downregulating the levels of active caspase-3. On the other hand, the combination of Notch inhibitors such as GSI and monoclonal antibodies, except anti-Notch3 and anti-DLL1, with alkylating drugs such as cyclophosphamide and bendamustine, counteracted stroma-dependent resistance of CLL cells to these drugs and reverted the above-mentioned molecular changes without affecting the viability of stromal cells [381]. The proteasome inhibitor Bortezomib, which exerted its antileukemic action through Notch1 downregulation, acted additively in combination with 4-hydroxycyclophosphamide in T-ALL cell models [382]. Moreover, the addition of diverse Notch-targeting agents might be useful to potentiate the effects of another alkylating agent, melphalan. Indeed, a combination of melphalan and the GSI MRK003 showed an additive/synergic effect on retinoblastoma cell lines, whereas GSI-XII significantly improved its cytotoxicity in multiple myeloma (MM) models, and Jagged1 and 2 inhibition reverted both intrinsic and stromal cell-induced resistance of MM to melphalan [383,384,385].

#### 5.1.2. Temozolomide

The Notch signaling pathway is one of the key processes involved in glioma development, and its hyperactivation contributes to tumor recurrence due to the persistence or selection of glioma stem-like cells (GSC), even if recently the oncosuppressive role of Notch signaling in glioma has emerged as well [130]. Generally, Notch signaling inhibition depleted GSC and inhibited the growth of tumor neurospheres and xenografts [386]. Temozolomide (TMZ) is a triazene derivative with alkylating properties that has the same active metabolite as dacarbazine and is commonly used for the treatment of brain tumors (gliomas, glioblastomas, and astrocytomas) [377]. Combination of TMZ and Notch-targeting agents (DAPT, RO4929097) decreased TMZ-resistant neurosphere recovery and extended tumor latency in murine xenograft models [387]. Interestingly, TMZ exposure upregulated transcriptional activity of the Notch pathway, and addition of the GSI-1 to TMZ had a synergistic cytotoxic effect on glioma cell lines [388]. RO-4929097 enhanced TMZ’s effect in ependymoma short-term cultures and glioma cells. Of note, RO-4929097-mediated effects were independent of *Notch1* mutational status but were associated with low IL6 levels [389]. However, co-treatment with TMZ and RO-4929097 reduced glioma stem cell markers expression (CD133, Sox2, Nestin) [189]. This combination was used in an early clinical setting. Patients who received RO-4929097 combined with TMZ and radiotherapy tolerated well the addition of a Notch-targeting agent to the treatment protocol. Despite the acceptable safety, modulation of Notch signaling, and decreased pool of stem-like CD133+ cells, some patients experienced tumor recurrence associated with upregulation of key mesenchymal genes and VEGF-dependent angiogenic factors; moreover, the efficient Notch signaling inhibition was observed in tumors with disrupted blood-brain barrier (BBB) (NCT01119599, Table 1) [390].

At the same time, simultaneous inhibition of Notch and VEGF signaling with a bispecific antiDLL4-antiVEGF antibody strongly improved tumor growth inhibition by temozolomide in a xenograft model of glioma, providing a possibility to affect both glioma-recurrence associated pathways with the addition of a single agent to chemotherapy [236].

#### 5.1.3. Platinum-Based Drugs: Cisplatin, Carboplatin, Oxaliplatin

Platinum derivatives (cisplatin, carboplatin, and oxaliplatin) are among the most effective alkylating agents commonly used for the treatment of various solid tumors, including testicular, ovarian, lung, esophagus, bladder, and head and neck epidermoid cancers in the case of cisplatin and carboplatin, and metastatic colon cancer for oxaliplatin [377].

##### Non-Small-Cell Lung Cancer

Notch inhibition in NSCLC affects the selected population of CSC, and it is upregulated by conventional therapy and implicated in the resistance mechanisms. One of them was linked to the overexpression of ABCG2 and ABCB1, granting multiple drug resistance to CSC; however, pre-treatment with DAPT significantly reduced cisplatin-mediated enrichment of drug-resistant CD133+ cells in NSCLC xenografts [186]. A similar effect was obtained after selective suppression of *Notch3* with silencing RNA (siRNA). Additionally, Notch3 inhibition counteracted the cisplatin-induced activation of the autophagosomal marker LC3-II in CSC, which is considered as an adaptive response to chemo- and radiotherapy [399]. Likewise, BMS-906024 synergistically increased spheroid growth delay of NSCLC cell lines when combined with cisplatin [400]. Similarly, the combination of BMS-906024 with cisplatin was synergic in an in vitro assay performed on 14 NSCLC cell lines and resulted in more effective PDX growth delay in vivo [401]. The results of two available CTs evaluating the combination of anti-DLL4 demcizumab and carboplatin/pemetrexed are discussed in Section 5.5. (Table 2).

##### Small-Cell Lung Cancer

Exposure of four SCLC cell lines to a concentration range of GSI PF-03084014 together with carboplatin resulted in additive or sub-additive action as assessed by the Bliss additivity method [402]. The phase II trial assessing the addition of etoposide and OMP-59R5 to cisplatin or carboplatin as the first-line therapy for extensive SCLC showed a lower frequency of disease progression or death in the group of tarextumab+etoposide addition to cisplatin/carboplatin compared to placebo during 1 year observation; however, it was not considered as an improved PFS, and it did not correspond to the increase in the frequency of ORR, confirming the controversial benefit of Notch inhibition in this lung cancer (NCT01859741). Moreover, the combination of Rova-T, composed of a human DLL3-specific mAb and the DNA-crosslinking agent and cisplatin + etoposide, did not add benefit to chemotherapy alone in terms of ORR and OS (NCT02819999), did not improve OS after the first-line platinum-based therapy (NCT03033511), and had worse OS and PSF compared to topotecan as the second-line approach (NCT03061812) [240,241,246]. A detailed description of the above-mentioned CT is shown in Table 2. The lack of inambiguous benefit of combining anti-Notch agents with cisplatin in this cancer may be related to the oncosuppressive role of Notch1 signaling [403]. Notably, the overexpression of DLL1 in SCLC increased cell sensitivity to cisplatin through induction of apoptosis and cell cycle arrest in G0/G1 phase [404].

##### Head and Neck Squamous Cell Carcinoma

Cisplatin is the most important chemotherapeutic agent used for the treatment of HNSCC; however, several Notch signaling-related mechanisms may result in resistance to this drug. High expression of Notch1 might be negatively correlated to HNSCC sensitivity to cisplatin [405]. Consistently, Notch1 inhibition sensitizes HNSCC cell lines to cisplatin [406] and enhances the efficacy of cisplatin by attenuating the population of chemotherapy-enriched CSC [407]. In particular, DAPT treatment reduced the CSC population by targeting the Notch1/HES1 axis that is often upregulated in HNSCC and is associated with higher expression of self-renewal markers such as CD44, Sox2, Slug, and ALDH1 [408]. On the other hand, an independent study showed that the targeting of the Notch4-HEY1 axis may sensitize HNSCC cells to cisplatin by preventing the upregulation of EMT-related genes [409]. Interestingly, some plant-derived compounds such as epigallocatechin-3-gallate could increase the sensitivity of HNSCC CSC to cisplatin by decreasing the expression of stemness markers and drug transporters in a Notch-dependent way [368].

##### Ovarian Cancer

In ovarian cancer, platinum-based drugs enrich the CSC population through activation of various stemness-related pathways including Notch signaling [410,411]. In line with this, overexpression of Notch target gene *HES1* promoted CSC characteristics and resulted in resistance to cisplatin. Reasonably, the addition of GSI MK-0752 counteracted these changes, providing a more evident synergistic effect if cisplatin administration was followed by Notch inhibition [412]. The same sequential advantage was found for the combination of eugenol and cisplatin, which was antagonistic when eugenol was added first in low concentrations; on the contrary, administering cisplatin followed by eugenol showed strong synergism. In the appropriate sequential combination, eugenol suppressed cisplatin-related enrichment of CSC, and its combination with cisplatin effectively downregulated drug-transporter expression and induced apoptosis in HES1+/CD44+ subpopulation resistant to single-agent treatments [413]. It is worth mentioning that sequential benefits of combining GSI and cisplatin might differ in case of cell lines with already-developed cisplatin resistance, as it was reported for pre-treatment with GSI DAPT, which increased the sensitivity of cisplatin-resistant cell lines through downregulation of both mRNA and protein levels of Notch1 and HES1, while cisplatin treatment followed by DAPT only presented an additive or antagonistic effect [414].

Among Notch receptors, high expression of Notch3 plays a particularly important role in ovarian cancer resistance to platinum-based compounds [415]. Illustrating this, carboplatin-induced ERK phosphorylation and apoptosis could be inhibited by Notch3 activation in some ovarian cancer cells [416]. Consistently, Notch3-modulating approaches have been successfully combined with cisplatin and carboplatin, increasing the DNA-damaging response and improving the sensitivity of cell lines and tumor xenografts to this agent [415,417,418]. The plant-derived substance mangiferin interfered with the activation of the Wnt/β-catenin pathway and induced cancer cell apoptosis in a Notch3-dependent manner, sensitizing ovarian carcinoma cells to cisplatin in time- and dose-dependent manners. In addition to this, it inhibited the activity of the upstream transcriptional regulator of Notch YAP [419,420]. Highlighting the possibility of targeting the upstream Notch regulators, an orphan receptor NR2F6 promoted the CSC phenotype and induced cisplatin resistance in epithelial ovarian cancer cells by interacting with *Notch3* promoter, localizing p300 there, enriching histone acetylation, and enhancing *Notch3* transcription. Both *NR2F6* knockdown, GSI, and *Notch3* knockdown helped to overcome cisplatin resistance in NR2F6-overexpressing cancer stem cells [421].

##### Osteosarcoma

A similar process is fair for osteosarcoma, in which cisplatin treatment selected CSCs through activation of Notch signaling [422]. In line with this, DAPT enhanced osteosarcoma cell line sensitivity to cisplatin, acted at least additively in combination with it, and downregulated pro-survival AKT and ERK signaling [423]. However, the effect of the concomitant Notch inhibition and cisplatin treatment is not so unequivocal, since in some osteosarcoma cell lines, Notch inhibition reduced available levels of pro-caspases 3, 8, and 9 and/or their activity [424].

##### Colon Cancer

Upregulation of Notch signaling (Notch1/HES1 axis, Notch2, and Jagged1) coherently with the modulation of major pro-survival pathways was associated with acquired resistance to oxaliplatin [302,425,426]. Biological synergy and mutual potentiation of the effects of each agent were evidenced when oxaliplatin was combined with GSI34, as oxaliplatin stimulated GS components’ expression, whereas downregulation of Notch signaling prevented the oxaliplatin-dependent induction of the pro-survival PI3K/AKT pathway [187]. Moreover, the knockdown of *JAG2* sensitized colon cancer cells to oxaliplatin by enhancing apoptotic cell death [427]. Another example of the shift of pro-survival pathways activation was described when inhibition of Notch1 with different GSIs led to transient activation of ERK 1/2 signaling that made the Notch1-positive subpopulation of colon cancer cells more susceptible to cisplatin-induced cell death [428]. In addition, the beneficial effects of combining oxaliplatin with Notch-targeting epigenetic modulators have been described. When oxaliplatin was combined with the JMJD3 inhibitor GSK-J4, the accumulation of H3K27me3 sensitized colorectal cancer to oxaliplatin through decreased transcription of *Notch2* [302]. Consistently, restoring the activity of the enzymatic counterpart of JMJD3 PRC2 through *STRAP* silencing counteracted transcriptional upregulation of *Notch1* and smothered stem-like features of colorectal cancer cells, resulting in tumor sensitization to oxaliplatin in vivo and in vitro [429]. Highlighting the controversial consequences of pro-survival pathways modulation after Notch signaling inhibition, the combination of GSI RO4929097 with oxaliplatin abrogated drug-induced apoptosis and improved survival of cancer cells, which was even more sustained by *HES1* silencing. This effect could be possibly explained by compensatory activation of other survival pathways [426]. In line with this, other GSIs such as MRK-003, DAPT, and GSI-XX reduced oxaliplatin-induced apoptosis in HTC116 colon cancer cells, increasing the levels of the anti-apoptotic BCL-2 family members MCL-1 and BCL-xL [430].

##### Breast Cancer

Lysine demethylase 2A (KDM2A) upregulated *JAG1* transcription to promote stemness, chemoresistance and angiogenesis in a TNBC model, whereas inhibition of its enzymatic activity enhanced the cytotoxic effect of cisplatin [413].

Other examples of beneficial targeting of Notch1 and Notch3 signaling for affecting CSC population and tumor sensitization to cisplatin treatment have been demonstrated in preclinical models of gastric cancer, neuroblastoma, hepatocellular carcinoma, renal cell carcinoma, cervical cancer, and nasopharyngeal carcinoma [431,432,433,434,435,436,437]. Additionally, in a phase I/II study, combination of GSI RO4929097 with two alkylating agents, cisplatin and temozolomide (+vinblastine), allowed for reaching PR or SD in 8 out of 14 patients with metastatic or recurrent melanoma, which correlated with reduced Notch cleavage in four out of five analyzed cases of OR (NCT01196416).

Moreover, an additional advantage of combining Notch inhibitors and cisplatin may be derived from the observation that Notch inhibitors could alleviate some systemic adverse effects of the platinum-based drug. Indeed, cisplatin may precipitate acute renal injury-causing apoptosis of tubular epithelium, and since it is excreted mainly through the kidney, the decreased renal function may result in its enhanced toxicity, as it happens in case of diabetic nephropathy. Cisplatin-induced kidney injury is associated with high levels of Notch1 activation, which in turn upregulates the inflammatory response and oxidative stress. DAPT-preconditioning of diabetic rats protected them from renal injury-inducing anti-inflammatory cytokines and upregulating antioxidant enzymes, and this protective effect was maintained in a combination of DAPT and cisplatin [438]. In addition to GSI treatment, *DLL1* knockdown attenuated Notch1 activation in kidney and prevented cisplatin-induced tubular necrosis [439]. Moreover, MDM2 inhibition disrupted Notch1 hyperexpression in cisplatin-induced kidney injury and alleviated the pro-apoptotic effects of this drug on tubular epithelium cells [440].

### 5.2. Notch and Microtubule-Targeting Agents

Microtubule-targeting agents interact with tubulins, disrupt microtubule/tubulin dynamics, and stop tumor growth. Traditionally, they are subdivided into two major groups: microtubule-stabilizing agents (taxanes, epothilones, and laulimalide) and microtubule-destabilizing agents (colchicine, vinca alkaloids, eribulin, nocodazole, and combretastatin A-4) [441].

#### 5.2.1. Vincristine

Vincristine is a vinca alkaloid approved for the treatment of several lymphoid malignancies, neuroblastoma, rhabdomyosarcoma, and Wilms tumor and used for some other off-label indications [442]. Reasonably, its combinations with Notch-targeting agents have been mostly evaluated in T-ALL with different functional consequences. An experimental study performed in several T-ALL cell lines did not show a significant advantage in terms of synergy or sensitization nor an antagonism in the case of combining the GSI Compound E and vincristine, both simultaneously and after pre-treatment with each drug [443]. Apart from the absence of a beneficial effect in GSI-sensitive T-ALL cell lines co-treated with vincristine and Compound E, GSI treatments were shown to antagonize the vincristine-induced apoptosis in GSI-resistant cell lines [444]. Nevertheless, an independent study demonstrated that DAPT as well as the GSI Compound E, DBZ, and L-685,458 increased vincristine-induced mitotic arrest and apoptosis, apparently in a Notch-independent fashion, in T-ALL cell lines and irrespectively of their GSI sensitivity. However, since at 48 h of treatment GSI did not cause cell cycle arrest, the observed enhancement of vincristine activity was described as sensitization and not synergism [445]. Notably, in solid tumors, the combined application of vincristine and DAPT affected the CSC population and improved the pro-apoptotic potential of the chemotherapeutic drug. Indeed, in hepatocellular carcinoma, pre-incubation with DAPT reduced the spheroid-forming and migratory capacity of CSC and sensitized them to vincristine treatment through enhancing BBC3-mediated apoptosis [446]. In addition to hepatocellular carcinoma, GSI DAPT enhanced vincristine-induced mitotic arrest in colon cancer cells and pancreatic ductal adenocarcinoma [447].

#### 5.2.2. Taxanes

The two microtubule stabilizers, paclitaxel extracted from the leaves of European yew (*Taxus baccata*) and its semisynthetic analog docetaxel, have been approved for the treatment of breast, ovarian, hormone-refractory prostate, pancreatic, esophageal, head-and-neck, and non-small-cell lung cancers [448]. Similar to other chemotherapeutic drugs, tumor recurrence after taxane therapy may be explained by the selection of CSC population, and it is believed that affecting CSC maintenance by targeting Notch signaling can provide notable advantages. Indeed, the addition of GSI or monoclonal antibodies against the NRR of Notch1 to docetaxel attenuated the CSC pool and sensitized cells to the chemotherapeutic treatment in experimental models of prostate cancer, breast cancer, NSCLC, and HNSCC [408,449,450,451,452]. Likewise, reduced occurrence of CSC also explained the beneficial effect proven by the combination of a cross-reactive Notch 2/3 antibody, OMP-59R5, and paclitaxel, which significantly decreased the growth of pancreatic, breast, ovarian, and SCLC xenograft tumors and delayed tumor recurrence following discontinuation of the chemotherapeutic agents [453].

Mechanistically, taxanes block cell cycle progression in the late G2/M phase by preventing mitotic spindle formation, and the prolonged mitotic arrest subjects cells to apoptosis [448]. Although the addition of GSI might enhance taxane-induced mitotic arrest and apoptosis, the advantage of combining microtubule-targeting agents and GSI might not be completely dependent on Notch signaling. Indeed, GSI DAPT, Compound E, and L-685,458 enhanced paclitaxel-induced cell cycle block of colon and pancreatic cancer cells but not of stomach and breast cancer cell lines [447,454], and the combination of DAPT and paclitaxel strikingly induced cyclin B1 levels, confirming the lacked activation of anaphase-promoting complexes. However, since the silencing of *Notch*/CBF1 did not enhance paclitaxel-induced mitotic arrest, the beneficial effects of γ-secretase inhibitors may not involve Notch signaling and would supposedly rely on the GS-independent functions of PSEN [447,454].

Moreover, the changes in tubulin dynamics under taxane treatment might favor Notch signaling activation. Indeed, paclitaxel promoted nuclear co-localization of α-/β_II_-tubulin and activated intracellular domain of Notch1 and augmented the CBF1-dependent transactivation activity of N1IC. Interestingly, this effect was not observed in the presence of colchicine [455]. Existence of these mechanisms provides one more rational basis for the addition of various Notch inhibitors, including the ones acting downstream the proteolytic cleavages to taxanes.

Below, we reported the results of preclinical and clinical studies combining taxanes and Notch-inhibitory molecules in different cancers.

##### NSCLC

Paclitaxel is part of the first-line chemotherapy of advanced NSCLC, and it provides an open field for the search of new drug combinations due to chemoresistance mechanisms switching on after prolonged treatment [456]. The addition of GSI to paclitaxel enhanced its antitumor effect in NSCLC preclinical models. Indeed, BMS-906024 improved spheroid growth delay under taxane treatment of NSCLC in in vitro experimental models [400]. Consistently, the combination of BMS-906024 and paclitaxel was characterized with notably synergic values of combination index (0.54) in several NSCLC cell lines. Of note, the synergy was greater in *KRAS-* and *BRAF*-WT cell lines and correlated with p53 status and low H_2_O_2_ pathway signature [401]. Consistently, with the role of Notch signaling in CSC selection, the pretreatment with DAPT increased the sensitivity of NSCLC cells to paclitaxel and negatively influenced the pool of CSCs selected by chemotherapy. In addition, the concomitant administration of DAPT or the selective inhibition of *Notch3* by siRNA and paclitaxel showed a synergic effect on promoting cancer cell death through activation of the intrinsic apoptotic pathway [186,457]. Moreover, the addition of DAPT effectively counteracted a Notch1-mediated mechanism of resistance to docetaxel related to increased multidrug transporter MDR-1 expression through the Notch1/AP-1/miR-451/MDR-1 signaling axis [449].

##### Ovarian Cancer

The combination of cisplatin and paclitaxel has been approved for the first-line treatment of ovarian cancer since 1996 [448]. Considering that the response to highly effective double therapy can be notably reduced through chemoresistance mechanisms, counteracting signaling underlying them is undoubtedly relevant. Indeed, GSI MRK-003 synergized with paclitaxel only in platinum-resistant ovarian cancer rather than in platinum-sensitive ones, supporting the critical role of Notch signaling in chemoresistance [458]. In particular, the CSC phenotype in ovarian cancer was associated with elevated Notch3 expression, and in the case of chemoresistant tumors, the effect of combined administration of paclitaxel and GSI-I was more dependent on Notch3 than on Notch1 due to its relatively higher expression and was associated with decreased viability, migration, angiogenesis, and CSC pool [459,460]. Likewise, the targeting of Notch ligands looked quite appealing, since *JAG1* knockdown sensitized ovarian cancer cells to docetaxel and it disrupted tumor angiogenesis in vivo at least in part by affecting the crosstalk with GLI2 [461]. The anti-DLL4 antibody demcizumab in combination with paclitaxel showed some signs of clinical benefit (CBR 42% expressed as PR and SD) and acceptably manageable toxicity in patients with recurrent platinum-resistant ovarian cancer in a phase Ib trial. Interestingly, two cases of PR and two cases of SD were registered in the sub-group of bevacizumab-pretreated patients (*n* = 5), providing an encouraging possibility of sequential use of the antiangiogenic drugs (NCT01952249) [221].

##### Breast Cancer

Cases of TNBC’s different sensitivity to GSI and its combination with paclitaxel may be related to the different status of *Notch* genes, as TNBC cell lines with *Notch1*-activating mutations were highly sensitive to GSI MRK-003 alone and in combination with paclitaxel, whereas cells with *Notch2* rearrangements were GSI-resistant [462]. Data on the association of Notch and HER2 expression in breast cancer are quite contradictory since both positive [463,464] and negative [465] relationships between these oncogenes have been reported; however, the interconversion between chemotherapy-sensitive HER2+/Notch1– and GSI-sensitive HER2–/Notch1+ circulating tumor cells underlay the in vivo efficacy of simultaneous treatment with GSI LY-411575 or RO4229097 and paclitaxel [466]. Notch1 is a poor prognostic factor responsible for CSC maintenance in breast cancer [467,468]. Indeed, Notch1 inhibition through miR-34a upregulation contributed to sensitization to paclitaxel in a breast cancer model affecting the pool of CSC [309]. It is worth mentioning that Notch1-related effects of taxane sensitization might be related to CSC-independent mechanisms, since *Notch1* knockdown notably enhanced growth inhibition and apoptosis induction by docetaxel through negative regulation of NF-κB DNA-binding activity [469]. Other molecular mechanisms mediating increased sensitivity to taxanes through Notch inhibition may include the restored expression of the Notch inhibitor Numb, abolished under taxane treatment, and the downregulation of MDR transporters, as it was found for the combination of GSI PF-03084014 and docetaxel [470].

The advantages of combining Notch signaling inhibition with taxanes could be explained by impaired tumor angiogenesis. In line with this, paclitaxel induced the generation of tumor-derived endothelial cells accompanied with DLL3 and Notch4 overexpression, and injection of GSI DAPT into xenografts derived from cells previously exposed to paclitaxel decreased the formation of tumor-derived endothelium, affecting Notch4-driven transcription of *VEGFR3* [471]. Additionally, the beneficial effects of combining luteolin, a naturally occurring flavonoid, with paclitaxel were mediated through the RSK/YB-1/Notch4 axis, confirming the prospective of increasing tumor susceptibility to apoptosis by Notch4 inhibition [472,473].

Since DLL4 could be adaptively upregulated by docetaxel, thus attenuating the cytotoxic effects of this agent [474], the addition of an anti-DLL4 monoclonal antibody, MMGZ01, reasonably enhanced this taxane efficacy in breast cancer xenografts through depleting the subpopulation of CSC, reversing the EMT, and inhibiting the formation of functional tumor vessels [475]. Considering the positive correlation between DLL4 expression and metastasis development in breast cancer, the addition of selective anti-DLL4 approaches could be useful to treat or prevent metastatic tumors [476]. Indeed, a bispecific anti-DLL4–anti-VEGF antibody notably improved the effects of paclitaxel on tumor growth inhibition and spontaneous lung and lymphatic node metastasis in a xenograft model of breast cancer, giving a good advantage over addition of anti-VEGF alone [236].

Early clinical trials with combinations of GSI and taxanes provided some evidence of moderate tolerability and efficacy. The evidence of beneficial combination of anti-Notch treatment with paclitaxel was reported in a phase I study assessing the safety of RO4929097 in patients with operable TNBC in combination with neoadjuvant paclitaxel and cisplatin, where 36% of enrolled patients achieved pathologic complete response, even if high doses of this GSI were often associated with neutropenia, thrombocytopenia, and hypertension (NCT01238133) [391]. An encouraging reduction of breast CSC pool was reported for the combination of the GSI MK-0752 and docetaxel in a xenograft model and in a CT involving patients with metastatic or locally advanced breast cancer, where this agent in a sequential combination with docetaxel possessed manageable toxicity and showed preliminary evidence of efficacy, such as decreasing the occurrence of stem cell phenotype in serial biopsies and providing a long disease stabilization in some participants (NCT00645333) [398]. Despite the promising preclinical results, PF-03084014, the third GSI tested in combination with docetaxel in patients with advanced breast cancer, showed moderate tolerability with reports of dose-limiting toxicity and limited preliminary clinical efficiency (four cases of CR and nine cases of SD out of 29 patients). The development of this molecule had been discontinued (NCT01876251, Table 1) [394].

##### Pancreatic Cancer

The role of different Notch proteins in pancreatic cancer is controversial, with some evidence supporting its function both as oncogene and oncosuppressor and indicating different patterns of expression of Notch paralogs and ligands [477,478,479]. Even if in preclinical studies the addition of a bispecific anti-Notch 2/3 antibody tarextumab to nab-paclitaxel and gemcitabine was associated with a greater antitumor effect, the same advantage was not repeated in a clinical setting. This is because the addition of anti-Notch 2/3 antibody tarextumab to nab-paclitaxel and gemcitabine in patients with metastatic pancreatic adenocarcinoma did not improve the clinical outcome, and the PFS was significantly shorter in the group of tarextumab-treated patients without any difference between the groups with different levels of Notch3 expression (NCT01647828, Table 2) [208,453]. Highlighting the controversial role of Notch signaling in pancreatic cancer, transcriptional reprogramming of cancer cells leading to elevated expression of Notch family proteins provided an advantage enhancing the selectivity and antitumor activity of oncolytic adenoviruses. Indeed, the combination treatment of pancreatic cancer cells with a Notch-responsive oncolytic virus that was strongly synergic with paclitaxel was revealed, with notable a reduction of the IC50 value compared with the drug alone and CI values of <1 [480].

##### HCC

The discovery of novel targeted approaches in HCC remains highly relevant since this tumor is not well responsive to standard chemotherapy [481]. Notch signaling affects neoplastic growth, invasion capacity, and the CSC properties of HCC, even if the impact of the four Notch receptors may differ [482,483]. The efficacy and safety of paclitaxel in HCC treatment is quite limited [484]; however, it is widely used in experimental models, and the few described examples of combining Notch inhibitors and taxanes in preclinical studies appear quite optimistic. A specific inhibitor of ADAM17, ZLDI-8, sensitized HCC cells to paclitaxel in vivo and in vitro, promoted accumulation of cells arrested in G2/M phase, and inverted the EMT phenotype, confirming the advantages of the Notch signaling blockade for potentiating taxol action [485]. A natural inhibitor of Notch-signaling rhamnetin, acting through upregulation of miR-34a, sensitized multiple-drug resistant cell lines of hepatocellular carcinoma to paclitaxel, decreasing its IC50 value when administered in combination and enhancing cell cycle arrest in G2/M phase [486].

##### Prostatic Cancer

The contribution of the Notch signaling pathway in prostate carcinogenesis is ambiguous since major evidence supports its association with the invasiveness, EMT, CSC maintenance, and more aggressive androgen-independent or castration-resistant phenotype, but its oncosuppressive role in heterogenous prostate tumors is not excluded [487]. Indeed, Notch1 levels inversely correlated with the expression of E-cadherin in paclitaxel-resistant prostatic cancer, and GSI-mediated inactivation of Notch1 and Notch4 reversed the sensitivity of cancer cells to this taxane. On the other hand, *Notch1* silencing promoted docetaxel-induced growth inhibition that was associated with the downregulation of p21(waf1/cip1), BCL-2, and AKT expression and the upregulation of BAX [488,489,490]. Similarly, the GSI PF-03084014 reversed docetaxel resistance, increasing the chemotherapy-induced apoptosis and suppressing EMT, tumor angiogenesis, and CSC population. Mechanistically, PF-03084014 prevented the upregulation of critical pro-survival cascades such as PI3K/AKT, EGFR, NF-κB, BCL-2, and BCL-xL [452,491]. At the same time, even if a monoclonal anti-Notch1 antibody OMP-A2G1 induced apoptosis alone and in combination with docetaxel in an androgen-independent prostate cancer model and suppressed tumor cell proliferation in an androgen-sensitive cell line, there was no additive or synergic interaction between the two agents. Indeed, OMP-A2G1 alone inhibited tumor growth to a greater extent than docetaxel alone, which inspires the thought that specific Notch1 inhibition may not be sufficient to potentiate taxanes’ therapeutic effect in this cancer [492]. Interestingly, other Notch receptors could contribute to the crosstalk with major cancer stemness regulators such as the Hedgehog pathway, as it was demonstrated that the silencing of *Notch2* or Notch target genes *HES1* and *HEY1* depleted the population of Notch/Hedgehog overexpressing tumor-initiating cells in a cell model of prostatic cancer with acquired resistance to docetaxel [493].

Additionally, some advantages of combining GSI or nature-derived compounds with Notch-inhibitory activity such as isoxanthohumol were reported also for uterine serous carcinoma and melanoma models [494,495].

### 5.3. Notch and Anthracyclines

Anthracycline antibiotics are one of the most-used chemotherapeutics and are highly effective in various malignancies. Their mechanism of action is based on topoisomerase II poisoning, resulting in enzyme-mediated DNA damage and generation of double-strand breaks with consequent activation of DNA-damage response and p53 pathways. Additionally, DNA intercalation, oxidative stress induction, and chromatin damage through histone eviction contribute to the cytotoxic action of these agents [496].

#### 5.3.1. Hematological Malignancies

Anthracyclines are indicated as a part of the first-line treatment of acute lymphoid and myeloid leukemia and various lymphomas. In addition, a possible non FDA-approved indication may be considered for multiple myeloma (MM) and Waldenstrom macroglobulinemia [497].

The existing evidence of the interaction between direct Notch inhibitors and anthracyclines is limited and ambiguous since the combination of daunorubicin and GSI lacked any additional effects in GSI-sensitive T-ALL cell lines and was antagonistic in GSI-resistant T-ALL cell lines through the upregulation of the anti-apoptotic BCL-xl [444]. On the other hand, sensitization of T-ALL cell lines to the Wee1 checkpoint kinase inhibitor MK-1775 and enhanced induction of apoptosis were related to the inhibition of Notch1 and not mTOR signaling, indicating that the consequences of combining doxorubicin and indirect Notch inhibitors in the same cancer context may be different [498]. Indeed, bortezomib, a proteasome inhibitor that exerted its antileukemic action through Notch1 downregulation, was highly synergistic with doxorubicin in several cell models of T-ALL [382].

Moreover, combining GSI and anthracyclines may be useful to prevent stroma-dependent chemotherapy resistance. In line with this, GSI-XII prevented bone marrow stroma-mediated protection of multiple myeloma cells from doxorubicin-induced apoptosis in cellular and tumor xenograft models. Possibly, GSI counteracted activation of Notch following MM cell interaction with stroma and prevented the accumulation of HES1, thus derepressing the transcription of proapoptotic regulator Noxa [385]. However, MM resistance to doxorubicin might also correlate with low levels of HES1, even if when they are accompanied by elevated expression of Notch2 and Jagged ligands [499].

On the contrary, in acute myeloid leukemia where Notch proteins might be oncosuppressive, the resistance to doxorubicin was associated with elevated levels of NUMB and low expression and activity of Notch2 and Notch3 [500].

#### 5.3.2. HCC

Doxorubicin may be used for chemoembolization therapy in unresectable hepatocellular cancer [501]. In hepatocellular carcinoma, Notch3 rather than Notch1 contributed to the doxorubicin resistance, and the advantage of combining anti-Notch3 treatment and doxorubicin for DNA damage and apoptosis induction was related to increased p53 expression and failed to be beneficial in p53-/-, consistent with the known role of p53 inactivation in conferring HCC resistance to doxorubicin [502,503]. A more eminent effect of combining Notch3 rather than Notch1 inhibitors with doxorubicin in p53-WT HCC may be explained by the observation that Notch1 could prevent AKT-mediated proteasomal degradation of p53 in p53-WT but not in p53-mutated cell lines [504]. Interestingly, also in glioblastoma, GSI synergized with doxorubicin only in cells harboring WT p53 [505]. At the same time, overexpression of miR-760, normally suppressed by doxorubicin treatment, mitigated HCC resistance to this chemotherapeutic drug through inhibiting Notch1 and promoting PTEN expression, demonstrating that inhibition of Notch1 might be re-sensitizing in this tumor as well [506]. The contrasting evidence on Notch1 inhibition in combination with doxorubicin may be explained by the ambiguous role of Notch1 in HCC progression which can assume either an oncogenic or oncosuppressive function, whereas Notch3 seems to be more concordantly pro-tumoral. Moreover, p53 gene status and AKT pathway signatures should be considered [52].

#### 5.3.3. Breast Cancer

Doxorubicin is a principal component of the first-line therapy against breast cancer not lacking the chemoresistance problem, and several mechanisms of tumor resistance to this anthracycline are linked to Notch signaling, making it an appealing additional target [507]. Indeed, inhibition of Notch1 with siRNA or GSI DAPT enhanced growth inhibition and apoptosis induction by doxorubicin in breast cancer cells accompanied with the inactivation of NF-κB, induction of PTEN, and abolishing doxorubicin-induced upregulation of MDR-1 [185,469,508]. Moreover, an anti-Notch1 monoclonal antibody increased the sensitivity of breast cancer cells to doxorubicin through depleting the population of CSC [260]. In addition, co-loaded nanoparticles containing doxorubicin and the Notch1-inhibitor miR-34a affected TNBC cell migration more effectively and allowed enhanced tumor growth suppression compared with the drug alone in vitro and in vivo [509]. However, the single silencing of *Notch1* could be not sufficient to regain breast cancer cell sensitivity to doxorubicin; therefore, targeting multiple pathways including STAT3 and β-catenin together with Notch1 could provide a more synergistic action with doxorubicin [510,511]. Targeting the DLL3/Notch4 axis instead allowed to affect tumor-derived endothelial cells and neoplastic angiogenesis upregulated under doxorubicin therapy by reducing Notch4-driven transcription of *VEGFR3* [471].

#### 5.3.4. Osteosarcoma

Doxorubicin is included in the standard-of-care chemotherapy of osteosarcoma [512]. Exposure of osteosarcoma cells to sub-lethal doses of doxorubicin upregulated Notch1 signaling and promoted the EMT, whereas treatment with GSI was able to prevent these changes [513]. At the same time, consistently with the quite equivocal benefits of combining Notch inhibition with cisplatin for osteosarcoma treatment, knockdown of *Notch1* in this tumor model reduced the cytotoxic effects of doxorubicin partially related to the upregulation of *Notch1* and its target genes [514].

Other successful combinations of Notch signaling inhibitors with doxorubicin have been reported for ovarian, NSCLC, and colon cancer, where the addition of a Notch inhibitor allowed to reduce the CSC population and to increase autophagy and ROS production [186,365,427,515]. Several mechanisms of chemoresistance, including multidrug resistance drug transporters, upregulation of prosurvival pathways, stabilization of EMT phenotype, and selection of CSC, decrease tumor cell sensitivity to doxorubicin [516]. The Notch transcriptional program takes an important and well-recognized place in all these mechanisms, and it explains the frequent yet context-dependent benefit of combining Notch signaling inhibitors and anthracyclines. Interestingly, the defective DNA repair (homologous recombination and nucleotide excision repair in particular) might notably influence the efficiency of doxorubicin [517]. Notch signaling participation in DNA damage response has been evidenced on several levels. In T-ALL, Notch1 directly inhibited ATM kinase activity contributing to survival of Notch1-driven leukemias through impaired formation of FOXO3a-KAT5/Tip60 complex [518,519]. On the other hand, in BRCA-deficient TNBC, Notch1 affected DNA damage response in a pro-survival way by enhancing phosphorylation of ATR [520]. Interestingly, in FANCA-mutated Fanconi anemia characterized by pancytopenia and chromosomal instability due to dysregulated DNA repair, Notch1 overexpression facilitated defective hematopoietic cell proliferation [521]. The existence of linking elements between Notch signaling and DNA repair mechanisms encourages us to think that the crosstalk between these pathways and Notch signaling should not be omitted when designing drug combinations between Notch inhibitors and DNA-targeting agents.

Another disadvantage of combining Notch inhibitors and anthracyclines should be mentioned since some cardiac adverse events have been described in the case of these agents’ co-administration. Since Notch signaling is implicated in cardiac protection following doxorubicin treatment, administration of GSI DAPT inhibited the release of N1IC and mitigated myocardial repair following doxorubicin-mediated heart injury, and inhibition of Jagged1 or Notch1 eliminated the antisenescence effects of mesenchymal stem cells on cardiomyocytes that underwent doxorubicin treatment [522,523,524]. Moreover, elevated levels of Notch1 in serum might be considered as candidate early biomarkers of doxorubicin toxicity because pretreatment of mice with cardioprotective substance dexrazoxane attenuated doxorubicin-induced elevated levels of Notch1 and mitigated its cardiotoxicity [525]. On the other hand, the suppression of the Notch1-Snail axis in podocytes prevented EMT, relieved glomerular structural disruption, and reduced proteinuria caused by doxorubicin [526].

### 5.4. Notch and Topoisomerase Inhibitors

#### 5.4.1. Podophyllotoxins: Etoposide and Teniposide (Topoisomerase 2 Inhibitors)

Etoposide is a topoisomerase II inhibitor acting in late S and G2 phases of cell cycle and is currently FDA-approved for SCLC and testicular cancer and used for the treatment of several other malignancies such as NSCLC; lymphomas; AML; prostatic, ovarian and hepatocellular cancer; and refractory pediatric tumors [527].

A combination of etoposide and cisplatin or carboplatin is the first-line chemotherapy for SCLC [528]. Interestingly, preclinical studies in several SCLC cell lines indicated the additive effect of the GSI PF-03084014 in combination with etoposide, as assessed by Bliss additivity method [402]. However, addition of the Notch 2/3 targeting antibody tarextumab and etoposide to carboplatin or cisplatin in patients with SCLC receiving these drugs as the first line treatment did not improve the frequency of OR compared to placebo (NCT01859741). It is worthful mentioning that SCLC is often associated with the inactivation of oncosuppressive Notch1 signaling [403].

On the other hand, optimistic evidence of combining anti-Notch agents and etoposide may be revealed in several cancer models. BMS-906024 synergistically decreased the spheroid growth delay of NSCLC cell lines when combined with etoposide [400]. In cases of breast cancer, p53-mediated upregulation of Notch1 expression might counteract the proapoptotic effects of p53 and blunt the action of genotoxic agents, and the addition of GSI to etoposide increased etoposide-induced apoptosis in p53-WT breast cancer cells [529]. In HCC, where etoposide may be used for chemoembolization, a natural inhibitor of Notch signaling rhamnetin sensitized multiple-drug resistant cell lines of HCC to etoposide through the upregulation of the Notch inhibitor miR-34a and enhanced cell cycle arrest in S/G2 phase [486]. In addition, an ADAM17 inhibitor, ZLDI-8, sensitized HCC to etoposide in vivo and in vitro and notably enhanced cell cycle block in S phase [485].

However, contrasting evidence exists in other cancer contexts. A combination of the GSI compound E and etoposide was antagonistic in a T-ALL model in vitro, since Notch1 inhibition partially protected T-ALL cells from etoposide-induced cell death and diminished the IKK contribution to etoposide-induced T-ALL cell apoptosis. Additionally, the expression of the Notch target genes *MYC* and *HES1* inversely correlated with the expression of the pro-survival *BCL-2* and *BCL-xL* [530]. Moreover, the addition of GSI MRK-003 and RO4929097 did not increase the cytotoxic effects of etoposide in cellular models of colon cancer and glioma, respectively, despite the existence of a molecular basis between tumor progression and Notch signaling in both cases [389,430,531]. Of note, a specific mechanism of resistance to etoposide mediated by the 5′-tyrosyl DNA phosphodiesterase (TDP2) has not been directly linked with Notch signaling yet [532].

Complicating even more the ambiguous interaction between Notch-targeting approaches and etoposide in the above-mentioned cancers, it should be noticed that a forced activation of Notch signaling with hD1R peptide (a DLL1 fragment linked to an endothelium-recognizing part) had a strong antiangiogenic effect and acted additively in combination with teniposide and cisplatin in glioma, breast cancer and NSCLC cells [533].

#### 5.4.2. Camptothecin Analogues: Irinotecan and Topotecan (Topoisomerase I Inhibitors)

Irinotecan is a DNA topoisomerase I inhibitor used against a variety of solid tumors, such as colorectal, pancreatic, ovarian, and lung cancers; however it is mostly known for its use in colorectal cancer first- and second-line treatment protocols [534]. The results of preclinical experiments evaluating the combinations of irinotecan with Notch inhibitors looked quite promising. Even if GSI MRK-003 did not affect apoptosis of irinotecan-treated colon cancer cells, GSI34 sensitized cancer cells to chemotherapy and was synergistic with the active metabolite of irinotecan SN-38 [187,430]. Moreover, a combination of the GSI PF-03084014 and irinotecan effectively reduced tumor growth in colon cancer preclinical explant model and suppressed the growth of ALDH+ tumor-initiating cells [535]. An ADAM17 inhibitor ZLDI-8 improved the anti-tumor activity of irinotecan in colon cancer cell lines acting in near-additive way and counteracting the induction of Notch1-4 expression by irinotecan [536]. It is worth mentioning that in addition to upregulation of Notch receptor expression after irinotecan treatment, elevated levels of the Jagged1 ligand might contribute to the resistance of colon cancer cells to irinotecan [425]. Moreover, targeting DLL4 looked like as an appealing strategy as an anti-DLL4 antibody was efficacious against both WT and mutant *KRAS* in combination with irinotecan, decreasing the population of colon cancer stem cells and promoting apoptosis in tumor cells both in vitro and in xenograft models [222,537]. The beneficial effect of combining anti-DLL4 and bispecific anti-DLL4/VEGF antibody with irinotecan was further confirmed in xenograft and orthotopic mouse models of gastric cancer and colon cancer [236,538].

### 5.5. Notch and Antimetabolites

#### 5.5.1. Folic Acid Antagonists

Folic acid antagonists disrupt metabolic pathways requiring one-carbon moieties such as methionine synthesis or purine and thymidine synthesis. Methotrexate is a cytostatic and immunosuppressive agent that inhibits DHFR (dihydrofolate reductase) and is used for the treatment of hematologic malignancies, several cancers, and sarcomas. Pemetrexed acts on thymidylate synthase and DHFR and is used in NSCLC and pleural mesothelioma chemotherapy [539]. Unfortunately, to our knowledge, these agents have not been abundantly studied in vitro in combination with Notch inhibitors in cancer models; therefore, we reviewed the results of few available experimental and clinical studies. DAPT did not increase methotrexate-induced apoptosis in GSI-resistant T-ALL cell lines [445]. It was suggested that the knockdown of *Disheveled-3* re-sensitized colon cancer cells to methotrexate by attenuating Notch1 signaling; however, this molecular marker was considered as a signature associated with CSC [540]. The addition of BMS-906024 did not increase the efficiency of spheroid growth delay induced by pemetrexed in cell lines of NSCLC [400]. Despite this, the combination of anti-DLL4 demcizumab and pemetrexed (+carboplatin) was evaluated in phase IB CT, where this association of drugs caused OR in 50% of patients (CR, PR, SD); however, PSF and OS were not different from the ones expected for chemotherapy alone, and moreover, the levels of proangiogenic regulators LEF1 and SFRP2 in blood of treated patients appeared to be increased, suggesting a possible resistance/relapse mechanism (NCT01189968) [226]. Indeed, the results of a phase II placebo-controlled trial showed no advantage of demcizumab addition to pemetrexed and carboplatin in terms of PR and SD compared with placebo (PR and SD frequency in placebo and two demcizumab arms of trial, respectively: 52% and 40%, 35.7% and 50.0%, 20.7% and 51.7%) (NCT02259582). Despite the axiomatic role of Notch signaling in various tumor resistance mechanisms, we did not find a direct description (based on experimental data and not on bioinformatic prediction) of Notch signaling status associated with methotrexate and pemetrexed treatment in the above-mentioned studies. Moreover, the upregulation of Notch1, Notch3, and Notch target genes after supplementation of folic acid or 5-MTHF or after folate receptor overexpression in physiological and tumoral context suggests a deeper evaluation of the link between the folate metabolism and Notch signaling [541,542,543].

#### 5.5.2. Pyrimidine Antagonists

##### Fluoropyrimidines: 5-Fluorouracil, Tegafur, Capecitabine

5-fluorouracil (5-FU) is an analog of uracil extensively used for the treatment of different tumors. Mechanistically, 5-FU interferes with the activity of the thymidylate synthase and disrupts the DNA and RNA synthesis by incorporating at the place of the pyrimidine bases, thus leading to cell death. Likewise, the oral 5-FU prodrug Capecitabine, which is converted into its active form 5-FU preferentially by tumor cells, is mostly used for the treatment of colorectal cancer and advanced forms of several other types of cancer [544]. Although 5-FU is the key component of colorectal cancer (CRC) chemotherapy regimens FOLFOX and FOLFIRI, the severe adverse reaction associated with the drug and acquired resistance after chemotherapy limit its clinical application. Mechanisms of tumor resistance to this drug have been abundantly studied but not completely overcome [545]. Notch signaling is frequently involved in these resistance mechanisms, including more generic ones such as EMT, multidrug resistance transporters, and CSC maintenance, and more specific ones such as epigenetic alterations, which makes it a valuable target to hit together with 5-FU. It is worth mentioning that the expression of Notch-induced transcriptional factors *HEY1*, *HES1*, and *SOX9* correlated with poorer outcomes in 5-FU-treated CRC patients [545,546]. In line with this, the utility of combining different approaches aiming against Notch receptors, ligands, and regulators with 5-FU has been evidenced by numerous preclinical studies. Indeed, the upregulation of the Notch1/HES1 axis and Jagged1 was associated with acquired resistance to 5-FU in colon cancer cells; co-treatment of colon cancer cells with GSI34 in CRC cells was synergistic with 5-FU [187,425,426,547]; and the knockdown of *JAG1* and *JAG2* sensitized colon cancer cells to 5-FU and enhanced the induction of apoptosis [427]. In particular, the tumor sensitization to 5-FU by *Notch1* knockdown was mediated through p27 upregulation [548], and inhibition of Notch1 by DAPT affected the 5-FU-chemoresistant population of CRC cells to a higher extent compared with the parental one through targeting the pool of CSC [549]. Additionally, the ADAM17 inhibitor ZLDI-I, by preventing 5-FU-mediated upregulation of Notch1-4, improved the anti-tumor activity of 5-FU and reversed EMT, acting in a synergic or additive way in different concentrations [536].

Improved sensitivity of colon cancer cells to 5-FU was achieved also through targeting the epigenetic machinery with *STRAP* silencing that restored PCR2 inhibitory activity on *Notch1* expression [429]. Not only *Notch1* but also *Notch3* knockdown suppressed spheroid formation as well as Oct-4 and Lgr5 expression and improved 5-FU resistance in another colon cancer cell model [550]. Moreover, considering that 5-FU affects RNA synthesis and microRNA expression profile [551], it seems reasonable to look through these changes and to select the bridging elements between 5-FU resistance mechanisms and Notch signaling regulators. Indeed, low levels of the negative Notch regulator miR-34a were associated with a worse response of colon cancer patients to 5-FU, and its overexpression overcame ABCG2-mediated resistance of a stem cell-like subpopulation of colon cancer cells to 5-FU through downregulation of DLL1/Notch signaling. Similarly, the overexpression of miR-195-5p and miR-139-5p sensitized colon cancer cells to 5-FU through decreased expression of Notch2/RBP-jκ and Notch1, respectively [552,553,554,555].

On the contrary, in another experimental setting, combining the MAPK (mitogen-activated protein kinase) and Notch inhibitors selumetinib and dibenzazepine, respectively, with 5-FU did not result in a significant increase in therapeutic response of colon cancer xenograft tumors compared to the single therapy, likely due to the decrease in tumor cell proliferation upon MAPK and Notch inhibition, leading to reduced effectiveness of cytotoxic treatment [556]. Interestingly, the same authors demonstrated that the combination of selumetinib and dibenzazepine downregulated the expression of thymidylate synthase (TS), but it did not improve 5-FU sensitivity. However, considering that elevated expression of TS is recognized to be an important specific mechanism of tumor resistance to 5-FU [557], unveiling the influence of specific Notch inhibitors on TS expression would be of a great interest.

Positive evidence of combining Notch inhibitors with 5-FU was obtained from some other cancer models. In HCC, DAPT and *Notch2* knockdown increased sensitivity to 5-FU by affecting CSC pool and upregulating apoptosis through the Notch/HES1/BBC3 axis [446,558]. Notch1-mediated CSC pool reduction responsible for the sensitization to 5-FU was described also for HNSCC, whereas in intrahepatic cholangiocarcinoma, it decreased the expression of multidrug-resistance proteins [444,559]. In gastric cancer, high DLL4 levels reasonably resulted in hyperactivation of Notch1 signaling and were associated with poorer clinical outcome, stem-cell-like phenotype and 5-FU resistance [560]. The addition of Notch inhibitor to 5-FU and chloroquine decreased the viability of gastric CSC, highly expressing Notch1 and autophagy markers [561]. Notably, the synergistic or additive effects of combined administration of GSI-I and 5-FU in gastric cancer experimental models were linked to non-competitive cell-cycle blocking mechanisms mediated by each drug (G2/M arrest for GSI-I and S-phase arrest for 5-FU), increased apoptosis, and negative regulation of cell survival pathways such as MAPK-related signaling [562].

Several early-stage clinical trials evaluating pyrimidine antagonists alone or as a part of FOLFIRI (irinotecan, leucovorin, 5-FU) protocol in combination with the blocking antibodies against Notch ligands for the treatment of CRC and other solid tumors have been registered (NCT03031691, NCT01189942, NCT03035253, NCT03368859, NCT01946074, NCT01158274, Table 2). In the case of capecitabine, a confirmed PR was achieved in few patients with fluoropyrimidine-refractory colon cancer and cervical cancer treated with a combination regimen of capecitabine and the GSI RO4929027. RO4929027 administration was associated with dose-limiting toxicity and auto-induction at high doses (NCT01158274) [393]. Unfortunately, the only completed phase 2 RCT of FOLFIRI+ABT-165 (a bispecific anti-DLL4/VEGF antibody) in pretreated patients with metastatic colon cancer (NCT03368859) showed a worse ORR and higher frequency of serious adverse events compared with the addition of anti-VEGF alone. Notably, in one of the above-mentioned preclinical studies, the FOLFIRI combination reduced Notch1 expression in colon cancer cells, which could be a possible explanation underlying the lack of expected efficacy of adding a Notch inhibitor to this chemotherapeutics combination [556].

##### Cytarabine

Cytarabine (Ara-C) is a pyrimidine nucleoside analog commonly used in multiagent chemotherapy protocols for the treatment of leukemia and lymphoma [563]. Ara-C combinations with Notch inhibitors looked more promising in B-ALL than in T-ALL; however, the existing evidence is quite limited. DAPT did not increase cytarabine-induced apoptosis in GSI-resistant T-ALL cell lines [445]. Bortezomib, a proteasome inhibitor that exerted its antileukemic action partially through Notch1 downregulation, had additive action in combination with cytarabine in several cell models of T-ALL, but as in cases of other non-specific Notch inhibitors, an additional anti-viability advantage could be attributed to the modulation of other molecular pathways [382]. On the other hand, the treatment with GSI-XII, DAPT, and the blocking antibody against Notch4 in B-ALL potentiated the anti-viability and pro-apoptotic effects of cytarabine in an ROS-dependent way. In addition, the co-administration of GSI-XII and cytarabine lowered the bone marrow leukemic burden in a murine xenograft model of B-ALL [564], while DAPT reduced central nervous system infiltration in a B-ALL murine model and led to increased chemosensitivity of leukemic cells to Ara-C by impairing their interaction with choroid plexus stroma expressing high levels of Jagged1 and ADAM10 [565].

##### Gemcitabine

Gemcitabine is a cytidine analog, and its diphosphate and triphosphate intracellular modifications inhibit ribonucleoside reductase and DNA polymerase, respectively, leading to the depletion of deoxyribonucleotide pool and the blockage of DNA synthesis [566]. It is used as a first-line treatment of pancreatic cancer and as a part of combined therapy for advanced bladder cancer and NSCLC [567,568]. It can be also considered for biliary tract cancers and some other malignancies [569]. The combination of gemcitabine and Notch-targeting agents has been abundantly studied in pancreatic cancer. Different Notch receptors and ligands contribute to EMT, CSC maintenance, stroma-induced resistance, and to the crosstalk with survival pathways such as NF-κB and PI3K/AKT, favoring the survival of resistant pancreatic clones [570]. Reasonably, combinations of different GSI and Notch-targeting antibodies affected various mechanisms of gemcitabine resistance and improved its anti-viability effects in in vitro experiments.

Gemcitabine enriched the CSC population in pancreatic cancer and led to the activation of the Notch signaling pathway in pancreatic cancer cell lines. In line with these observations, the addition of the GSIs DAPT or PF-03084014 in gemcitabine-treated pancreatic cancer cells significantly reduced the CSC pool and improved the growth inhibitory effects of the treatment through the reactivation of the intrinsic apoptosis pathway and by reversing the upregulation of the pro-survival pathways β-catenin and pAKT [571,572,573,574]. Interestingly, the combination of a Notch-responsive oncolytic virus and gemcitabine was synergic in pancreatic cancer cell lines, as confirmed with evident reduction of IC50 value in co-treated cells compared with the drug alone and CI values <1 [394]. The combination of MRK003 with gemcitabine prolonged the survival of mice with pancreatic ductal adenocarcinoma xenografts by targeting tumor endothelial cells and promoting tumor hypoxic necrosis [575]. The same combination potentiated gemcitabine efficiency through downregulation of nuclear Notch1 intracellular domain, inhibition of anchorage-dependent growth and reduction of CSC pool. Interestingly, NF-κB upregulation was predictive of good sensitivity to MRK-003, whereas upregulation of the NRF2 pathway and B-cell receptor signaling correlated with a good response to the drug combination [576].

Notch2 receptor overexpression has been related to chemoresistance and EMT in pancreatic cancer, and high Notch3 expression is considered as a predictor of poor survival in pancreatic cancer patients, whereas low expression of Notch3 correlated with longer OS in patients with unresectable pancreatic cancer and could serve as a predictive biomarker of gemcitabine efficiency [577,578]. In line with this, some regulatory circuits explaining Notch2 and Notch3-mediated gemcitabine resistance have been described. Indeed, Gemcitabine treatment increased the expression of a heparin-binding growth factor, Midkine, which critically activated Notch2 and NF-κB signaling, whereas knockdown of both *MK* and *Notch2* sensitized pancreatic cells to gemcitabine [118]. Likewise, *Notch3* knockdown decreased the average IC50 of gemcitabine through inactivation of the PI3K/AKT pathway [579], and consistently, the combination of a cross-reactive Notch 2/3 antibody with gemcitabine drastically reduced the frequency of CSC, sensitized tumorigenic cells to the cytotoxic effects of the chemotherapy and, apart from more efficient reduction of tumor growth, delayed tumor recurrence compared with single agents in a pancreatic cancer xenograft model. Interestingly, treatment efficiency correlated with Notch3 rather than Notch1 and Notch2 expression, determining the difference between responders and non-responders [453].

Low DLL4 expression was associated with longer OS in patients with pancreatic ductal adenocarcinoma [580]. Accordingly, in another model of pancreatic cancer, DAPT restrained the effects caused by DLL4-induced Notch activation, and pre-treatment with a high dose of DAPT abrogated DLL4/Notch-induced chemoresistance led to the activation of apoptosis and reduced the CSC pool [581]. The combination of anti-DLL4 and gemcitabine had additive antitumor activity in pancreatic cancer xenograft models and delayed tumor recurrence after termination of gemcitabine treatment targeting DLL4 both in tumor and in stroma/vasculature [582]. More evidence of the beneficial effects of concomitant Notch inhibition in overcoming gemcitabine resistance was observed also in vitro, as the co-culture of pancreatic cancer cell lines with pancreatic stellate cells conferred chemoresistance to gemcitabine to cancer cells, while the GSI L1790 or *HES1* knockdown reversed the stroma-induced drug resistance [583].

Preclinical evidence of combinations between gemcitabine and Notch inhibitors in other cancer models is not so detailed. In cholangiocarcinoma, Notch 1-3 expression was associated with lower histological differentiation and poorer survival of patients, and combining gemcitabine and GSI-IX prevented gemcitabine-induced enrichment of CSC-like population in in vitro models [584]. Similar to what was described in pancreatic cancer, Midkine-mediated upregulation of Notch1, responsible for Notch2 upregulation, was also reported as a resistance mechanism for biliary tract cancer [585]. In NSCLC, chronic exposure to gemcitabine drastically upregulates Notch3 expression by tumor cells, whereas the addition of DAPT sensitized cell lines to the pyrimidine analogue, affecting pro- and anti-apoptotic protein expression patterns [586]. At the same time, in SCLC, where the oncogenic impact of Notch signaling is less clear, a combined RBP-jκ/MAML3 inhibition reduced SCLC sensitivity to gemcitabine, which is possibly explainable by the inhibition of proliferation under RBP-jκ/MAML3 suppression [587]. In cutaneous T-cell lymphoma cell lines gemcitabine treatment induced Notch1 and its target gene expression, and *Notch1* knockdown improved gemcitabine anti-proliferative efficiency [588].

Thus, the preclinical data on gemcitabine/Notch inhibitors combinations are abundant and descriptive, and they created the rationale for combinatory attempts in a clinical setting. The combination of RO4929097 and gemcitabine in patients with advanced solid tumors had acceptable tolerability, dose exhalation of RO was limited by its auto-induction, and 4 out of 18 patients had PR (nasopharyngeal carcinoma) and SD (pancreas, tracheal, and breast cancer). Of note, the median Notch3 levels in immunohistochemistry staining were higher in individuals who received less than four cycles of drug combination (NCT01145456) [392]. The combination of MK-0752 with gemcitabine for the treatment of metastatic pancreatic cancer had a favorable pharmacokinetic and safety profile, and in a preliminary setting of a phase I trial, 13 of 19 patients had SD and one had a PR (NCT01098344); however, the matched pre/post-treatment tumor samples were characterized by a surprisingly low basal expression of HES1, contrasting with effective inhibition of Notch signaling in the hair follicles of these patients [397]. Despite the promising preclinical results on targeting Notch2 and Notch3 to overcome gemcitabine resistance, the addition of anti-Notch2/3 antibody tarextumab to gemcitabine and nab-paclitaxel worsened the PFS in metastatic pancreatic cancer patients, and contrasting with the precedent correlations, patients’ responses did not correlate with Notch3 expression [208]. Two trials evaluating the combinations of gemcitabine (±protein-bound paclitaxel) and anti-DLL4 demcizumab for the treatment of advanced pancreatic cancer have been registered (NCT01189929 and NCT02289898). The second one evidenced that demcizumab did not improve the PFS compared with placebo and the standard-of-care treatment (Table 1 and Table 2).

#### 5.5.3. Purine Antagonists

##### Fludarabine

Fludarabine is a purine analog inhibiting ribonucleotide reductase and DNA polymerase and thus interferes with DNA synthesis. Considering its preferential distribution to blood cells, it is widely used for the treatment of hematological malignancies, being highly effective in chronic lymphoblastic leukemia [589]. The addition of Notch inhibitors seemed to prevent stroma-mediated protection of leukemic cells from the cytotoxic action of fludarabine. Combination of anti-Notch antibodies (except anti-Notch3) or GSI-XII reverted bone marrow stroma-induced protection of chronic lymphoid leukemia (CLL) cells from the pro-apoptotic action of fludarabine associated with the upregulation of BCL-2 and NF-κB [381]. The combination of PF-03084014 with fludarabine had a synergic antileukemic effect in primary *Notch*-1-mutated CLL cells, even in the presence of protective stroma, which was mediated through upregulation of proapoptotic HRK, downregulation of MMP9, IL32, RAC2 related to invasion and chemotaxis, and overcoming fludarabine-induced activation of NF-κB signaling [590].

### 5.6. Notch and Glucocorticoids

Unlike other steroid hormone receptors driving the proliferation of hormone-dependent cancers, glucocorticoid receptor (GR) activation leads to growth arrest and apoptosis induction in lymphoid tissue that underlies the use of GR agonists for lymphoid cancer treatment and makes them one of the key components of multimodal treatment protocols of T-ALL [591]. In T-ALL, the presence of activating *Notch1* and inactivating *FBXW7* mutations was correlated with a good prednisone response and a better clinical outcome [592,593]. In preclinical studies, the addition of Notch inhibitors allowed leukemic cells to overcome resistance to glucocorticoids. Indeed, the inhibition of Notch1 signaling by a GSI Compound E suppressed *HES1*, restored glucocorticoid receptor (NR3C1) auto-upregulation, and induced apoptotic cell death through BCL-2L11 induction in a model of glucocorticoid-resistant T-ALL [190]. Another GSI PF-03084014 in combination with dexamethasone enhanced expression of glucocorticoid target genes (*RUNX2*, *PFKFB2*, *BCL-2L11*, *BMF*, and *TSC22D3*) and increased the cytotoxicity of dexamethasone in vitro and in vivo in a xenograft model of glucocorticoid-resistant T-ALL [191]. Additionally, OMP-52M51, an anti-Notch1 antibody targeting LNR and HD, potentiated dexamethasone effects in a murine xenograft model of advanced T-ALL derived from a prednisone-poor responder patient [209]. Moreover, bortezomib, a proteasome inhibitor that exerted its antileukemic action through *Notch1* transcriptional downregulation, was highly synergistic with dexamethasone in cellular and murine xenograft models of glucocorticoid-resistant T-ALL [382].

On the other hand, contrasting evidence has been obtained for glucocorticoid-sensitive cell lines. From one side, a synergic interaction between dexamethasone and Compound E was not evidenced for glucocorticoid-sensitive cell lines and B-cell driven tumors [190]. On the contrary, in another study, the combination of the same GSI Compound E and dexamethasone augmented the apoptotic effects of the latter one in some GSI-sensitive T-ALL cell lines [444]. However, in a clinical setting, the GSI crenigacestat (LY3039478) plus dexamethasone demonstrated limited clinical activity (which included, however, 1 CR that lasted for 10.51 months) and tolerability in adult patients with relapsed/refractory T-ALL/T-cell lymphoblastic lymphoma, and dexamethasone did not revert completely severe gastrointestinal adverse events that were registered in 16.7% of co-treated patients. The efficacy of Notch1 cleavage reduction varied from 66% in the group receiving 50 mg of crenigacestat to 87% in the group of 100–125 mg, but higher doses did not correspond with a better clinical outcome; moreover, the frequency of *Notch1* activating mutations in this study was quite low (NCT02518113) [396]. The results of one more completed phase 1 study evaluating the combination of another GSI BMS-906024 with dexamethasone in patients with T-ALL/T-cell lymphoblastic lymphoma (NCT01363817) are still to be published. One more study (NCT01236586) has been withdrawn.

Importantly, glucocorticoids may alleviate GSI-mediated intestinal toxicity. In line with this, dexamethasone treatment protected mice from GSI-induced intestinal goblet cell metaplasia through upregulation of cyclin D2, counteracting GSI-driven cell cycle arrest and upregulation of KLF4, a negative regulator of goblet cell differentiation [190]. Indeed, in one study, prednisone co-administration effectively reduced the gastrointestinal toxicity of crenigacestat (LY3039478) in patients with advanced or metastatic cancer, which allowed disease stabilization to be reached in 54.5% and 64.7% of individuals receiving different GSI dose regiments (NCT01695005) [395]. Despite this optimistic evidence, the previously described trial showed that dexamethasone did not help to overcome crenigacestat-related gastrointestinal toxicity that had led to treatment interruption in some responding patients (NCT02518113) [396].

### 5.7. Notch and Antitumor Enzyme-L-Asparginase

The antitumor enzyme L-asparginase, which catalyzes the deamidation of L-asparagine to L-aspartic acid and ammonia causing the nutritional stress and thus affecting the protein synthesis and cell growth of cancer cells, has been commonly included in chemotherapy regimens of acute lymphoblastic leukemia and other hematological malignancies [594]. It has not been widely tested in combination with Notch inhibitors; therefore, the existing evidence of the lack of apparent advantage is quite limited. The GSI Compound E augmented L-asparginase-induced apoptotic effects in GSI-sensitive T-ALL cell lines, whereas in GSI-resistant cells, its effect was antagonized by GSI through upregulation of the anti-apoptotic protein BCL-xL [444]. Interestingly, DAPT did not increase L-asparginase-induced apoptosis in GSI-resistant T-ALL cell lines [445].

## 6. Combining Radiotherapy and Notch Inhibition

**Figure 3:**
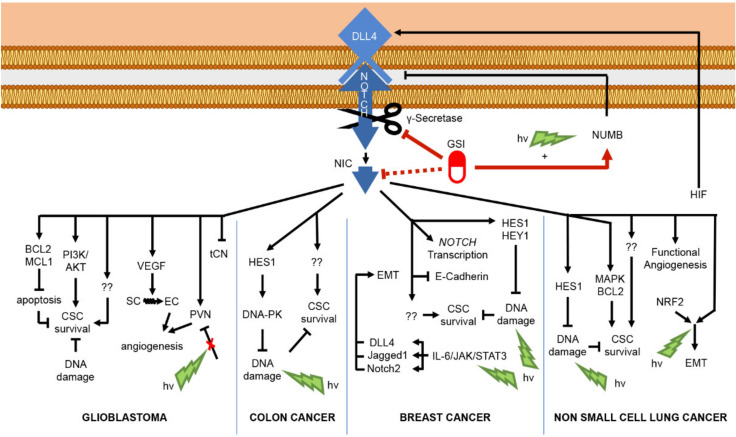
Schematic diagram of potential mechanisms mediating GSI effects on Notch-related cancer radioresistance. In glioblastoma, NIC sustains: CSC survival via the anti-apoptotic protein MCL1 (induced myeloid leukemia cell differentiation protein) and BCL2 (B-cell lymphoma 2) as well as via the pro-survival pathway PI3K/AKT; angiogenesis via VEGF (vascular endothelial growth factor) signaling that transforms stromal cells (SC) to endothelial cells (EC), and selecting the perivascular niche (PVN) cells. On the other hand, *Notch1* knockdown stimulates the formation of cell-communicating network (tCN) inside the tumor. In colon cancer, HES1 counteracts the accumulation of DNA damage by sustaining DNA-PK (DNA-dependent protein kinase) implicated in DNA repair following exposure to radiation (hν), therefore sustaining CSC survival. In breast cancer, the Notch target genes HES1 and HEY1 are implicated in contrasting DNA damage accumulation, while NIC also mediates the inhibition of oncosuppressor protein E-Cadherin and the increase in CSC survival via unspecified mechanisms following exposure to hν. Additionally, radiation stimulates the IL-6/JAK/STAT3 axis that upregulates DLL4, Jagged1, and Notch2 expression that promote EMT. In NSCLC, NIC sustains CSC survival via HES1, MAPK (mitogen-activated protein kinase), and BCL2 upregulation and is implicated in the functional angiogenesis. In addition, HIF is associated with DLL4-related Notch stimulation, and radiation stimulates the NRF2/Notch axis that enhances EMT. Finally, in NSCLC, the combined GSI-radiation therapy results in Numb-dependent downregulation of Notch. “??” represents other unknown mechanisms which may be involved in Notch-dependent CSC survival.

Notch inhibition seems to have an appealing potential in improving the therapeutic effects of radiotherapy (RT). Here, we give a short review of the available studies (summarized in Figure 3).

### 6.1. Glioblastoma Multiforme

Positive HEY1 and Notch1 expression was associated with shorter PFS and OS after chemo- and radiotherapy in patients with primary and recurrent glioblastoma, and Notch1 positive GSC had an increased potential to transform into endothelial cells possibly through Notch/VEGF crosstalk [126,127]. In a cell line of glioblastoma multiforme cell line, the combination of RT and a GSI DAPT significantly decreased cell proliferation in tumor explants as compared to single agents through affecting CSC pool in an endothelium-dependent way. The absence of endothelial cells reduced the combined therapy efficacy [125]. In a similar experiment, glioma stem cell survival was significantly reduced in vitro after combined RT and a GSI (DAPT or L685.458) and in vivo after combining RT and *Notch1*/*2* shRNA knockdown, as compared to individual treatments. Again, the CSC population was responsible for increased sensitivity to RT, and Notch inhibition prevented pro-survival PI3K/AKT and MCL-1 upregulation [595]. Combining a GSI RO4929097 with TMZ and RT reduced tumor growth and prolonged survival of mice in a xenograft model, once more affecting cells with stem-like behavior [189]. Additionally, Notch signaling activation contributed to the protection of malignant stromal cells induced by glioma progenitors against radiation in the orthotopic glioma nude mouse model, whereas addition of GSI improved their radiosensitivity and reverted BCL-2 and pAKT upregulation induced by radiation [596]. The clinical trial assessing the addition of RO-4929097 to TMZ demonstrated the relative safety of this approach [390] (NCT01119599). Despite the decrease in CSC pool and modulation of Notch signaling, some patients experienced recurrence associated with the upregulation of mesenchymal genes and VEGF-dependent angiogenesis (for details see “Temozolomide” chapter and Table 1). To note, Notch signaling may act both as oncogene and tumor suppressor in glioma development, which raises additional contradictions for combined approaches [130]. A recent study demonstrated that both perivascular niche cells and network-forming cells of glioma could be highly resistant to radio- and chemotherapy, but these two populations were inversely regulated by Notch1. In particular, *Notch1* knockdown depleted perivascular niche pool and at the same time stimulated the formation of tumor microtubules responsible for the formation of communicating network inside the tumor [597]. Therefore, concomitant Notch inhibition may act as a double-edged sword eradicating one radiation-induced resistance mechanism while favoring another one. This consideration could be a possible explanation of tumor recurrence in patients who received such combined treatments.

### 6.2. Colorectal Cancer

In colon cancer, the combination of ionizing radiation and an anti-DLL4 antibody or a GSI dibenzazepine impaired tumor growth by promoting nonfunctional tumor angiogenesis and tumor necrosis [598]. However, a GSI DAPT or *Notch1* knockdown improved radiation sensitivity of colon cancer cells directly by affecting the Notch1/HES1 axis, leading to enhanced irradiation-induced DNA damage and attenuated radiation-triggered DNA-PK activity [599].

### 6.3. Breast Cancer

In mammary tumor cells, radiation stimulated EMT through the IL-6/JAK/STAT3 signaling axis, which upregulated *JAG1*, *DLL4*, and *Notch2* transcripts, and GSI addition effectively attenuated migration and mesenchymal markers expression in the irradiated cells [600]. Moreover, in breast cancer models, GSI helped to reduce CSC pool enrichment by RT, limited the functional consequences of Notch pathway upregulation by RT, and affected migration and invasion of cancer cells by a mechanism based on decreased expression of Notch target genes *HES1* and *HEY1* induced by ionizing radiation [601,602]. One CT assessing the combination of RO4929097 and whole-brain radiation therapy for treating patients with breast cancer brain metastases was registered (NCT01217411); however, due to a small number of patients enrolled (*n* = 5) and discontinuation of RO4929097 development, the published results are not available for analysis.

### 6.4. NSCLC

High expression of *Notch1*, *JAG2*, and *HES1* mRNA in resected tissue samples of NSCLC patients that did not receive neoadjuvant therapy correlated with worse DFS, and xenografts with high Notch activity grew faster, had hypoxic features, and possessed notable radioresistance [603]. Notch inhibitor BMS-906024 was synergistic in combination with chemoradiation and effectively delayed spheroid growth in NSCLC cell lines [400]. Increased cell death of NSCLC cell lines after addition of GSI I and GSI XX to RT was related to Notch1 and Notch3 inhibition mediating MAPK and BCL2 downregulation not accompanied with AKT activation and was further confirmed with additive-to-synergistic values of CI [604]. A GSI MW167 enhanced the inhibiting effects of X-ray on the proliferation, invasion, and migration of lung cancer cells affecting the Numb/Notch1/HES1 axis [605]. A triple GSI, HIF-1 inhibitor, and RT combination had a synergistic antitumor effect in vitro, since HIF-1 could upregulate DLL4 expression that in its turn activated Notch3, whereas the addition of HIF-1 inhibitor prevented the radiation-induced Notch3 activation [606]. Another mechanism of enhanced radiosensitivity was related to the NRF2/Notch1 axis inhibition and resulted in reduced EMT in irradiated cells [607]. It is worth mentioning that combining Notch inhibitors with radiotherapy had quite contrasting consequences for airway epithelium regeneration in experimental works. Radiation damages the DNA of progenitor cells, and Notch signaling inactivation increases the phosphorylation of ATM and CHK2 and alters DNA damage response, but it could result both in decreased and improved renewal capacity of basal stem cells in different conditions [608,609].

## 7. Conclusions and Future Perspectives

Several approaches intercepting different steps of the Notch pathway activation have proven their anti-tumor effects in pre-clinical and early clinical testing as single agents and as a part of combination therapy. However, their translation to clinical studies has revealed some associated problems such as the lack of expected efficacy and severe adverse effects.

The preclinical evidence of CSC pool and tumor angiogenesis reduction after associating Notch inhibitors and temozolomide was confirmed in an early-stage CT combining RO4929097 and TMZ; however, some tumors relapsed due to the upregulation of mesenchymal genes and VEGF signaling.

In terms of platinum-based drugs, Notch blockage showed synergistic activity with cisplatin by affecting the CSC pool, MDR transporters, and autophagy in several preclinical studies. However, the available clinical data showed no benefit of demcizumab together with carboplatin/pemetrexed compared to placebo in NSCLC, possibly due to the upregulation of other proangiogenic mechanisms. Similarly, the additive combinations between GSI and carboplatin in SCLC and the apparent efficiency of Rova-T as a single approach in NET in vitro and in vivo experimentations were not extrapolated to CT, where Rova-T gave no benefit to the first-line platinum/etoposide-based chemotherapy in either of sequential regimens and was less effective than the second-line drug topotecan. Not even the anti-Notch2/3 antibody tarextumab showed any benefit in CT in this cancer. The controversial role of Notch signaling in SCLC together with the paradoxical prevalence of clinical over experimental data encourages considering specular Notch-activating approaches for this cancer. Interestingly, the addition of Notch-targeting agents sensitized experimental models of ovarian cancer and colon cancer to the treatments with cisplatin and with platinum derivatives, respectively, by affecting the selection and the survival of the CSC population. However, all these findings need clinical confirmation.

In relation to microtubule-targeting drugs, a positive preclinical evidence of combinations between vincristine and GSI in T-ALL is available; however, it may not be completely dependent on Notch signaling. In a preclinical setting, Notch inhibition sensitized various types of cancer to taxanes through affecting CSC, EMT, and pro-survival pathways, enhancing the taxane-induced mitotic arrest, and possibly involving the crosstalk between tubulin dynamics and Notch nuclear localization. In ovarian cancer, these benefits were translated into a modest clinical efficacy linked to a promising opportunity to affect DLL4-driven tumor angiogenesis after the potential of anti-VEGF treatment had expired. In breast cancer, CT confirmed CSC pool reduction in concomitant or sequential combinations with paclitaxel; however, the clinical benefit was limited. One optimistic detail revealed from the treatment-naïve patients should be mentioned, since some positive pathological OR was obtained after using the GSI RO4929097 in combination with neoadjuvant paclitaxel and carboplatin in operable TNBC. In pancreatic cancer, the controversial preclinical evidence on the role of Notch paralogs could explain the lack of tarextumab addition benefit in combination with paclitaxel and gemcitabine.

In terms of anthracyclines, preclinical studies showed no benefit/antagonism in combination with GSI in T-ALL. In solid tumors, GSI and mAb counteracted the Notch-associated drug resistance mechanisms with some differential response in relation to p53 status. Indeed, the preclinical evidence on combining another DNA-damaging agent etoposide with Notch inhibitors was conceptually similar, since these drug associations were antagonistic in T-ALL and gave no benefit in some solid tumors such as colon cancer and glioma, whereas in breast cancer, the response depended on p53 status. Since Notch signaling may be involved in DNA repair regulation, its interference with anthracyclines and topoisomerase II inhibitors’ modes of action should be considered. Despite this, the preclinical data on the second class of topoisomerase I inhibitors (irinotecan and topotecan) in combination with Notch inhibitors seems to be promising. However, it is mostly limited to in vitro and in vivo models of colon cancer. The benefit of these approaches will be cleared up when the ongoing FOLFIRI/Notch inhibitors-combining trials are completed.

In addition, the preclinical evidence on combining folic acid antagonists and Notch inhibitors is quite limited and has not been linked to the specific mechanisms of antifolate resistance so far.

There is generally positive preclinical data for colon cancer Notch-inhibition-mediated sensitization to 5-FU through different mechanisms including the specific ones such as TS modulation. However, when not a single 5-FU, but all components of FOLFIRI protocol were applied to colon cancer cells in vitro, the sensitivity to Notch inhibition was lowered due to the strong antiproliferative effect that could explain the lacked benefit of ABT-165 (anti-DLL4) over anti-VEGF in the only completed so far CT.

Despite that the preclinical potential of targeting Notch pathway elements together with gemcitabine is mostly mediated through Notch2/3, the addition of anti-Notch2/3 blocking antibodies to gemcitabine in CT worsened the PFS, and patients’ response did not correlate with Notch3 expression. In addition, another completed trial with GSI MK-0752 revealed a low basal Notch pathway activation in the pre-treatment samples associated with paradoxically effective inhibition of Notch signaling in normal tissues, which explained the low susceptibility of tumors to Notch blockade and created the physiological background for GSI-related adverse effects.

A clear mechanism of GSI-mediated glucocorticoid sensitization in T-ALL has been suggested, whereas the effects of concomitant Notch inhibition and GR stimulation in glucocorticoid-sensitive cell lines were quite contrasting. In particular, a clinical study revealed modest efficacy of GSI combination with dexamethasone, even though the poor outcome of the trial could be due to the low frequency of *Notch*-activating mutations in the involved patients [551]. The attempts to overcome the gastrointestinal toxicity of GSI with glucocorticoid premedication were less optimistic than preclinical data yet not completely delusive.

Even if Notch signaling orchestrates some general tumor resistance mechanisms such as CSC, EMT, tumor angiogenesis, drug efflux transporters, pro-survival pathways, and tumor-stroma interactions, the impact of each member of the pathway is quite context-dependent and should not be excessively generalized. Indeed, different Notch paralogs may play different roles within the same tumor as in pancreatic cancer, and the impact of Notch signaling may be controversial as in SCLC and glioma. Moreover, different ligands may provide a variable Notch signaling induction. Knowledge of the importance of a particular Notch-mediated functional read-out for a distinct type of tumor may become a rationale for a better implication of Notch inhibitors in CT and could be useful for further understanding of the results of the ongoing and completed ones.

To date, except for the GSIs AL101 and nirogacestat, recently granted with the Orphan Drug Designation and Fast Track Designation by the FDA for the treatment of Notch-mutant ACC and desmoid tumors, respectively, no other Notch-targeting therapy has been clinically approved. An important issue of the therapeutic modulation of Notch signaling is the on-target side effects on vulnerable healthy tissues. One way to solve this problem may be fulfilled through designing Notch-based therapeutic approaches with selective delivery to tumor cells to limit systemic distribution and undesired modulation of the pathway in the other tissues. In this case, the development of Notch-inhibitor agents linked to antibodies against specific antigens highly expressed on the membrane of distinct cancer cells would be a fascinating solution. Of note, an advanced delivery might contribute to the efficiency of Notch antagonists against the CNS tumors as the available molecules might have a limited possibility to cross the BBB. Another specificity-aiming approach may implicate the Notch-specific education of the immune system that is currently being investigated in CT with CAR-T cells modified to recognize DLL3-positive cells of SCLC.

Additionally, the Notch-mediated interaction of tumor cells with the stroma and the immune system should be considered, and patient-derived tumor organoids with isolated microenvironment could be a suitable experimental model for it.

Particular attention should be paid to VEGFR, MAPK, PI3K/AKT, and other pathways cross-talking with Notch since their compensatory activation often underlies the resistance to Notch inhibition. Interestingly, the molecules influencing the epigenetic machinery and NTC provide a fruitful field for future discoveries. Among them, a high expectation is addressed to the potential clinical applicability of the orally active small molecules CB-103 and NADI-351 directly interfering with the NTC formation and function, which have demonstrated outstanding pre-clinical safety, efficacy, and pharmacokinetics.

In the last decade, several natural molecules and their derivatives, including curcumin, butein, quercetin, and withaferin A, have proven their ability to modulate the Notch signaling cascade and to affect the proliferation of multiple types of cancer cells both in vitro and in vivo. However, the molecular mechanisms underlying their anti-Notch and anti-growth effects remain controversial, whereas the major existing evidence on them is still based on preclinical studies.

Notably, the delusive results of some CT may be explained by the low quality of molecular selection of patients obliging to apply Notch inhibitors where they are not really needed. However, the tough reality of advanced stage cancer often implies the experimental approaches as the last weapon rather than a carefully selected target. Indeed, most of the available studies have been conducted in severely pre-treated patients with the resistance to conventional therapy. Anyway, the detection of gain-or-loss of function mutations of the principal Notch signaling elements together with the immunohistochemical analysis of the ligands and the activated form of the different Notch receptors expression in pre-treatment tumor samples could advance the choice of a more suitable treatment for distinct patients and should be included in the eligibility criteria of CTs. Finally, the sequential combinations between Notch inhibitors and chemotherapeutic agents should be evaluated initially in a preclinical setting, as their functional outcome may differ. Moreover, the preclinical evidence of antagonism between Notch inhibitors and several classes of drugs should not really preclude their implication in treatment protocols but rather create a rational background for separating these agents in space and time, as it is realized in some T-ALL treatment protocols where methotrexate and cytarabine may be administered at different days due to a possible antagonism between them [610].

## Figures and Tables

**Table 1:** Clinical trials with GSI in combination with chemotherapeutic drugs registered at clinicaltrials.gov.

Agent	CT Identifier	Phase	Cancer Type	Drug Combination	Results (as of 1 August 2021)
RO4929097/R4733	NCT01238133	I	Operable Triple-Negative Breast Cancer	Neoadjuvant with Paclitaxel+Carboplatin	*n* = 14, pCR in 36% of patients, 4 out of 5 patients of higher dose group required dose reduction due to toxicity (neutropenia, thrombocytopenia, hypertension); no paired pre/post-treatment biopsies [391]
	NCT01236586	I	Relapsed/Refractory Solid or CNS tumors, Lymphoma, or T-Cell Leukemia	Dexamethasone	No, withdrawn
	NCT01196416	I/II	Recurrent or Metastatic Melanoma	Cisplatin, Vinblastine, and Temozolomide	*n* = 14, PR or SD in 8 out of 14 patients which correlated with reduced Notch cleavage in 4 out of 5 analyzed cases of objective response. Adverse effects: leukopenia, thrombocytopenia, elevated transaminases, electrolyte disturbances, hyperglycemia, nausea, vomiting (available at clinicaltrials.gov, accessed on 30 July 2021)
	NCT01119599	I	Malignant Glioma	Temozolomide+radiotherapy	*n* = 21, MTD was reached (20 mg), no treatment discontinued due to toxicity, generally well-tolerated. PFS 13 months, OS 21 months, better survival correlated with N1IC reduction in post-treatment samples. Reduction of tumor blood perfusion on MRI, significant decrease in N1IC-expressing microvessels without affecting overall microvascular density. The drug had variable BBB penetration with higher concentrations achieved in BBB-disrupted samples. DLL1, DLL3, Jagged2, and HES5 but not HES1 downregulated in post-treatment samples of BBB-disrupted tumors. RO decreased CD133+ CSC pool [390]
	NCT01145456	I	Advanced Solid Tumors	Gemcitabine	*n* = 18, recommended RO dose for combination with gemcitabine: 30 mg, autoinduction at higher doses, PR in 1 patient (nasopharyngeal carcinoma), SD in 3 patients (pancreas, tracheal, and breast cancer) (*n* = 18). Adverse effects: elevated transaminases, skin rush, neutropenia. Notch3 levels at IHC were lower in patients who received more than 4 cycles of RO, higher levels of Notch3 in tumor tissue were associated with resistance to RO4929097+gemcitabine [392]
	NCT01158274	I	Refractory Solid Tumors	Capecitabine	*n* = 30, MTD was not reached, RO autoinduction at high doses, PR in 2 patients (fluoropyrimidine-refractory colon cancer and cervical cancer). Adverse effects: nausea, vomiting, hypophosphatemia, diarrhea [393]
	NCT01192763	I	Pancreatic Cancer	Various neoadjuvant	No, terminated
Nirogacestat/PF-03084014	NCT01876251	I	Advanced Breast Cancer	Docetaxel	*n* = 29, MTD 100 mg twice daily, PR in 4 and SD in 9 out of 25 patients, median PSF 4.1 months in the expansion cohort. Adverse effects: neutropenia, fatigue, nausea, leukopenia, diarrhea, alopecia, anemia, vomiting. *Notch1* and *Notch2* RNA in serum decreased on the 2nd day after treatment and increased on the 8th day compared with the baseline. *Notch4* RNA in serum decreased on the 8th day [394]
	NCT02109445	I/II	Metastatic Pancreatic Adenocarcinoma	Gemcitabine and Nab-Paclitaxel	*n* = 3, phase II was not performed, only some pharmacokinetic data posted (available at clinicaltrials.gov, accessed on 31 July 2021)
LY3039478/JSMD194	NCT02784795	I	Advanced or Metastatic Solid Tumors	Cisplatin/Gemcitabine, or Gemcitabine/Carboplatin, or Taladegib, or LY3023414, or Abemaciclib	No
	NCT01695005	I	Advanced or Metastatic Solid Tumors	Prednisone	*n* = 28, combination aimed to mitigate GSI intestinal toxicity. SD in 54.5% and 64.7% of patients receiving 75 to 150 mg escalating doses of LY TIW (F1) or BIW (F2), respectively. DLT: increased serum amylase, fatigue, hypophosphatemia, maculopapular rush. No DLT in combination with prednisone, GI toxicity less frequent than in no-prednisone groups. In matched pre- and post-treatment tumor samples (*n* = 10) positive for Notch1 at baseline, 5 were negative for Notch1 post-treatment (2 patients had SD), 2 biopsies remained positive (both SD), and 3 biopsies were not evaluable [395]
	NCT03502577	I	Multiple Myeloma	BCMA-specific CAR T followed with fludarabine and cyclophosphamide	No
	NCT01363817	I	T-ALL or T-LBL	Dexamethasone	No
	NCT02518113	Ib/II	T-ALL/T-LBL	Dexamethasone	*n* = 36, 1 patient had CR that lasted 10.51 months, 16.7% (*n* = 6) had SD, 33.3% (*n* = 12) had PD. 47.2% (*n* = 17) were not evaluable, median PSF was 1.18 months. MTD: 75 mg LY + 24 mg dexamethasone daily on 1–5 days of treatment. Adverse reactions in 77.8% of patients. Dexamethasone did not revert severe GI adverse events that were registered in 16.7% of patients. DLT: GI hemorrhage, nausea, vomiting, diarrhea. The efficacy of Notch1 cleavage reduction varied from 66% in the group receiving 50 mg of crenigacestat to 87% in the group of 100–125 mg, but higher doses did not correspond to a better clinical outcome [396]
AL101/BMS-906024	NCT01653470	I	Advanced/Metastatic Solid Tumors	Paclitaxel or FOLFIRI or Paclitaxel with and without Carboplatin	No
MK-0752	NCT01098344	I	Inoperable Stage III/IV Pancreatic Cancer	Gemcitabine	*n* = 44, 13 patients had SD and 1 patient had PR among 19 patients appropriate for tumor response analysis, median time to disease progression was 169 days, median time of overall survival was 246 days. Adverse effects: 55% patients—nausea, 55%—vomiting, 48%—diarrhea, 40.5%—thrombocytopenia, 41%—anemia, 33%—anorexia, 31% -fatigue, 29%—neutropenia. Significant inhibition of Notch signaling in hair follicles was observed in 25/29 patients, no dose-dependent relationship, HES1 expression was evaluated in 20 matched pre/post treatment tumor samples, basal HES1 expression was low, HES1 expression post-treatment was lower in 2 out of 20 biopsy pairs [397]
	NCT00645333	I/II	Advanced/Metastatic Breast Cancer	Docetaxel and Pegfilgrastim	*n* = 30, of 24 participants evaluable for response, 11 PR, 9 SD, and 3 PD were observed. MTD of MK in combination with docetaxel was 600 mg, 5 cases of DLT, serious adverse effects in 55.3% of patients, adverse effects: 66.67%—fatigue, 50.00%—nausea, 33.33%—diarrhea/hyperglycemia/nail changes, decrease in CD44+/CD24–, ALDH(+) cells in tumors of patients undergoing serial biopsies (3/5) after several cycles of treatment [398]

**Table 2:** Clinical trials with monoclonal antibodies against Notch receptors and ligands in combination with chemotherapeutic drugs registered at clinicaltrials.gov.

Agent	CT Identifier	Phase/Type	Cancer Type	Drug Combination	Results Description (as of 1 August 2021)
Tarextumab (OMP-59R5)	NCT01859741	I/II	Stage IV SCLC	Etoposide and Cisplatin/Carboplatin	Phase I (*n* = 3, 5, 6 in different dose regimens): MTD was not reached, the recommended phase II determined as 15 mg/kg every 21-day cycle. PR or SD in 80–100% of participants in different OMP dose regimens. OMP-59R5 (15 mg/kg) + ETO + CIS: 83.3% PR, 16.7% PD; OMP-59R5 + ETO + CARB: 66.7% PR, 16.7% SD, 16.7% PD. Phase II (*n* = 72 in placebo + CIS/CARB, *n* = 73 in OMP-59R5 + ETO + CIS/CARB): during 1 year observation period, the frequency of disease progression or death in the group of placebo and OMP + ETO was 77.8% and 69.9%, respectively. The frequency of CR was 2.8% and 1.4%, PR 68.1% and 67.1%, SD—13.9% and 12.3% in the groups of placebo and OMP-59R5 + ETO, respectively. The frequency of serious adverse effects was 42.65% (placebo) and 53.62% (OMP-59R5 + ETO), among them: febrile neutropenia, diarrhea, pancytopenia, and cardiac disorders (available at clinicaltrials.gov, accessed on 29 July 2021)
	NCT01647828	I/II	Untreated Stage IV Pancreatic Cancer	Gemcitabine and Nab-Paclitaxel	(*n* = 177) Median OS was 6.4 months in tarextumab group vs. 7.9 months in the placebo group (HR 1.34, *p* = 0.0985). No difference in OS in the *Notch3* gene expression subgroups. PFS in the tarextumab-treated group (3.7 months) was significantly shorter compared with placebo (5.5 months). No difference in ORR. Adverse effects in tarextumab group: diarrhea (72%), fatigue (52%), thrombocytopenia (49%), nausea (41%) [208]
Brontictuzumab (OMP-52M51)	NCT03031691	I	Metastatic Colorectal Cancer	Trifluridine/Tipiracil	No
Demcizumab (OMP-21M18)	NCT01952249	Ib/II	Platinum Resistant Ovarian Cancer	Paclitaxel	(*n* = 19), MTD not reached, established dose 3.5 mg/kg, overall response rate 21%, 79% of patients had PD, clinical benefit rate was 42% (PR in 4 patients (21%) and SD in 4 patients (21%), no DLT. Common adverse effects: 68%—diarrhea, 38%—fatigue, 53% peripheral edema, 53% nausea, 16% pulmonary hypertension [225]
	NCT01189968	I	Untreated Metastatic Non-Squamous NSCLC	Carboplatin and Pemetrexed	(*n* = 46), truncated dose regimen and phase II dose 5 mg/kg weekly were recommended. 20 out of 40 (50%) evaluable patients had OR. CR in 1 patient (3%), PR in 19 patients (48%), SD in 15 patients (38%), PD in 5 patients (13%). Clinical benefit rate was 88%/PFS and OS in truncated regimen groups were 5.8 and 11.5 months, respectively. Adverse effects: 80%—fatigue, 67%—vomiting, 54%—constipation, 48% - anemia, 48% - dyspnea, 46% - hypertension, 41% - diarrhea, 37% - headache, 35% - thrombocytopenia or neutropenia. Compared with the baseline, blood expression levels of *Notch1, Notch2, MAML2,* and *MAML3* decreased, and *LEF1* and *SFRP2* (regulators of blood vessel branching) were increased [226]
	NCT01189929	I	Locally Advanced or Metastatic Pancreatic Cancer	Gemcitabine ± Abraxane	No
	NCT02289898	II	Metastatic Pancreatic Ductal Adenocarcinoma	Gemcitabine, Abraxane	(*n* = 204), demcizumab did not improve PFS compared to placebo (HR 0.93, *p* = 0.7158, Kaplan-Meier-based estimation). Frequent adverse effects in demcizumab treatment arms: anemia, diarrhea, vomiting, fatigue, peripheral edema (available at clinicaltrials.gov, accessed on 1 August 2021)
	NCT02259582	II	Non-Squamous NSCLC	Carboplatin and Pemetrexed	(*n* = 82), PR and SD frequency in placebo and two demcizumab arms of trial, respectively: 52% and 40%, 35.7% and 50.0%, 20.7% and 51.7%. Frequency of serious adverse events: 24.0% in placebo group and 39.29 and 51.72% in two demcizumab arms. Common adverse effects in two demcizumab treatment arms: nausea (64.29% and 48.28%), fatigue (57.14% and 41.38%), vomiting (28.57% and 37.93%), diarrhea (21.43% and 44.83%), decreased appetite (39.29% and 3.03%), hypertension (50.00% and 41.38%), elevated BNP (28.57% and 20.69%) (available at clinicaltrials.gov, accessed on 29 July 2021)
	NCT01189942	I	Metastatic Colorectal Cancer	FOLFIRI	No
Navixizumab (OMP-305B83)	NCT03030287	Ib	Ovarian, Peritoneal or Fallopian Tube Cancer	Paclitaxel	No
	NCT03035253	I	Metastatic Colorectal Cancer	FOLFIRI or FOLFOX	No
ABT-165	NCT03368859	II	Metastatic Colorectal Cancer Previously Treated with Fluoropyrimidine, Oxaliplatin and Bevacizumab	FOLFIRI	(*n* = 70) PFS was 3.78 months and 7.36 months, and ORR was 5.6% and 14.7% in ABT-165 + FOLFIRI and bevacizumab + FOLFIRI groups, respectively. All-cause mortality and frequency of serious adverse events was higher in ABT-165 group compared to bevacizumab (35.29% vs. 18.75% and 50.00% vs. 25.00%, respectively). Common adverse effects in ABT-165 group: 52.94%—diarrhea, 52.94%—nausea, 41.18%—neutropenia, 29.41%—hypertension (available at clinicaltrials.gov, accessed on 27 July 2021)
	NCT01946074	I	Solid Tumors	Alone or FOLFIRI or Paclitaxel with and without ABBV-181	No
Rovalpituzumab tesirine (Rova-T)	NCT02819999	I	Extensive Stage SCLC	Cisplatin and Etoposide	(*n* = 26), 4 cohorts evaluating Rova-T alone and in different sequential combinations of Rova-T and cisplatin + etoposide (CE). Combination of Rova-T and CE did not add benefit to median OS and ORR of CE alone. Median OS in Rova-T + CE was 10.3 months, median PFS was 5.2 months, ORR was 50% (in other studies, ES alone produced ORR 60–70%, and median OS around 10 months). Cohort of lower dose of Rova-T + CE showed lower frequency of Rova-T-related adverse events such as pleural effusion (0 vs. 33%), pericardial effusion (0 vs. 17%), ascites (0 vs. 8%), peripheral edema (36% vs. 42%), generalized edema (0 vs. 8%), pneumonia (7% vs. 25%), and hypoalbuminemia (0 vs. 17%) [246]
	NCT03033511	III	Advanced SCLC	Rova-T or placebo following platinum-based chemotherapy (+etoposide or irinotecan) 3–9 weeks after achieving CR/PR/SD	(*n* = 748), no benefit for OS in both low- and high-DLL3-expressing subsets, PFS better in Rova-T group (4.0 vs. 1.4 months in Rova-T group and placebo, respectively). Rova-T-associated adverse effects: 27%—pleural effusion, 27%—decreased appetite, 26%—peripheral edema, 25%—photosensitivity reaction, 25%—fatigue, 22%—nausea, 21%—dyspnea [241]
	NCT03061812	III	Advanced or Metastatic DLL3-high SCLC	Rova-T or topotecan in patients with first disease progression following platinum-based chemotherapy	(*n* = 444), Rova-T exhibited lower OS (6.3 months) compared to topotecan (8.6 months) and lower PFS (3.0 and 4.3 months in Rova-T and topotecan groups, respectively). ORR was 15% in the Rova-T arm and 21% in the topotecan arm. One CR in the Rova-T group, no CR in the topotecan group. 14% of PR in the Rova-T arm, 21% of PR in the topotecan arm. Rova-T-associated adverse events: pleural effusion (29%), decreased appetite (25%), dyspnea (25%), fatigue (25%), nausea (23%), and pericardial effusion (20%) [240]

## Supplementary Materials

The following are available online at https://www.mdpi.com/article/10.3390/cancers13205106/s1, Table S1: Principal Notch inhibiting strategies, Table S2: Clinical trials with GSI as a monotherapy registered at clinicaltrials.gov, accessed on 15 July 2021, Table S3: Clinical trials with monoclonal antibodies against Notch receptors and ligands as a monotherapy registered at clinicaltrials.gov, accessed on 19 July 2021.

## Abbreviations

5-FU	5-Fluorouracil
ACC	Adenoid Cystic Carcinoma
ACL	Lung Adenocarcinoma
ADAM	A Disintegrin and Metalloproteinase
AIF	Apoptosis-Inducing Factor
ALDH	Aldehyde Dehydrogenase
ALL	Acute Lymphoblastic Leukemia
AML	Acute Myeloid Leukemia
ANK	Ankyrin Repeats
APP	Amyloid Precursor Proteins
ARA-C	Cytarabine
ASCL1	Achaete-Scute Homolog 1
ATP	Adenosine Triphosphate
B-ALL	B-cell Acute Lymphoblastic Leukemia
BBB	Blood−Brain Barrier
BBC3	BCL-Binding Component 3
BCL	B-cell lymphoma
BMF	BCL2-Modifying Factor
BRD4	Bromodomain-containing protein 4
BTK	Bruton Tyrosine Kinase
CBF1	C-repeat/DRE-Binding Factor 1
CD	Cluster of Differentiation
CI	Combination Index
CLL	Chronic Lymphocytic Leukemia
CNS	Central Nervous System
CRC	Colorectal Cancer
CR	Complete Response
CSC	Cancer Stem Cell
CSL	CBF-1/RBP-Jκ/Suppressor of Hairless and Lag-1
CT	Clinical Trial
CtBP	C-Terminal Binding Protein
CtIP	CtBP Interacting Protein
DAC	5-aza-2′deoxycytidine
DGC	Diffuse Gastric Cancer
DHFR	Dihydrofolate Reductase
DLK	Delta-like Homolog
DLL	Delta-like Ligand
DN	Double Negative
DNMT	DNA Methyltransferase
DSL	Delta/Serrate/Lag2 ligands
EAC	Esophageal Adenocarcinoma
EC	Endothelial Cells
EGF	Epidermal Growth Factor
EGFR	Epidermal Growth Factor Receptor
EMT	Epithelial-Mesenchymal Transition
ER	Endoplasmic Reticulum
ERα+	Estrogen Receptor α-Positive
ERK	Extracellular Signal-Regulated Kinase
ESCC	Esophagus Squamous Cell Carcinoma
EZH2	Enhancer of Zeste Homolog 2
FOLFIRI	Folinic Acid/5-FU/Irinotecan
FBXW7	F-Box and WD Repeat Domain Containing 7
FDA	Food and Drug Administration
FGFR	Fibroblast Growth Factor Receptor
GBM	Glioblastoma Multiforme
GCT	Gastric Carcinoid Tumor
GI	Gastrointestinal
GR	Glucocorticoid Receptor
GS	Gamma-Secretase Complex
GSC	Glioma Stem-Like Cells
GSI	Gamma-Secretase Inhibitor
GSK-3	Glycogen Synthase Kinase-3
H	Histone
HAT	Histone Acetyltransferase
HCC	Hepatocellular Carcinoma
HD	Heterodimerization Domain
HDAC	Histone Deacetylase
HER	Human Epidermal Growth Factor Receptor
HES	Hairy/Enhancer of Split
HIF	Hypoxia Inducible Factor
HMT	Histone Methyltransferase
HNSCC	Head and Neck Squamous Cell Carcinoma
HNK	Honokiol
HPV	Human Papillomavirus
HSC	Hematopoietic Stem Cells
IC50	Half-Inhibitory Concentration
IGF1R	Insulin-like Growth Factor 1
IKK	Inhibitor of Nuclear Factor-κB (IκB) Kinase
IL	Interleukin
IMR-1	Inhibitor of Mastermind Recruitment-1
IXN	Isoxanthohumol
JMJD3	Jumonji Domain-Containing Protein 3
KDM	Lysine Demethylase
KMT	Lysine Methyltransferase
LCNEC	Large-Cell Neuroendocrine Carcinoma
LIC	Leukemia-Initiating Cells
LiCl	Lithium Chloride
LNR	Lin12-Notch Repeats
LSC	Leukemia Stem Cells
LSCC	Lung Squamous Cell Carcinoma
LSD1	Lysine Demethylase 1
mAb	Monoclonal Antibody
MAGP	Microfibril-Associated Glycoprotein
MAM	Mastermind
MAML	Mastermind-like
MAPK	Mitogen-Activated Protein Kinase
MCL1	Myeloid Cell Leukemia Sequence 1
MDR	Multidrug Resistance
miR	MicroRNA
MM	Multiple Myeloma
MMP	Matrix Metallopeptidase
MRI	Magnetic Resonance Imaging
MRP1	Multidrug-Resistance-Associated Protein-1
MTC	Medullary Thyroid Cancer
mTOR	Mammalian Target of Rapamycin
NAC	N-Acetylcysteine
N1IC	Notch1 Intracellular Domain
N2IC	Notch2 Intracellular Domain
N3IC	Notch3 Intracellular Domain
N4IC	Notch4 Intracellular Domain
NEC	Notch Extracellular Domain
NET	Neuroendocrine Tumor
NEXT	Notch Extracellular Truncation
NF-κB	Nuclear Factor κB
NHD	Notch Heterodimerization Domain
NIC	Notch Intracellular Domain
NR2F6	Nuclear Receptor Subfamily Group F Member 6
NRF2	Nuclear Factor Erythroid 2-Related Factor 2
NRR	Negative Regulatory Region
NSC	Neural Stem Cells
NSCLC	Non-Small-Cell Lung Cancer
NTC	Notch Transcription Complex
NTM	Notch Transmembrane−intracellular Fragment
OR	Objective Response
ORR	Objective Response Rate
OS	Overall Survival
PARP	Poly (ADP-Ribose) Polymerase
PD	Progressive Disease
PDAC	Pancreatic Ductal Adenocarcinoma
PDGF	Platelet-Derived Growth Factor
PDX	Patient-Derived Xenograft
PEST	Proline (P)/Glutamic Acid (E)/Serine (S)/Threonine (T)-Rich Motif
PFKFB2	6-Phosphofructo-2-Kinase/Fructose-2,6-Biphosphatase 2
PFS	Progression-Free Survival
PHF8	Histone Lysine De-methylase PHF8
PI3K	Phosphatidylinositol-3-kinase
Pofut1	O-fucosyltransferase1
PR	Partial Response
PRC2	Polycomb-Repressive Complex 2
PSEN	Presenilin
PTEN	Phosphatase and Tension Homolog Deleted on Chromosome 10
PTMs	Post-Translational Modifications
RAM	RBP-jκ Associated Molecule
RIN1	RBP-jκ INhibitor-1
RISC	RNA-Induced Silencing Complex
ROS	Reactive Oxygen Species
Rova-T	Rovalpituzumab Tesirine
RBP-jκ	Recombination Signal Binding Protein for Immunoglobulin Kappa J Region
RCT	Randomized Clinical Trial
RT	Radiotherapy
SCC	Squamous Cell Carcinoma
SCF_Fbxw7_	S-phase-Kinase-Associated Protein1(SKP1) Cullin1(CUL1)-F-box
SCLC	Small-Cell Lung Cancer
SD	Stable Disease
SERCA	Sarco(endo)plasmic Reticulum Ca+2 ATPase
SHARP	SMRT/HDAC1-Associated Repressor Protein
siRNA	Silencing RNA
SSCC	Skin Squamous Cell Carcinoma
STAT	SRA Taxonomy Analysis Tool
TAD	Transactivation Domain
T-ALL	T-cell Acute Lymphoblastic Leukemia
TCR	T-cell Receptor
TF	Transcription Factor
TG	Thapsigargin
TGF	Transforming Growth Factor
T-ISC	Tumor-Initiating Stem Cells
TMZ	Temozolomide
TNBC	Triple-Negative Breast Cancer
TS	Thymidylate Synthase
UTX	Ubiquitously Transcribed Tetratricopeptide Repeat X-linked Protein
VEGF	Vascular Endothelial Growth Factor
VEGFR	Vascular Endothelial Growth Factor Receptor
VPA	Valproic Acid
WA	Withaferin A
WT	Wild-Type
XN	Xanthohumol
ZEB1	Zinc Finger E-Box Binding Homeobox 1
