# Supplementary material for: Targeting Notch to Maximize Chemotherapeutic Benefits: Rationale, Advanced Strategies, and Future Perspectives
Source: Cancers (Basel). 2021 Oct 12;13(20):5106. doi: 10.3390/cancers13205106 (PMC8533696; doi:10.3390/cancers13205106)
Supplement: Supplementary file 1 [file cancers-13-05106-s001.zip › cancers-1380938-supplementary.pdf]

Supplementary Material: Targeting Notch to Maximize Chemotherapeutic Benefits: Rationale, Advanced Strategies, and Future Perspectives

Nadezda Zhdanovskaya, Mariarosaria Firrincieli, Sara Lazzari, Eleonora Pace, Pietro Scribani Rossi, Maria Pia Felli, Claudio Talora, Isabella Screpanti * and Rocco Palermo *

Table S1. Principal Notch-inhibiting strategies.

Drug class	Drug Name	Molecular target	Notes on Notch inhibition	References
GSI	RO4929097/R4733	GS	Inhibition of Notch receptors at different concentration. Increased Notch3 cleavage at lower dosages.	[1]
GSI	LY3039478/ JSMD194	GS		
GSI	Nirogacestat/PF-3084014	GS	Higher inhibition of Notch2 than other Notch receptors. Increased Notch3 cleavage at low concentrations	[1]
GSI	AL101/BMS-906024	GS	Equal inhibition of all Notch receptors. Potentiation of Notch3 cleavage at low concentration.	[1]
GSI	AL102/BMS-986115	GS		
GSI	Avagacestat/ BMS-708163	N terminal fragment of PS1. No preferences for PS subtype.	Poor Notch-sparing activity	[2,3]
GSI	MRK-003	GS		
GSI	MRK-560	Higher affinity to GS complex containing PS1 rather than PS2		[4]
GSI	GSI-I	GS		
GSI	DAPT/GSI-IX	Catalytic pocket of PS1.	Potent Notch1 inhibition. Potentiation of Notch3 and Notch4 cleavage at lower concentrations	[1,5]
GSI	GSI-34	GS		
GSI	MK-0752	GS	Equivalent inhibition of Notch1, 2, and 4 cleavage. Increased Notch3 cleavage at low concentration	[1]
GSI	Compound E/GSI-XXI	GS		
GSI	Semagacestat/LY-450139	Catalytic pocket of PS1. No preferences for PS subtype	Potent Notch1 inhibition. Potentiation of Notch3 and Notch4 cleavage at lower concentrations	[1,2,6]
mAb	Tarextumab (OMP-59R5)	Notch2 and Notch3 receptors	Selective Notch2 and Notch3 inhibition at low doses Minimal binding to Notch1 and no binding to Notch4	[7,8]

mAb	Brontictuzumab (OMP-52M51)	Notch receptor negative regulation region (NRR)	Inhibition of activation of Notch1 bearing mutations in HD/PEST and HD/TAD domains	[9,10]
mAb	602.101	EGF-like repeats of Notch1	Inhibits binding of Notch ligands, specific for Notch1	[11]
mAb	Demcizumab (OMP-21M18)	DLL4	Prevents Notch activation by targeting DLL4 ligand	[12]
mAb	Enoticumab (REGN1035)	DLL4	Prevents Notch activation by targeting DLL4 ligand	[13]
mAb	MEDI0639	DLL4	Prevents Notch activation by targeting DLL4 ligand	[14]
Bispecific Ab	HD105	DLL4 and VEGF	Inhibits VEGF/VEGFR2 and DLL4/Notch interaction weaker than single monoclonal antibodies	[15]
Bispecific Ab	HB32	DLL4 and VEGF	Strong inhibition of VEGF and DLL4 simultaneously	[16]
Bispecific Ab	Navixizumab (OMP-305B83)	DLL4 and VEGF	Simultaneous VEGF and DLL4 inhibition	[17]
Bispecific Ab	ABT-165	DLL4 and VEGF	Simultaneous inhibition of VEGF and DLL4 and stronger anti-cancer activity if compared with single Abs	[18]
Bispecific T-cell engager (BiTE)	AMG757 and AMG119	Anti-DLL3 BiTE molecule and CD3 of T-cells	High specificity for DLL3 and capacity to engage human T cells and to initiate the cytotoxic response against tumor cells	[19]
Antibody-drug conjugate	Rovalpituzumab Tesirine	Anti-DLL3 antibody drug conjugate with the DNA-crosslinking agent pyrrolobenzodiazepine and a protease-cleavable linker	Anti-cancer activity in DLL3-expressing cancers	[20,21]
Immunoconjugates	PF-06650808	Anti-Notch3 antibody conjugated to the cytostatic agent auristatin	Selective for Notch3 at low doses	[22]
mAb	15D11	Jagged1	Jagged1 inhibition with minimal toxicity and excellent therapeutic efficacy against bone metastatis	[23]
Hydrocarbon-stapled peptide	SAHM1	MAML1	Prevents assembly of the Notch transcriptional activation complex by inhibiting MAML1 binding	[24]
Synthetic organic compound	IMR-1	MAML1	Prevents assembly of the Notch transcriptional activation complex by inhibiting MAML1 binding	[25]
RBP-jκ Inhibitor-1	RIN1	RBP-jκ	Inhibits transcriptional activation of Notch downstream genes, blocking the association of RBP-jκ with SHARP and NIC	[26]
Pan-Notch inhibitor	CB-103	BTD domain of RBPJ	Inhibits Notch-mediated transcription by interfering with assembly of the NTC	[27,28]

Notch1-selective small-molecule inhibitor	NADI-351	Notch1 transcriptional active complex	Inhibits Notch1-driven transcription by disruption of the NTC	[29]
HES1 inhibitor	JI051	HES1-PHB2 interaction	Inhibition of HES1-mediated transcriptional repression via the PHB2 chaperone	[30]
Natural SERCA inhibitor	Thapsigargin	SERCA pump	Prevents Notch1 receptor maturation through accumulation of unprocessed Notch1 in the ER/Golgi compartment	[31]
Natural SERCA inhibitor	Casearin J	SERCA pump	Inhibition of Notch1 receptor maturation and exocytosis in T-ALL cells carrying *Notch1* HD domain mutations	[32]
Calcium channel blocker	Bepridil	Ca^{2+} channel	Inhibits Notch1 translation, trafficking and/or maturation by reducing ER Ca^{2+} concentration	[33]
Demethylating agent	5-aza-2'deoxycitidine (DAC)	DNA methylation	Reactivation of Notch singling by increasing DLL1 expression in DGC and Notch receptors and targets expression in B-ALL; Inhibition of Notch signaling by enhancing expression of miR-34 in colangiocarcinoma, miR-139-5p in CRC, miR-199 in ovarian cancer	[34–39]
Immunomodulator	Lenalidomide		Inhibition of *Notch2* expression in gastric cancer by enhancing DNA methylation	[40]
Natural compound	Resveratrol		Inhibits *MAML2* by increasing DNA methylation in breast cancer, functional modulation of Notch signaling in several other contexts	[41,42]
HDAC inhibitor	Valproic Acid (VPA)	HDAC4	Reactivation of Notch signaling in NET by increasing H4ac	[43,44]
LSD1 inhibitor	ORY-1001	LSD1	Reactivation of Notch1 in SCLC by indirectly increasing H3K27ac	[45]
JMJD3 inhibitor	GSK-J4	JMJD3	Indirect inhibition of Notch signaling in T-ALL and inhibition of Notch2 expression in CRC by increasing H3K27me3	[46–48]
P300/CBP inhibitor	A485	P300	Indirect inhibition of Notch signaling in T-ALL by reducing H3K27ac	[47]
EZH2 inhibitor	DZNep	EZH2	Suppression of Notch signaling by increasing miR-21-5p and miR-26a-1-5p	[49]
EZH2 inhibitor	GSK343	EZH2	Inhibits Notch signaling by up-regulating miR-21-5p and miR-26a-1-5p	[49]
Nucleic-acid	miR-34 mimic		Targeting Notch1 and/or Jagged1 in breast cancer and CRC	[50–53]
EZH2 inhibitor	GSK-126	EZH2	Inhibition of *Notch1* by increasing miR-34 in cholangiocarcinoma	[35]
Natural compound	delta-tocotrienol		Inhibition of *Notch1* by up-regulating miR-34 in NSCL	[54]
Nucleic-acid	miR-449a mimic		Inhibits Notch in ovarian cancer	[55]
Nucleic-acid	miR-129-5p		Inhibits Notch ligands DLK1 in NLSC	[56]

Pan-HDAC inhibitor	Trichostatin A	HDACs	Inhibition of Notch3 in T-ALL through its hyper-acetylation and consequent proteasomal degradation	[57]
HDAC Inhibitor	Tubacin	HDAC6	Inhibition of Notch3 protein expression and activity in T-ALL	[58]
Pan-HDAC Inhibitor	SAHA/Vorinostat	HDACs	Inhibition of Notch3 expression through its acetylation and consequent proteasomal degradation in urothelial cancer	[59]
HDAC Inhibitor	Apicidin	HDAC1 and HDAC3	Reduction of N1IC protein levels and activity in Notch-dependent leukemic cell lines	[60]
Antioxidant	N-acetylcysteine		Indirect inhibition of Notch2 expression in GBM	[61]
GSK-3β inhibitor	Lithium chloride (LiCl)	GSK-3β	Different effects on Notch stability and activity	[62,63]
EGFR Tyrosine kinase receptor inhibitor	Erlotinib	EGFR kinase	Increase of Notch3 signaling in EGFR-mutated lung cancer cells	[64]
Natural compound	Xanthohumol		Anti-cancer activity through Notch signaling inhibition	[65–69]
Natural compound	Butein		Inhibition of Notch signaling in T-ALL cell lines	[70]
Semisynthetic compound	Chalcone 8		Inhibition of Notch signaling in T-ALL cell lines	[70]
Natural compound	Juglone		Inhibition of Notch3-dependent T-ALL growth through Notch3 signaling downregulation	[71]
Natural compound	Quercetin		Anti-cancer activity through Notch1 signaling inhibition in HCC;<br>Inhibition of Notch signaling in colon cancer	[72,73]
Natural compound	Luteolin		Inhibits Notch1 signaling by regulating miRNAs in breast cancer	[74]
Natural compound	Honokiol	γ-secretase complex	Inhibits Notch2 in melanoma cells and Notch1 in colon cancer by affecting Notch cleavage;	[75,76]
Natural compound	Curcumin		Anti-cancer activity by downregulation of Notch1 signaling in prostate cancer, in osteosarcoma and in oral SCC;<br>Indirect downregulation of Notch1 signaling through EZH2 inhibition in NSCLC	[77–80]
Natural compound	Withaferin A		Inhibition of Notch signaling in colon cancer;<br>Inhibition of Notch3 and Notch1 signaling in ovarian cancer	[81,82]
Natural compound	Cucurbitacin B	ankyrin domain of Notch1	Inhibits Notch1 signaling in colon cancer, by hindering its interaction with important co-factors	[83]
Natural compound	Diallyl trisulfide		Inhibition of Notch signaling in breast cancer	[84]

Natural molecule	ITE	AhR receptor	Reduction of tumor aggressiveness by downregulation of Jagged1-Notch1 activation in TNBC	[85,86]
Natural compound	N-methylhemeanthidine chloride (NMHC)	Hydrophobic pocket of Notch1 NRR	Reactivation of Notch1 signaling by facilitating its proteolytic cleavage and release of the active intracellular domain in AML	[87]
Natural compound	Chrysin		Anti-cancer activity through Notch1 signaling activation in ATC	[88]

Table S2. Clinical trials with GSI as a monotherapy registered at clinicaltrials.gov (accessed on 15 July 2021).

**Agent**	**CT identifier**	**Phase/type**	**Cancer type**	**Results (as of 15 July 2021)**
RO4929097/R4733	NCT01232829	II	Metastatic pancreatic cancer	Yes, published at clinicaltrials.gov (accessed on 14 July 2021)
	NCT01120275	II	Stage IV melanoma	Yes, [89]
	NCT01151449	II	Advanced breast cancer	Yes, published at clinicaltrials.gov (accessed on 14 July 2021)
	NCT01122901	II	Recurrent/progressive glioblastoma	Yes, published at clinicaltrials.gov (accessed on 14 July 2021)
	NCT01217411	I/II	Breast cancer brain metastases	Yes, published at clinicaltrials.gov (accessed on 14 July 2021)
	NCT01269411	I	Recurrent malignant glioma	Yes, published at clinicaltrials.gov (accessed on 15 July 2021)
	NCT01088763	I/II	Refractory solid tumors, CNS tumors, lymphoma, or T-cell leukemia	No
	NCT01096355	I	Metastatic or unresectable solid malignancies	No
	NCT01193868	II	NSCLC	Yes, published at clinicaltrials.gov (accessed on 15 July 2021)

	NCT01175343	II	Recurrent and/or metastatic epithelial ovarian cancer, fallopian tube cancer, or primary peritoneal cancer	Yes, published at clinicaltrials.gov (accessed on 15 July 2021)
	NCT01141569	II	Advanced renal cell carcinoma after the failure of VEGFR therapy	Yes, published at clinicaltrials.gov (accessed on 15 July 2021)
	NCT01116687	II	Metastatic colorectal cancer	Yes, [90]
	NCT01251172	II	Multiple myeloma	No
	NCT01216787	II	Resectable melanoma	No
	NCT01070927	II	Recurrent/refractory NSCLC	No
	NCT01218620	I	Advanced solid tumors	No
	NCT00532090	I	Advanced solid tumors	No
Nirogacestat/PF-03084014	NCT02137564	II	AIDS-associated Kaposi sarcoma	No
	NCT02462707	I	Advanced solid tumors	No
	NCT02338531	II	Triple negative breast cancer	No
	NCT04195399	II	Inoperable desmoid tumors	No
	NCT03785964	III	Desmoid tumor/aggressive fibromatosis	No
	NCT02299635	II	Breast cancer	Yes, published at clinicaltrials.gov (accessed on 15 July 2021)
	NCT01981551	II	Desmoid tumors/aggressive fibromatosis	Yes, [91]
	NCT02955446	I	Desmoid tumor	No
	NCT00878189	I	Advanced cancer and leukemia	Yes, [92]
AL102/BMS-986115	NCT01986218	I	Advanced solid tumors	Yes, [93]
	NCT04871282	II/III	Progressing desmoid tumors	No
LY3039478/JSMD194	NCT02836600	I	Advanced solid tumors	Yes, [94]
	NCT02906618	I	Healthy	No
	NCT02917733	I	Healthy	No
	NCT02659865	I	Healthy	No
AL101/BMS-906024	NCT03691207	II	Adenoid cystic carcinoma	No
	NCT01292655	I	Advanced/metastatic solid tumors	Yes, [95]
	NCT04461600	II	Breast cancer	No
	NCT04111666	I	Healthy	No
MK-0752	NCT00106145	I	Advanced breast cancer	No
	NCT00100152	I	T-ALL (relapsed/refractory)	No
	NCT00572182	I	CNS (relapsed/refractory)	No
	NCT00803894	I	Healthy	No

Table S3. Clinical trials with monoclonal antibodies against Notch receptors and ligands as a monotherapy registered at clinicaltrials.gov (accessed on 19 July 2021).

Agent	CT identifier	Phase/type	Cancer type	Results (as of 19 July 2021)
Tarextumab (OMP-59R5)	NCT01277146	I	Solid tumors	Yes [7]
Brontictuzumab (OMP-52M51)	NCT02662608	n/a	Adenoid cystic carcinoma	Yes, published at clinicaltrials.gov (accessed on 19 July 2021)
	NCT01703572	I	Lymphoid malignancies	No
	NCT01778439	I	Solid tumors	Yes [9]
PF-06650808	NCT02129205	I	Advanced solid tumors	Yes, [22]
Demcizumab (OMP-21M18)	NCT00744562	I	Solid tumors	No
MEDI0639	NCT01577745	I	Solid tumors	Yes, published at clinicaltrials.gov (accessed on 19 July 2021)
Navixizumab (OMP-305B83)	NCT02298387	I	Solid tumors	Yes, [17]
AMG757	NCT04702737	I	Neuroendocrine prostate cancer	No
Rovalpituzumab tesirine (Rova-T)	NCT03543358	II	Cancer	Yes, published at clinicaltrials.gov (accessed on 19 July 2021)
	NCT02674568	II	Relapsed or refractory DLL3-expressing SCLC	Yes, [96]
	NCT03086239	I	Advanced, recurrent SCLC	Yes, [20]
	NCT01901653	I/II	Recurrent SCLC	Yes, [21]
	NCT03503890	N/A	SCLC	No
	NCT02874664	I	SCLC	No
	NCT03334487	III	Relapsed or refractory SCLC	No
	NCT02709889	I/II	Advanced solid tumors	Yes, [97]
AMG119	NCT03392064	I	Relapsed/refractory SCLC	No

References

1. Ran, Y.; Hossain, F.; Pannuti, A.; Lessard, C.B.; Ladd, G.Z.; Jung, J.I.; Minter, L.M.; Osborne, B.A.; Miele, L.; Golde, T.E. gamma-Secretase inhibitors in cancer clinical trials are pharmacologically and functionally distinct. *EMBO Mol Med* **2017**, *9*, 950-966, doi:10.15252/emmm.201607265.
2. Borgegard, T.; Gustavsson, S.; Nilsson, C.; Parpal, S.; Klintenberg, R.; Berg, A.L.; Rosqvist, S.; Serneels, L.; Svensson, S.; Olsson, F.; et al. Alzheimer's disease: presenilin 2-sparing gamma-secretase inhibition is a tolerable Abeta peptide-lowering strategy. *J Neurosci* **2012**, *32*, 17297-17305, doi:10.1523/JNEUROSCI.1451-12.2012.
3. Crump, C.J.; Castro, S.V.; Wang, F.; Pozdnyakov, N.; Ballard, T.E.; Sisodia, S.S.; Bales, K.R.; Johnson, D.S.; Li, Y.M. BMS-708,163 targets presenilin and lacks notch-sparing activity. *Biochemistry* **2012**, *51*, 7209-7211, doi:10.1021/bi301137h.

4. Habets, R.A.; de Bock, C.E.; Serneels, L.; Lodewijckx, I.; Verbeke, D.; Nittner, D.; Narlawar, R.; Demeyer, S.; Dooley, J.; Liston, A.; et al. Safe targeting of T cell acute lymphoblastic leukemia by pathology-specific NOTCH inhibition. *Sci Transl Med* **2019**, *11*, doi:10.1126/scitranslmed.aau6246.
5. Aguayo-Ortiz, R.; Guzman-Ocampo, D.C.; Dominguez, L. Toward the Characterization of DAPT Interactions with gamma-Secretase. *ChemMedChem* **2019**, *14*, 1005-1010, doi:10.1002/cmdc.201900106.
6. Yang, G.; Zhou, R.; Guo, X.; Yan, C.; Lei, J.; Shi, Y. Structural basis of gamma-secretase inhibition and modulation by small molecule drugs. *Cell* **2021**, *184*, 521-533 e514, doi:10.1016/j.cell.2020.11.049.
7. Smith, D.C.; Chugh, R.; Patnaik, A.; Papadopoulos, K.P.; Wang, M.; Kapoun, A.M.; Xu, L.; Dupont, J.; Stagg, R.J.; Tolcher, A. A phase 1 dose escalation and expansion study of Tarextumab (OMP-59R5) in patients with solid tumors. *Invest New Drugs* **2019**, *37*, 722-730, doi:10.1007/s10637-018-0714-6.
8. Yen, W.C.; Fischer, M.M.; Axelrod, F.; Bond, C.; Cain, J.; Cancilla, B.; Henner, W.R.; Meisner, R.; Sato, A.; Shah, J.; et al. Targeting Notch signaling with a Notch2/Notch3 antagonist (tarextumab) inhibits tumor growth and decreases tumor-initiating cell frequency. *Clin Cancer Res* **2015**, *21*, 2084-2095, doi:10.1158/1078-0432.CCR-14-2808.
9. Ferrarotto, R.; Eckhardt, G.; Patnaik, A.; LoRusso, P.; Faoro, L.; Heymach, J.V.; Kapoun, A.M.; Xu, L.; Munster, P. A phase I dose-escalation and dose-expansion study of brontictuzumab in subjects with selected solid tumors. *Ann Oncol* **2018**, *29*, 1561-1568, doi:10.1093/annonc/mdy171.
10. Agnusdei, V.; Minuzzo, S.; Frasson, C.; Grassi, A.; Axelrod, F.; Satyal, S.; Gurney, A.; Hoey, T.; Seganfreddo, E.; Basso, G.; et al. Therapeutic antibody targeting of Notch1 in T-acute lymphoblastic leukemia xenografts. *Leukemia* **2014**, *28*, 278-288, doi:10.1038/leu.2013.183.
11. Sharma, A.; Paranjape, A.N.; Rangarajan, A.; Dighe, R.R. A monoclonal antibody against human Notch1 ligand-binding domain depletes subpopulation of putative breast cancer stem-like cells. *Mol Cancer Ther* **2012**, *11*, 77-86, doi:10.1158/1535-7163.MCT-11-0508.
12. Smith, D.C.; Eisenberg, P.D.; Manikhas, G.; Chugh, R.; Gubens, M.A.; Stagg, R.J.; Kapoun, A.M.; Xu, L.; Dupont, J.; Sikic, B. A phase I dose escalation and expansion study of the anticancer stem cell agent demcizumab (anti-DLL4) in patients with previously treated solid tumors. *Clin Cancer Res* **2014**, *20*, 6295-6303, doi:10.1158/1078-0432.CCR-14-1373.
13. Hu, W.; Lu, C.; Dong, H.H.; Huang, J.; Shen, D.Y.; Stone, R.L.; Nick, A.M.; Shahzad, M.M.; Mora, E.; Jennings, N.B.; et al. Biological roles of the Delta family Notch ligand Dll4 in tumor and endothelial cells in ovarian cancer. *Cancer Res* **2011**, *71*, 6030-6039, doi:10.1158/0008-5472.CAN-10-2719.
14. Jenkins, D.W.; Ross, S.; Veldman-Jones, M.; Foltz, I.N.; Clavette, B.C.; Manchulenko, K.; Eberlein, C.; Kendrew, J.; Petteruti, P.; Cho, S.; et al. MEDI0639: a novel therapeutic antibody targeting Dll4 modulates endothelial cell function and angiogenesis in vivo. *Mol Cancer Ther* **2012**, *11*, 1650-1660, doi:10.1158/1535-7163.MCT-11-1027.
15. Lee, D.; Kim, D.; Choi, Y.B.; Kang, K.; Sung, E.S.; Ahn, J.H.; Goo, J.; Yeom, D.H.; Jang, H.S.; Moon, K.D.; et al. Simultaneous blockade of VEGF and Dll4 by HD105, a bispecific antibody, inhibits tumor progression and angiogenesis. *MAbs* **2016**, *8*, 892-904, doi:10.1080/19420862.2016.1171432.
16. Zhou, R.; Wang, S.; Wen, H.; Wang, M.; Wu, M. The bispecific antibody HB-32, blockade of both VEGF and DLL4 shows potent anti-angiogenic activity in vitro and anti-tumor activity in breast cancer xenograft models. *Exp Cell Res* **2019**, *380*, 141-148, doi:10.1016/j.yexcr.2019.04.025.
17. Jimeno, A.; Moore, K.N.; Gordon, M.; Chugh, R.; Diamond, J.R.; Aljumaily, R.; Mendelson, D.; Kapoun, A.M.; Xu, L.; Stagg, R.; et al. A first-in-human phase 1a study of the bispecific anti-DLL4/anti-VEGF antibody navicixizumab (OMP-305B83) in patients with previously treated solid tumors. *Invest New Drugs* **2019**, *37*, 461-472, doi:10.1007/s10637-018-0665-y.
18. Li, Y.; Hickson, J.A.; Ambrosi, D.J.; Haasch, D.L.; Foster-Duke, K.D.; Eaton, L.J.; DiGiammarino, E.L.; Panchal, S.C.; Jiang, F.; Mudd, S.R.; et al. ABT-165, a Dual Variable Domain Immunoglobulin (DVD-Ig) Targeting DLL4 and VEGF, Demonstrates Superior Efficacy and Favorable Safety Profiles in Preclinical Models. *Mol Cancer Ther* **2018**, *17*, 1039-1050, doi:10.1158/1535-7163.MCT-17-0800.
19. Giffin, M.J.; Cooke, K.; Lobenhofer, E.K.; Estrada, J.; Zhan, J.; Deegen, P.; Thomas, M.; Murawsky, C.M.; Werner, J.; Liu, S.; et al. AMG 757, a Half-Life Extended, DLL3-Targeted Bispecific T-Cell Engager, Shows High Potency and Sensitivity in Preclinical Models of Small-Cell Lung Cancer. *Clin Cancer Res* **2021**, *27*, 1526-1537, doi:10.1158/1078-0432.CCR-20-2845.
20. Udagawa, H.; Akamatsu, H.; Tanaka, K.; Takeda, M.; Kanda, S.; Kirita, K.; Teraoka, S.; Nakagawa, K.; Fujiwara, Y.; Yasuda, I.; et al. Phase I safety and pharmacokinetics study of rovalpituzumab tesirine in Japanese patients with advanced, recurrent small cell lung cancer. *Lung Cancer* **2019**, *135*, 145-150, doi:10.1016/j.lungcan.2019.07.025.
21. Rudin, C.M.; Pietanza, M.C.; Bauer, T.M.; Ready, N.; Morgensztern, D.; Glisson, B.S.; Byers, L.A.; Johnson, M.L.; Burris, H.A., 3rd; Robert, F.; et al. Rovalpituzumab tesirine, a DLL3-targeted antibody-drug conjugate, in recurrent small-cell lung cancer: a first-in-human, first-in-class, open-label, phase 1 study. *Lancet Oncol* **2017**, *18*, 42-51, doi:10.1016/S1470-2045(16)30565-4.
22. Rosen, L.S.; Wesolowski, R.; Baffa, R.; Liao, K.H.; Hua, S.Y.; Gibson, B.L.; Pirie-Shepherd, S.; Tolcher, A.W. A phase I, dose-escalation study of PF-06650808, an anti-Notch3 antibody-drug conjugate, in patients with breast cancer and other advanced solid tumors. *Invest New Drugs* **2020**, *38*, 120-130, doi:10.1007/s10637-019-00754-y.
23. Zheng, H.Q.; Bae, Y.; Kasimir-Bauer, S.; Tang, R.; Chen, J.; Ren, G.W.; Yuan, M.; Esposito, M.; Li, W.Y.; Wei, Y.; et al. Therapeutic Antibody Targeting Tumor- and Osteoblastic Niche-Derived Jagged1 Sensitizes Bone Metastasis to Chemotherapy. *Cancer Cell* **2017**, *32*, 731-+, doi:10.1016/j.ccell.2017.11.002.
24. Moellering, R.E.; Cornejo, M.; Davis, T.N.; Del Bianco, C.; Aster, J.C.; Blacklow, S.C.; Kung, A.L.; Gilliland, D.G.; Verdine, G.L.; Bradner, J.E. Direct inhibition of the NOTCH transcription factor complex. *Nature* **2009**, *462*, 182-188, doi:10.1038/nature08543.

25. Astudillo, L.; Da Silva, T.G.; Wang, Z.; Han, X.; Jin, K.; VanWye, J.; Zhu, X.; Weaver, K.; Oashi, T.; Lopes, P.E.; et al. The Small Molecule IMR-1 Inhibits the Notch Transcriptional Activation Complex to Suppress Tumorigenesis. *Cancer Res* **2016**, *76*, 3593-3603, doi:10.1158/0008-5472.CAN-16-0061.
26. Hurtado, C.; Safarova, A.; Smith, M.; Chung, R.; Bruyneel, A.A.N.; Gomez-Galeno, J.; Oswald, F.; Larson, C.J.; Cashman, J.R.; Ruiz-Lozano, P.; et al. Disruption of NOTCH signaling by a small molecule inhibitor of the transcription factor RBPJ. *Sci Rep* **2019**, *9*, 10811, doi:10.1038/s41598-019-46948-5.
27. Hossain, F.; Majumder, S.; David, J.; Miele, L. Precision Medicine and Triple-Negative Breast Cancer: Current Landscape and Future Directions. *Cancers (Basel)* **2021**, *13*, doi:10.3390/cancers13153739.
28. Lehal, R.; Zaric, J.; Vigolo, M.; Urech, C.; Frismantas, V.; Zangger, N.; Cao, L.; Berger, A.; Chicote, I.; Loubery, S.; et al. Pharmacological disruption of the Notch transcription factor complex. *Proc Natl Acad Sci U S A* **2020**, *117*, 16292-16301, doi:10.1073/pnas.1922606117.
29. Alvarez-Trotta, A.; Guerrant, W.; Astudillo, L.; Lahiry, M.; Diluvio, G.; Shersher, E.; Kaneku, H.; Robbins, D.J.; Orton, D.; Capobianco, A.J. Pharmacological Disruption of the Notch1 Transcriptional Complex Inhibits Tumor Growth by Selectively Targeting Cancer Stem Cells. *Cancer Res* **2021**, *81*, 3347-3357, doi:10.1158/0008-5472.CAN-20-3611.
30. Perron, A.; Nishikawa, Y.; Iwata, J.; Shimojo, H.; Takaya, J.; Kobayashi, K.; Imayoshi, I.; Mbenza, N.M.; Takenoya, M.; Kageyama, R.; et al. Small-molecule screening yields a compound that inhibits the cancer-associated transcription factor Hes1 via the PHB2 chaperone. *J Biol Chem* **2018**, *293*, 8285-8294, doi:10.1074/jbc.RA118.002316.
31. Roti, G.; Carlton, A.; Ross, K.N.; Markstein, M.; Pajcini, K.; Su, A.H.; Perrimon, N.; Pear, W.S.; Kung, A.L.; Blacklow, S.C.; et al. Complementary genomic screens identify SERCA as a therapeutic target in NOTCH1 mutated cancer. *Cancer Cell* **2013**, *23*, 390-405, doi:10.1016/j.ccr.2013.01.015.
32. De Ford, C.; Heidersdorf, B.; Haun, F.; Murillo, R.; Friedrich, T.; Borner, C.; Merfort, I. The clerodane diterpene casearin J induces apoptosis of T-ALL cells through SERCA inhibition, oxidative stress, and interference with Notch1 signaling. *Cell Death Dis* **2016**, *7*, e2070, doi:10.1038/cddis.2015.413.
33. Baldoni, S.; Del Papa, B.; Dorillo, E.; Aureli, P.; De Falco, F.; Rompietti, C.; Sorcini, D.; Varasano, E.; Cecchini, D.; Zei, T.; et al. Bepridil exhibits anti-leukemic activity associated with NOTCH1 pathway inhibition in chronic lymphocytic leukemia. *Int J Cancer* **2018**, *143*, 958-970, doi:10.1002/ijc.31355.
34. Kuang, S.Q.; Fang, Z.; Zweidler-McKay, P.A.; Yang, H.; Wei, Y.; Gonzalez-Cervantes, E.A.; Boumber, Y.; Garcia-Manero, G. Epigenetic inactivation of Notch-Hes pathway in human B-cell acute lymphoblastic leukemia. *PLoS One* **2013**, *8*, e61807, doi:10.1371/journal.pone.0061807.
35. Kwon, H.; Song, K.; Han, C.; Zhang, J.; Lu, L.; Chen, W.; Wu, T. Epigenetic Silencing of miRNA-34a in Human Cholangiocarcinoma via EZH2 and DNA Methylation: Impact on Regulation of Notch Pathway. *Am J Pathol* **2017**, *187*, 2288-2299, doi:10.1016/j.ajpath.2017.06.014.
36. Liu, M.X.; Siu, M.K.; Liu, S.S.; Yam, J.W.; Ngan, H.Y.; Chan, D.W. Epigenetic silencing of microRNA-199b-5p is associated with acquired chemoresistance via activation of JAG1-Notch1 signaling in ovarian cancer. *Oncotarget* **2014**, *5*, 944-958, doi:10.18632/oncotarget.1458.
37. Piazzi, G.; Fini, L.; Selgrad, M.; Garcia, M.; Daoud, Y.; Wex, T.; Malfertheiner, P.; Gasbarrini, A.; Romano, M.; Meyer, R.L.; et al. Epigenetic regulation of Delta-Like1 controls Notch1 activation in gastric cancer. *Oncotarget* **2011**, *2*, 1291-1301, doi:10.18632/oncotarget.414.
38. Xu, K.; Shen, K.; Liang, X.; Li, Y.; Nagao, N.; Li, J.; Liu, J.; Yin, P. MiR-139-5p reverses CD44+/CD133+-associated multidrug resistance by downregulating NOTCH1 in colorectal carcinoma cells. *Oncotarget* **2016**, *7*, 75118-75129, doi:10.18632/oncotarget.12611.
39. Zhang, L.; Dong, Y.; Zhu, N.; Tsoi, H.; Zhao, Z.; Wu, C.W.; Wang, K.; Zheng, S.; Ng, S.S.; Chan, F.K.; et al. microRNA-139-5p exerts tumor suppressor function by targeting NOTCH1 in colorectal cancer. *Mol Cancer* **2014**, *13*, 124, doi:10.1186/1476-4598-13-124.
40. Ding, W.; Zeng, T.; Tao, W.; Ge, W.; Deng, J.; Lei, H.; Xiao, Y.; Liao, F. Effect of lenalidomide on the human gastric cancer cell line SGC7901/vincristine Notch signaling. *J Cancer Res Ther* **2018**, *14*, S237-S242, doi:10.4103/0973-1482.183181.
41. Farooqi, A.A.; Khalid, S.; Ahmad, A. Regulation of Cell Signaling Pathways and miRNAs by Resveratrol in Different Cancers. *Int J Mol Sci* **2018**, *19*, doi:10.3390/ijms19030652.
42. Lubecka, K.; Kurzava, L.; Flower, K.; Buvala, H.; Zhang, H.; Teegarden, D.; Camarillo, I.; Suderman, M.; Kuang, S.; Andrisani, O.; et al. Stilbenoids remodel the DNA methylation patterns in breast cancer cells and inhibit oncogenic NOTCH signaling through epigenetic regulation of MAML2 transcriptional activity. *Carcinogenesis* **2016**, *37*, 656-668, doi:10.1093/carcin/bgw048.
43. Sun, L.; He, Q.; Tsai, C.; Lei, J.; Chen, J.; Vienna Makcey, L.; Coy, D.H. HDAC inhibitors suppressed small cell lung cancer cell growth and enhanced the suppressive effects of receptor-targeting cytotoxins via upregulating somatostatin receptor II. *Am J Transl Res* **2018**, *10*, 545-553.
44. Sun, L.; Qian, Q.; Sun, G.; Mackey, L.V.; Fuselier, J.A.; Coy, D.H.; Yu, C.Y. Valproic acid induces NET cell growth arrest and enhances tumor suppression of the receptor-targeted peptide-drug conjugate via activating somatostatin receptor type II. *J Drug Target* **2016**, *24*, 169-177, doi:10.3109/1061186X.2015.1066794.
45. Augert, A.; Eastwood, E.; Ibrahim, A.H.; Wu, N.; Grunblatt, E.; Basom, R.; Liggitt, D.; Eaton, K.D.; Martins, R.; Poirier, J.T.; et al. Targeting NOTCH activation in small cell lung cancer through LSD1 inhibition. *Sci Signal* **2019**, *12*, doi:10.1126/scisignal.aau2922.

46. Ntziachristos, P.; Tsirigos, A.; Welstead, G.G.; Trimarchi, T.; Bakogianni, S.; Xu, L.; Loizou, E.; Holmfeldt, L.; Strikoudis, A.; King, B.; et al. Contrasting roles of histone 3 lysine 27 demethylases in acute lymphoblastic leukaemia. *Nature* **2014**, *514*, 513-517, doi:10.1038/nature13605.
47. Tottone, L.; Zhdanovskaya, N.; Carmona Pestana, A.; Zampieri, M.; Simeoni, F.; Lazzari, S.; Ruocco, V.; Pelullo, M.; Caiafa, P.; Felli, M.P.; et al. Histone Modifications Drive Aberrant Notch3 Expression/Activity and Growth in T-ALL. *Front Oncol* **2019**, *9*, 198, doi:10.3389/fonc.2019.00198.
48. Wang, Q.; Chen, X.; Jiang, Y.; Liu, S.; Liu, H.; Sun, X.; Zhang, H.; Liu, Z.; Tao, Y.; Li, C.; et al. Elevating H3K27me3 level sensitizes colorectal cancer to oxaliplatin. *J Mol Cell Biol* **2020**, *12*, 125-137, doi:10.1093/jmcb/mjz032.
49. Wang, S.; Cai, L.; Zhang, F.; Shang, X.; Xiao, R.; Zhou, H. Inhibition of EZH2 Attenuates Sorafenib Resistance by Targeting NOTCH1 Activation-Dependent Liver Cancer Stem Cells via NOTCH1-Related MicroRNAs in Hepatocellular Carcinoma. *Transl Oncol* **2020**, *13*, 100741, doi:10.1016/j.tranon.2020.01.002.
50. Bu, P.; Chen, K.Y.; Chen, J.H.; Wang, L.; Walters, J.; Shin, Y.J.; Goerger, J.P.; Sun, J.; Witherspoon, M.; Rakhilin, N.; et al. A microRNA miR-34a-regulated bimodal switch targets Notch in colon cancer stem cells. *Cell Stem Cell* **2013**, *12*, 602-615, doi:10.1016/j.stem.2013.03.002.
51. Kang, L.; Mao, J.; Tao, Y.; Song, B.; Ma, W.; Lu, Y.; Zhao, L.; Li, J.; Yang, B.; Li, L. MicroRNA-34a suppresses the breast cancer stem cell-like characteristics by downregulating Notch1 pathway. *Cancer Sci* **2015**, *106*, 700-708, doi:10.1111/cas.12656.
52. Li, X.J.; Ji, M.H.; Zhong, S.L.; Zha, Q.B.; Xu, J.J.; Zhao, J.H.; Tang, J.H. MicroRNA-34a modulates chemosensitivity of breast cancer cells to adriamycin by targeting Notch1. *Arch Med Res* **2012**, *43*, 514-521, doi:10.1016/j.arcmed.2012.09.007.
53. Park, E.Y.; Chang, E.; Lee, E.J.; Lee, H.W.; Kang, H.G.; Chun, K.H.; Woo, Y.M.; Kong, H.K.; Ko, J.Y.; Suzuki, H.; et al. Targeting of miR34a-NOTCH1 axis reduced breast cancer stemness and chemoresistance. *Cancer Res* **2014**, *74*, 7573-7582, doi:10.1158/0008-5472.CAN-14-1140.
54. Ji, X.; Wang, Z.; Geamanu, A.; Goja, A.; Sarkar, F.H.; Gupta, S.V. Delta-tocotrienol suppresses Notch-1 pathway by upregulating miR-34a in nonsmall cell lung cancer cells. *Int J Cancer* **2012**, *131*, 2668-2677, doi:10.1002/ijc.27549.
55. Zhou, Y.; Chen, Q.; Qin, R.; Zhang, K.; Li, H. MicroRNA-449a reduces cell survival and enhances cisplatin-induced cytotoxicity via downregulation of NOTCH1 in ovarian cancer cells. *Tumour Biol* **2014**, *35*, 12369-12378, doi:10.1007/s13277-014-2551-3.
56. Ma, Z.; Cai, H.; Zhang, Y.; Chang, L.; Cui, Y. MiR-129-5p inhibits non-small cell lung cancer cell stemness and chemoresistance through targeting DLK1. *Biochem Biophys Res Commun* **2017**, *490*, 309-316, doi:10.1016/j.bbrc.2017.06.041.
57. Palermo, R.; Checquolo, S.; Giovenco, A.; Grazioli, P.; Kumar, V.; Campese, A.F.; Giorgi, A.; Napolitano, M.; Canettieri, G.; Ferrara, G.; et al. Acetylation controls Notch3 stability and function in T-cell leukemia. *Oncogene* **2012**, *31*, 3807-3817, doi:10.1038/onc.2011.533.
58. Pinazza, M.; Ghisi, M.; Minuzzo, S.; Agnusdei, V.; Fossati, G.; Ciminale, V.; Pezze, L.; Ciribilli, Y.; Pilotto, G.; Venturoli, C.; et al. Histone deacetylase 6 controls Notch3 trafficking and degradation in T-cell acute lymphoblastic leukemia cells. *Oncogene* **2018**, *37*, 3839-3851, doi:10.1038/s41388-018-0234-z.
59. Zhang, H.; Liu, L.; Liu, C.; Pan, J.; Lu, G.; Zhou, Z.; Chen, Z.; Qian, C. Notch3 overexpression enhances progression and chemoresistance of urothelial carcinoma. *Oncotarget* **2017**, *8*, 34362-34373, doi:10.18632/oncotarget.16156.
60. Ferrante, F.; Giaimo, B.D.; Bartkuhn, M.; Zimmermann, T.; Close, V.; Mertens, D.; Nist, A.; Stiewe, T.; Meier-Soelch, J.; Kracht, M.; et al. HDAC3 functions as a positive regulator in Notch signal transduction. *Nucleic Acids Res* **2020**, *48*, 3496-3512, doi:10.1093/nar/gkaa088.
61. Deng, J.; Liu, A.D.; Hou, G.Q.; Zhang, X.; Ren, K.; Chen, X.Z.; Li, S.S.C.; Wu, Y.S.; Cao, X. N-acetylcysteine decreases malignant characteristics of glioblastoma cells by inhibiting Notch2 signaling. *J Exp Clin Cancer Res* **2019**, *38*, 2, doi:10.1186/s13046-018-1016-8.
62. Foltz, D.R.; Santiago, M.C.; Berechid, B.E.; Nye, J.S. Glycogen synthase kinase-3beta modulates notch signaling and stability. *Curr Biol* **2002**, *12*, 1006-1011, doi:10.1016/s0960-9822(02)00888-6.
63. Jin, Y.H.; Kim, H.; Oh, M.; Ki, H.; Kim, K. Regulation of Notch1/NICD and Hes1 expressions by GSK-3alpha/beta. *Mol Cells* **2009**, *27*, 15-19, doi:10.1007/s10059-009-0001-7.
64. Arasada, R.R.; Amann, J.M.; Rahman, M.A.; Huppert, S.S.; Carbone, D.P. EGFR blockade enriches for lung cancer stem-like cells through Notch3-dependent signaling. *Cancer Res* **2014**, *74*, 5572-5584, doi:10.1158/0008-5472.CAN-13-3724.
65. Drenzek, J.G.; Seiler, N.L.; Jaskula-Sztul, R.; Rausch, M.M.; Rose, S.L. Xanthohumol decreases Notch1 expression and cell growth by cell cycle arrest and induction of apoptosis in epithelial ovarian cancer cell lines. *Gynecol Oncol* **2011**, *122*, 396-401, doi:10.1016/j.ygyno.2011.04.027.
66. Kunnimalaiyaan, S.; Sokolowski, K.M.; Balamurugan, M.; Gamblin, T.C.; Kunnimalaiyaan, M. Xanthohumol inhibits Notch signaling and induces apoptosis in hepatocellular carcinoma. *PLoS One* **2015**, *10*, e0127464, doi:10.1371/journal.pone.0127464.
67. Kunnimalaiyaan, S.; Trevino, J.; Tsai, S.; Gamblin, T.C.; Kunnimalaiyaan, M. Xanthohumol-Mediated Suppression of Notch1 Signaling Is Associated with Antitumor Activity in Human Pancreatic Cancer Cells. *Mol Cancer Ther* **2015**, *14*, 1395-1403, doi:10.1158/1535-7163.MCT-14-0915.
68. Monteghirfo, S.; Tosetti, F.; Ambrosini, C.; Stigliani, S.; Pozzi, S.; Frassoni, F.; Fassina, G.; Soverini, S.; Albini, A.; Ferrari, N. Antileukemia effects of xanthohumol in Bcr/Abl-transformed cells involve nuclear factor-kappaB and p53 modulation. *Mol Cancer Ther* **2008**, *7*, 2692-2702, doi:10.1158/1535-7163.MCT-08-0132.
69. Sun, Z.; Zhou, C.; Liu, F.; Zhang, W.; Chen, J.; Pan, Y.; Ma, L.; Liu, Q.; Du, Y.; Yang, J.; et al. Inhibition of breast cancer cell survival by Xanthohumol via modulation of the Notch signaling pathway in vivo and in vitro. *Oncol Lett* **2018**, *15*, 908-916, doi:10.3892/ol.2017.7434.

70. Mori, M.; Tottone, L.; Quaglio, D.; Zhdanovskaya, N.; Ingallina, C.; Fusto, M.; Ghirga, F.; Peruzzi, G.; Crestoni, M.E.; Simeoni, F.; et al. Identification of a novel chalcone derivative that inhibits Notch signaling in T-cell acute lymphoblastic leukemia. *Sci Rep* **2017**, *7*, 2213, doi:10.1038/s41598-017-02316-9.
71. Giuli, M.V.; Diluvio, G.; Giuliani, E.; Franciosa, G.; Di Magno, L.; Pignataro, M.G.; Tottone, L.; Nicoletti, C.; Besharat, Z.M.; Peruzzi, G.; et al. Notch3 contributes to T-cell leukemia growth via regulation of the unfolded protein response. *Oncogenesis* **2020**, *9*, 93, doi:10.1038/s41389-020-00279-7.
72. Li, Y.; Wang, Z.; Jin, J.; Zhu, S.X.; He, G.Q.; Li, S.H.; Wang, J.; Cai, Y. Quercetin pretreatment enhances the radiosensitivity of colon cancer cells by targeting Notch-1 pathway. *Biochem Biophys Res Commun* **2020**, *523*, 947-953, doi:10.1016/j.bbrc.2020.01.048.
73. Salama, Y.A.; El-Karef, A.; El Gayyar, A.M.; Abdel-Rahman, N. Beyond its antioxidant properties: Quercetin targets multiple signalling pathways in hepatocellular carcinoma in rats. *Life Sci* **2019**, *236*, 116933, doi:10.1016/j.lfs.2019.116933.
74. Sun, D.W.; Zhang, H.D.; Mao, L.; Mao, C.F.; Chen, W.; Cui, M.; Ma, R.; Cao, H.X.; Jing, C.W.; Wang, Z.; et al. Luteolin Inhibits Breast Cancer Development and Progression In Vitro and In Vivo by Suppressing Notch Signaling and Regulating MiRNAs. *Cell Physiol Biochem* **2015**, *37*, 1693-1711, doi:10.1159/000438535.
75. Kaushik, G.; Venugopal, A.; Ramamoorthy, P.; Standing, D.; Subramaniam, D.; Umar, S.; Jensen, R.A.; Anant, S.; Mammen, J.M. Honokiol inhibits melanoma stem cells by targeting notch signaling. *Mol Carcinog* **2015**, *54*, 1710-1721, doi:10.1002/mc.22242.
76. Ponnurangam, S.; Mammen, J.M.; Ramalingam, S.; He, Z.; Zhang, Y.; Umar, S.; Subramaniam, D.; Anant, S. Honokiol in combination with radiation targets notch signaling to inhibit colon cancer stem cells. *Mol Cancer Ther* **2012**, *11*, 963-972, doi:10.1158/1535-7163.MCT-11-0999.
77. Li, Y.; Zhang, J.; Ma, D.; Zhang, L.; Si, M.; Yin, H.; Li, J. Curcumin inhibits proliferation and invasion of osteosarcoma cells through inactivation of Notch-1 signaling. *FEBS J* **2012**, *279*, 2247-2259, doi:10.1111/j.1742-4658.2012.08607.x.
78. Liao, S.; Xia, J.; Chen, Z.; Zhang, S.; Ahmad, A.; Miele, L.; Sarkar, F.H.; Wang, Z. Inhibitory effect of curcumin on oral carcinoma CAL-27 cells via suppression of Notch-1 and NF-kappaB signaling pathways. *J Cell Biochem* **2011**, *112*, 1055-1065, doi:10.1002/jcb.23019.
79. Sha, J.; Li, J.; Wang, W.; Pan, L.; Cheng, J.; Li, L.; Zhao, H.; Lin, W. Curcumin induces G0/G1 arrest and apoptosis in hormone independent prostate cancer DU-145 cells by down regulating Notch signaling. *Biomed Pharmacother* **2016**, *84*, 177-184, doi:10.1016/j.biopha.2016.09.037.
80. Wu, G.Q.; Chai, K.Q.; Zhu, X.M.; Jiang, H.; Wang, X.; Xue, Q.; Zheng, A.H.; Zhou, H.Y.; Chen, Y.; Chen, X.C.; et al. Anti-cancer effects of curcumin on lung cancer through the inhibition of EZH2 and NOTCH1. *Oncotarget* **2016**, *7*, 26535-26550, doi:10.18632/oncotarget.8532.
81. Koduru, S.; Kumar, R.; Srinivasan, S.; Evers, M.B.; Damodaran, C. Notch-1 inhibition by Withaferin-A: a therapeutic target against colon carcinogenesis. *Mol Cancer Ther* **2010**, *9*, 202-210, doi:10.1158/1535-7163.MCT-09-0771.
82. Zhang, X.; Samadi, A.K.; Roby, K.F.; Timmermann, B.; Cohen, M.S. Inhibition of cell growth and induction of apoptosis in ovarian carcinoma cell lines CaOV3 and SKOV3 by natural withanolide Withaferin A. *Gynecol Oncol* **2012**, *124*, 606-612, doi:10.1016/j.ygyno.2011.11.044.
83. Dandawate, P.; Subramaniam, D.; Panovich, P.; Standing, D.; Krishnamachary, B.; Kaushik, G.; Thomas, S.M.; Dhar, A.; Weir, S.J.; Jensen, R.A.; et al. Cucurbitacin B and I inhibits colon cancer growth by targeting the Notch signaling pathway. *Sci Rep* **2020**, *10*, 1290, doi:10.1038/s41598-020-57940-9.
84. Elsherbiny, N.M.; El-Sherbiny, M.; Zaitone, S.A. Diallyl trisulfide potentiates chemotherapeutic efficacy of doxorubicin in experimentally induced mammary carcinoma: Role of Notch signaling. *Pathol Res Pract* **2020**, *216*, 153139, doi:10.1016/j.prp.2020.153139.
85. Paris, A.; Tardif, N.; Galibert, M.D.; Corre, S. AhR and Cancer: From Gene Profiling to Targeted Therapy. *Int J Mol Sci* **2021**, *22*, doi:10.3390/ijms22020752.
86. Xiu, M.X.; Liu, Y.M.; Kuang, B.H. The oncogenic role of Jagged1/Notch signaling in cancer. *Biomed Pharmacother* **2020**, *129*, 110416, doi:10.1016/j.biopha.2020.110416.
87. Ye, Q.; Jiang, J.; Zhan, G.; Yan, W.; Huang, L.; Hu, Y.; Su, H.; Tong, Q.; Yue, M.; Li, H.; et al. Small molecule activation of NOTCH signaling inhibits acute myeloid leukemia. *Sci Rep* **2016**, *6*, 26510, doi:10.1038/srep26510.
88. Yu, X.M.; Phan, T.; Patel, P.N.; Jaskula-Sztul, R.; Chen, H. Chrysin activates Notch1 signaling and suppresses tumor growth of anaplastic thyroid carcinoma in vitro and in vivo. *Cancer* **2013**, *119*, 774-781, doi:10.1002/cncr.27742.
89. Lee, S.M.; Moon, J.; Redman, B.G.; Chidiac, T.; Flaherty, L.E.; Zha, Y.; Othus, M.; Ribas, A.; Sondak, V.K.; Gajewski, T.F.; et al. Phase 2 study of RO4929097, a gamma-secretase inhibitor, in metastatic melanoma: SWOG 0933. *Cancer* **2015**, *121*, 432-440, doi:10.1002/cncr.29055.
90. Strosberg, J.R.; Yeatman, T.; Weber, J.; Coppola, D.; Schell, M.J.; Han, G.; Almhanna, K.; Kim, R.; Valone, T.; Jump, H.; et al. A phase II study of RO4929097 in metastatic colorectal cancer. *Eur J Cancer* **2012**, *48*, 997-1003, doi:10.1016/j.ejca.2012.02.056.
91. Kummar, S.; O'Sullivan Coyne, G.; Do, K.T.; Turkbey, B.; Meltzer, P.S.; Polley, E.; Choyke, P.L.; Meehan, R.; Vilimas, R.; Horneffer, Y.; et al. Clinical Activity of the gamma-Secretase Inhibitor PF-03084014 in Adults With Desmoid Tumors (Aggressive Fibromatosis). *J Clin Oncol* **2017**, *35*, 1561-1569, doi:10.1200/JCO.2016.71.1994.
92. Papayannidis, C.; DeAngelo, D.J.; Stock, W.; Huang, B.; Shaik, M.N.; Cesari, R.; Zheng, X.; Reynolds, J.M.; English, P.A.; Ozeck, M.; et al. A Phase 1 study of the novel gamma-secretase inhibitor PF-03084014 in patients with T-cell acute lymphoblastic leukemia and T-cell lymphoblastic lymphoma. *Blood Cancer J* **2015**, *5*, e350, doi:10.1038/bcj.2015.80.
93. Aung, K.L.; El-Khoueiry, A.B.; Gelmon, K.; Tran, B.; Bajaj, G.; He, B.; Chen, T.; Zhu, L.; Poojary, S.; Basak, S.; et al. A multi-arm phase I dose escalating study of an oral NOTCH inhibitor BMS-986115 in patients with advanced solid tumours. *Invest New Drugs* **2018**, *36*, 1026-1036, doi:10.1007/s10637-018-0597-6.

94. Doi, T.; Tajimi, M.; Mori, J.; Asou, H.; Inoue, K.; Benhadji, K.A.; Naito, Y. A phase 1 study of crenigacestat (LY3039478), the Notch inhibitor, in Japanese patients with advanced solid tumors. *Invest New Drugs* **2021**, *39*, 469-476, doi:10.1007/s10637-020-01001-5.
95. El-Khoueiry, A.B.; Desai, J.; Iyer, S.P.; Gadgeel, S.M.; Ramalingam, S.S.; Horn, L.; LoRusso, P.; Bajaj, G.; Kollia, G.; Qi, Z. A phase I study of AL101, a pan-NOTCH inhibitor, in patients (pts) with locally advanced or metastatic solid tumors. **2018**.
96. Morgensztern, D.; Besse, B.; Greillier, L.; Santana-Davila, R.; Ready, N.; Hann, C.L.; Glisson, B.S.; Farago, A.F.; Dowlati, A.; Rudin, C.M.; et al. Efficacy and Safety of Rovalpituzumab Tesirine in Third-Line and Beyond Patients with DLL3-Expressing, Relapsed/Refractory Small-Cell Lung Cancer: Results From the Phase II TRINITY Study. *Clin Cancer Res* **2019**, *25*, 6958-6966, doi:10.1158/1078-0432.CCR-19-1133.
97. Xie, H.; Kaye, F.J.; Isse, K.; Sun, Y.; Ramoth, J.; French, D.M.; Flotte, T.J.; Luo, Y.; Saunders, L.R.; Mansfield, A.S. Delta-Like Protein 3 Expression and Targeting in Merkel Cell Carcinoma. *Oncologist* **2020**, *25*, 810-817, doi:10.1634/theoncologist.2019-0877.
